# Solid-Phase Synthesis of Glycosyl Phosphate Repeating Units via Glycosyl Boranophosphates as Stable Intermediates

**DOI:** 10.1021/acs.orglett.3c01293

**Published:** 2023-05-19

**Authors:** Kazuki Sato, Kazumasa Muramoto, Tomoya Hagio, Rintaro I. Hara, Takeshi Wada

**Affiliations:** † Department of Medicinal and Life Sciences, Faculty of Pharmaceutical Sciences, Tokyo University of Science, 2641 Yamazaki, Noda, Chiba 278-8510, Japan; ‡ Department of Neurology and Neurological Science, Graduate School of Medicinal and Dental Sciences, Tokyo Medical and Dental University, 1-5-45 Yushima, Bunkyo-ku, Tokyo 113-8519, Japan

## Abstract

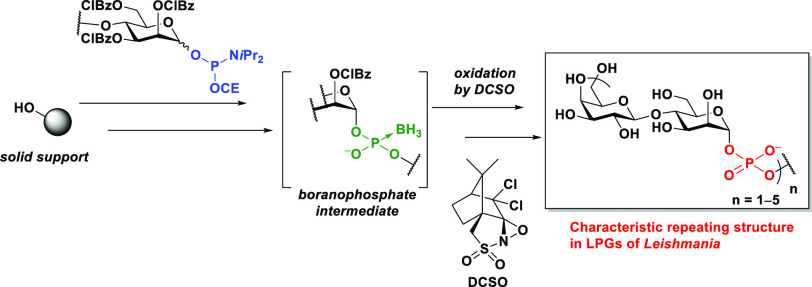

Solid-phase synthesis of glycosyl phosphate repeating units was investigated using glycosyl boranophosphates as stable precursors. The stable nature of glycosyl boranophosphate enables the elongation of a saccharide chain without remarkable decomposition. After deprotection of the boranophosphotriester linkages to boranophosphodiesters, the intersugar linkages were converted to the phosphate counterparts quantitatively using an oxaziridine derivative. This method significantly improves the synthesis of oligosaccharides containing glycosyl phosphate units.

*Leishmania* is a tropical protozoan parasite that causes Leishmaniasis.^1^ Leishmaniasis is one of the neglected tropical diseases, and although there is a few therapeutics for Leishmaniasis,^1^ a fundamental means to eradicate it is highly required. Developing a vaccine against *Leishmania* is promising, and lipophosphoglycans (LPGs) on the *Leishmania* surface^2^ are reported to play a vital role in its survival and infectious processes.^3^ Notably, purified LPGs have been used as a vaccine against *Leishmania major*.^4^ Synthetic phosphoglycans conjugated with tetanus toxin fragment C protein have also shown significant protection against *Leishmania* infection in mice.^5^ LPGs have a characteristic repeating unit of [→6)β-d-Gal-(1 → 4)-α-d-Man-(1-P)]^2^ (Figure 1). The length of the repeating units varies from 15 to 30 in accordance with the life stage of *Leishmania*.^6^ Therefore, there is a need for a method to synthesize phosphoglycans with structural homogeneity to progress in this field.

**Figure 1:**
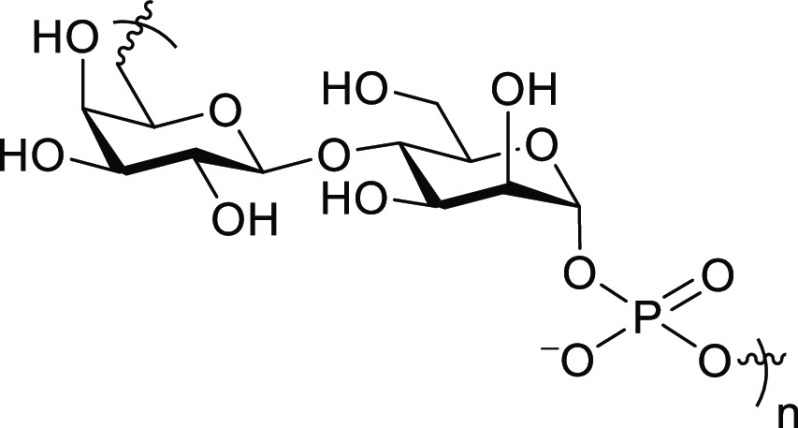
Structure of the glycosyl phosphate repeating units found in the LPGs of *Leishmania donovani*.

The traditional method of synthesizing glycosyl phosphates involves using the *H*-phosphonate method, where a glycosyl *H*-phosphonate monoester monomer unit is condensed with a hydroxy group of another molecule in the presence of a condensing reagent such as pivaloyl chloride (PivCl) and the resultant *H*-phosphonate diester is further oxidized to form its phosphodiester counterpart.^7^ However, it has been reported that the oxidation of *H*-phosphonate diester is prone to side reactions such as hydrolysis of the intersugar linkages.^8,9^ In addition, the phosphodiester can be a potential reaction site and may react with a condensing reagent, which causes undesired reactions. Although some reports have attempted to synthesize repeating units using a polycondensation approach,^10,11^ the length of the repeating units prepared stepwise is limited to four.^12^ Recently, Zhang et al. reported the α-selective synthesis of glycosyl phosphate using glycosyl *o*-alkynylbenzoate and phosphate as the glycosyl donor and acceptor, respectively, in the presence of a gold(I) catalyst. They effectively constructed intersugar phosphate linkages with good stereoselectivity.^13^ Another approach is to use a glycosyl phosphoramidite monomer, which can be condensed with a hydroxy group to form a phosphite triester and then oxidized to a phosphotriester.^7^ In nucleic acid chemistry, this method is the most widely used due to the high coupling efficiency.^14^ However, as one can see from the fact that glycosyl phosphotriesters and phosphite triesters are used as potent glycosyl donors under acidic conditions,^15,16^ the liability of these moieties should be taken into consideration for the synthesis of glycosyl phosphate derivatives. To address this issue, our group has shown that the substitution of a 2-position hydroxy group with a fluorine atom prevents the degradation of glycosyl phosphates and phosphites, probably due to its electron-withdrawing nature, which prohibits the formation of oxocarbenium cations.^17^ In this study, two strategies were developed to synthesize glycosyl phosphate repeating units in LPGs of *Leishmania*: (1) introduction of the electron-withdrawing group as a hydroxy protecting group and (2) utilization of glycosyl boranophosphates as stable precursors of glycosyl phosphates. As for (1), the preliminary investigation revealed that the use of the benzoyl (Bz) protecting group did not prevent the decomposition of a glycosyl phosphite and/or phosphate (data not shown). Thus, the *o*-chlorobenzoyl group was chosen as a strong electron-withdrawing group. Regarding (2), Prosperi et al.^18^ and our group^19^ have shown that a glycosyl boranophosphate, in which one of nonbridging oxygen atoms of a phosphate is replaced with a borano group, has substantial stability under acidic conditions. Additionally, glycosyl boranophosphodiesters were converted to phosphodiesters via either *H*-phosphonate^19−21^ or acyl phosphite intermediates.^22^ Therefore, we anticipated an efficient synthesis of oligo-(glycosyl phosphate) would be possible by creating boranophosphotriester intersugar linkages and converting the linkages to the corresponding phosphodiester linkages in the final stage of the synthesis.

At first, we started to synthesize a monomer for the construction of the repeating units. The hydroxy groups of the mannose residue were protected by *o*-chlorobenzoyl groups, while those of the galactose residue were protected by Bz groups except for the 6-position, which had an *o*-(4-methoxytrityl) group. The phosphoramidite monomer **2** was obtained by the phosphitylation of the hemiacetal **1** with the preference for an α-isomer (α/β = 89:11–94:6, Scheme 1). The configuration of the anomeric position was confirmed by the ^1^*J*_C1–H1_ value (172 Hz) of **2**.^23^ The detailed experimental procedures are described in the Supporting Information.

**Scheme 1:**
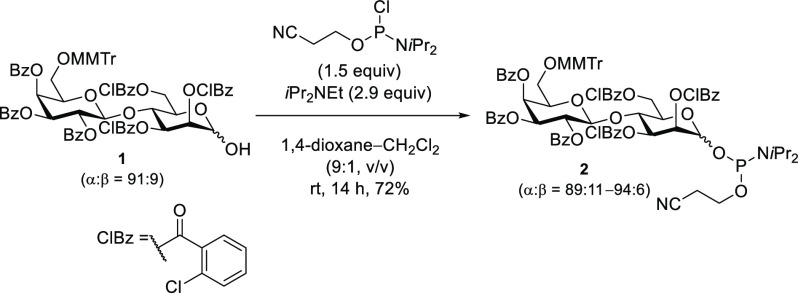
Synthesis of Disaccharide 1-Phosphoramidite **2**

Next, the solid-phase synthesis of disaccharide 1-phosphate was initiated (Scheme 2). Controlled pore glass (CPG) was chosen as a solid support, and the 1,4-bis(2-hydroxyethyl) hydroquinone spacer, which enables the detection of products with its UV absorption, was introduced via a succinyl linker to CPG.^17^ The hydroxy group of **3** was condensed with phosphoramidite **2** in MeCN in the presence of 4,5-dicyanoimidazole (DCI) as an acidic activator for 10 min. The resulting phosphite was oxidized to its phosphotriester counterpart using (+)-(8,8-dichlorocamphorylsulfonyl)oxaziridine (DCSO). After the MMTr group removal at the 6-position of the galactose residue and the deprotection of the phosphate moiety with Et_3_N, the removal of hydroxy protecting groups and cleavage of the linker were simultaneously performed via treatment with a 40% MeNH_2_ aqueous solution to afford glycosyl phosphate **4**. The crude mixture was analyzed by reverse-phase high-performance liquid chromatography (RP-HPLC). The profile is shown in Figure S1 (Supporting Information). Although it was indicated that the disaccharide 1-phosphate **4** was obtained as a main product, we detected the formation of the byproduct **6**. Additionally, the crude HPLC profile indicated that the disaccharide 1-phosphate **4** was formed as an α/β mixture, with two peaks having similar retention times, NMR analysis suggested that the former eluted peak was the α-isomer (isolated yield of 71%; the chemical shift of Man-*H*-1 was δ 5.43 ppm, in good agreement with α disaccharide 1-phosphate derivatives^24^), while the latter was the β-isomer (isolated yield of 21%; the chemical shift of Man-*H*-1 was δ 5.11 ppm). In addition, they had similar *m*/*z* values (calcd for [M – H]^−^, 601.1539; the former was 601.1530, and the latter was 601.1528).

**Scheme 2:**
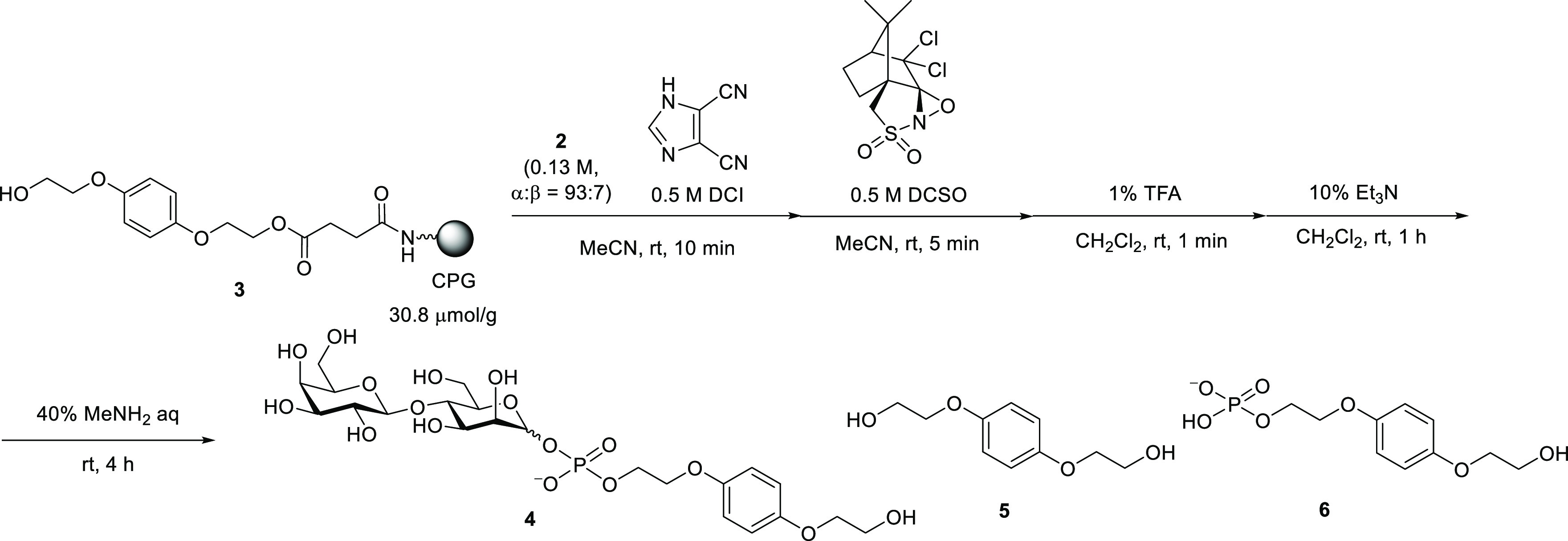
Solid-Phase Synthesis of Disaccharide 1-Phosphophate

The formation of **6** could be attributed to eliminating the phosphate moiety from the anomeric position, probably during the detritylation reaction. Thus, it was suggested that introducing an *o*-chlorobenzoyl protecting group could not prevent the formation of an oxocarbenium ion under acidic conditions. Subsequently, we attempted to synthesize a glycosyl boranophosphate using a similar procedure as that for glycosyl phosphate except for the oxidation step, which was replaced by a boronation step, and the removal of the cyanoethyl group was conducted by DBU treatment (Scheme S2). The crude RP-HPLC profile indicated the predominant formation of the disaccharide 1-boranophosphate, indicating that the glycosyl boranophosphate was tolerant of the detritylation conditions (Figure S2). Furthermore, treatment with a DCSO solution in MeCN resulted in an almost quantitative conversion of the glycosyl boranophosphodiester to its phosphate counterpart. We serendipitously found the reaction. Actually, we investigated the conversion of a boranophosphodiester to a phosphodiester via an acyl phosphite intermediate. As a control experiment, we examined the reaction between a boranophosphodiester and DCSO and found that the boranophosphate was converted to the phosphodiester. The mechanism and the scope of the reaction is under investigation. The procedure in Scheme 3 gave the glycosyl phosphate **4** as a major product (Table 1, entry 1, Figure S3). This result indicated that the degradation of the reaction intermediates was significantly suppressed via boranophosphate intermediates. In our previous reports, a boranophosphodiester linkage was converted to a phosphodiester linkage by multiple steps.^20−22^ In contrast, treatment with DCSO afforded a phosphodiester derivative in a single step, significantly facilitating the synthesis.

**Scheme 3:**
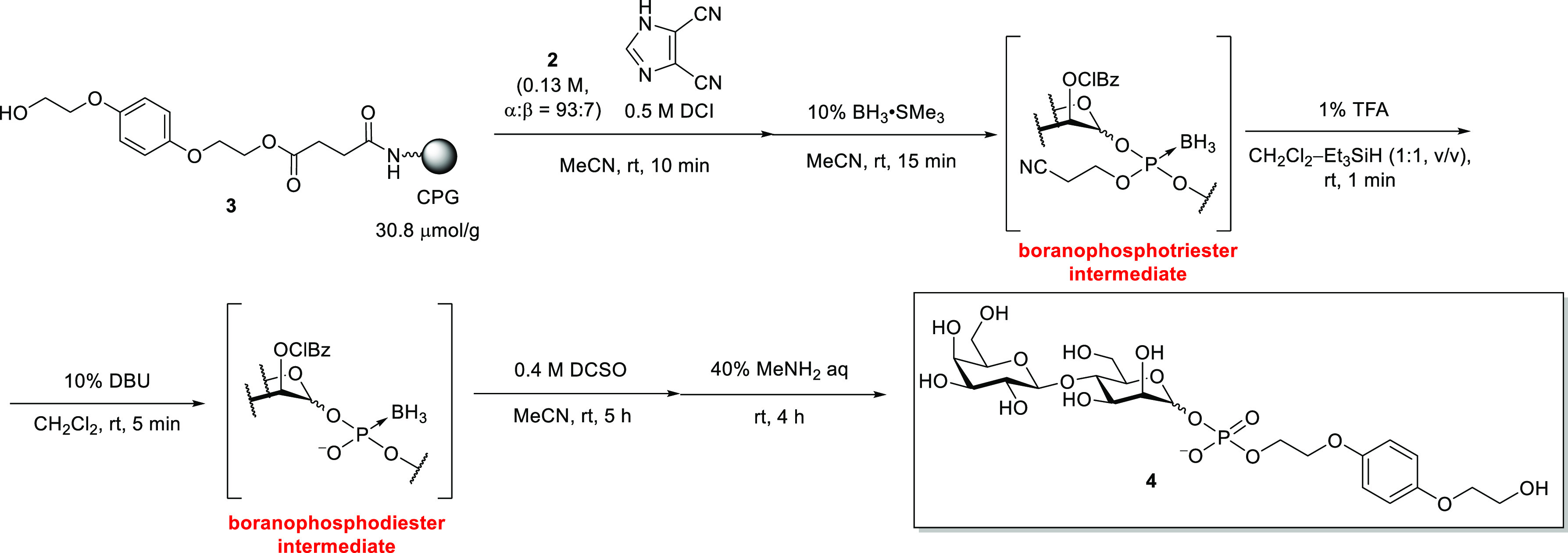
Solid-Phase Synthesis of Disaccharide 1-Phosphophate via a Boranophosphate Intermediate

**Table 1:** Investigation of Preactivation Conditions

entry	pre-activation	condensation time (min)	HPLC yield (%)^a^	α/β ratio^a^
1		10	97	82:18
2	+	10	77	97:3
3	+	60	90	97:3

a Determined by RP-HPLC.

As for the low diastereoselectivity of the condensation reaction, a similar phenomenon was also observed in our precedent study, which used an α/β mixture of a glycosyl phosphoramidite as a monomer.^17^ Since the β-isomer of the phosphoramidite seemed to be more reactive than the α-counterpart, a preactivation strategy was effective to suppress the formation of β-glycosyl phosphate linkages. In the preactivation strategy, the α/β mixture of glycosyl phosphoramidite was treated with an alcohol to consume the reactive β-isomer before a condensation reaction on a solid support.^17^ Thus, this strategy was applied to the synthesis (Scheme 4, Table 1). The monomer **2** was treated with an activator and an alcohol in MeCN solvent in a round-bottom flask to consume the β-isomer, and after 3 min the mixture was added to the reactor for the manual solid-phase synthesis by a syringe. It was found that 3-phenyl-1-propanol effectively prevented the formation of the β-isomer. However, the condensation efficiency was not satisfactory with a 10 min reaction time (Table 1, entry 2, Figure S4). Extending the condensation reaction time to 1 h improved the HPLC yield (Table 1, entry 3, Figure S4). Thus, the condensation reactions were conducted for 1 h in the following experiments.

**Scheme 4:**
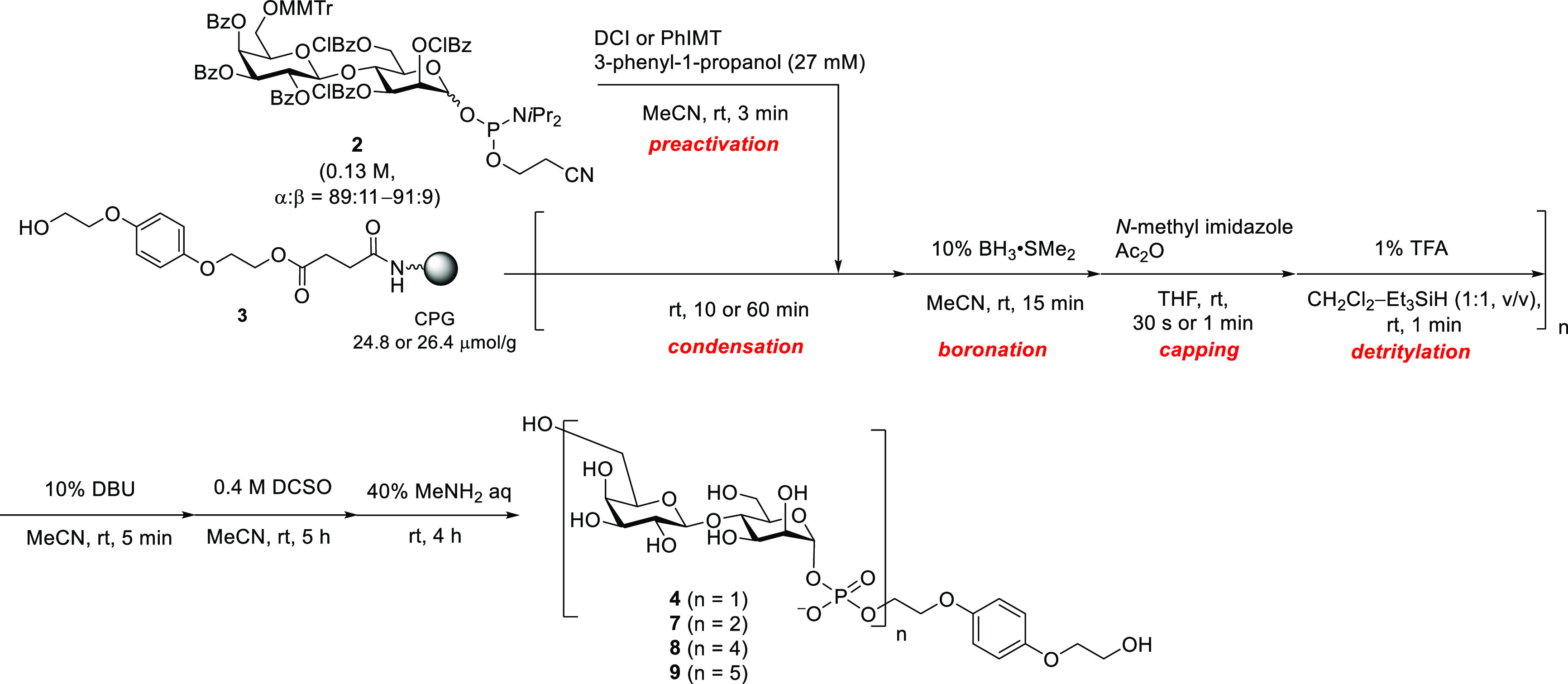
Solid-Phase Synthesis of Glycosyl Phosphate Repeating Units (*n* = 1, 2, 4, 5) Capping steps were conducted except for after the final condensation step.

We proceeded to synthesize glycosyl phosphates with multiple phosphate moieties. The synthesis of tetrasaccharide having two phosphate linkages was attempted following Scheme 4. A capping step was added after the condensation reaction using acetic anhydride. The coupling yields were estimated from RP-HPLC profiles of the reaction mixtures. The results are shown in Table 2. The second coupling efficiency was low compared with the first step when the condensation reactions were performed in the presence of DCI, regardless of DCI concentration (Table 2, entries 1 and 2, Figure S5). In contrast, when *N*-phenylimidazolium triflate (PhIMT) was used, high coupling efficiencies were achieved, and tetrarasaccharide was obtained with a 59% isolated yield (Table 2, entry 3, Figure S5). We proceeded to synthesize octasaccharide and decasaccharide, which contained four and five repeating units, respectively, by repeating the cycles of condensation in the presence of PhIMT, boronation, and detritylation, followed by the removal of cyanoethyl groups at boranophosphate linkages, oxidation with DCSO, and deprotection of the hydroxy groups and linker cleavage. It is worth noting that the solid support was washed with MeOH following the boronation steps to remove the boronation reagent and/or its residue, which would interfere with the next condensation reaction.^25^ The HPLC profiles of the reaction mixtures shown in Figures 2 and S6 indicates the predominant formation of the desired products. The octasaccharide and decasaccharide were isolated in 46% and 36% yields, respectively (Table 2, entries 4 and 5, Figures 2 and S6). However, in the ^1^H NMR spectrum of the isolated decasaccharide derivative, a signal around 5.2 ppm was observed (SI page S61), indicating the presence of small amounts of the β-isomer. The improvement in stereoselectivity still needs to be solved.

**Figure 2:**
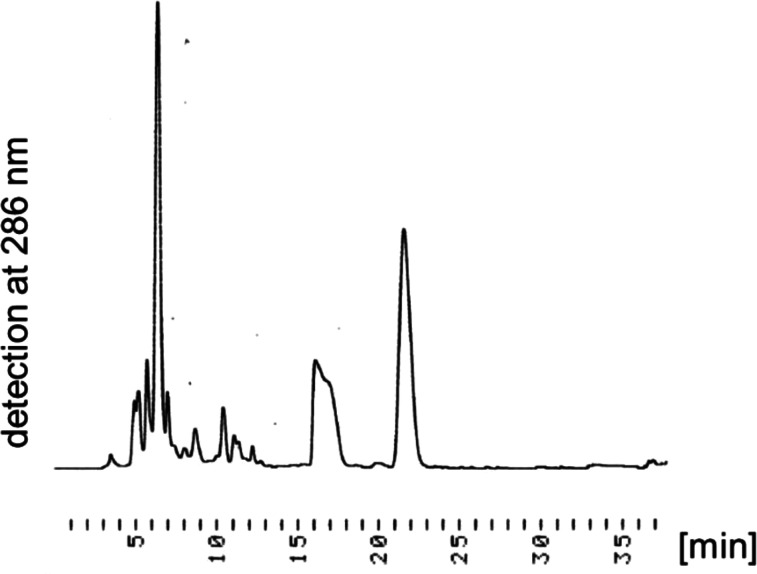
RP-HPLC profiles of the crude mixture of **9** (ODS, H_2_O (containing 0.1 M 1,1,1,3,3,3-hexafluoro-2-propanol and 8 mM Et_3_N)/MeOH = 95:5–77:23 over 30 min with a flow rate of 0.5 mL/min, detection at 286 nm and 60 °C, and *t*_R_ = 6.4 min (compound **9**).

**Table 2:** Synthesis of Oligo(glycosyl phosphate)

			coupling efficiency^a^ (%)		
entry	*n*	activator (concentration)	*n* = 1	*n* = 2	isolated yield (%)
1	2	DCI (0.5 M)	95	75	
2	2	DCI (1.0 M)	91	82	
3	2	PhIMT (1.0 M)	95	92	59
4^b^	4	PhIMT (1.0 M)			46
5^b^	5	PhIMT (1.0 M)			36

a Determined by RP-HPLC.

b Washing steps with MeOH were conducted after each condensation step.

In conclusion, we successfully synthesized glycosyl phosphate repeating units found in LPGs of *Leishmania* via solid-phase synthesis using boranophosphates as key reaction intermediates. Using glycosyl boranophosphates provides stability under acidic conditions, enabling chain elongation without notable side reactions. The conversion of boranophosphodiester into phosphodiester was almost quantitative using the DCSO treatment. The repeating units up to decasaccharide were successfully obtained following this strategy. Notably, this is the first report of synthesizing a homogeneous oligo-(glycosyl phosphate) structure bearing more than four phosphate groups without chemical modifications on pyranose moieties. Our method would significantly facilitate the synthesis and property evaluation of biomolecules bearing glycosyl phosphate moieties.

## Data Availability

The data underlying this study are available in the published article and its online Supporting Information.

## Supporting Information Available

Experimental procedures; HPLC profiles; and copies of ^1^H, ^13^C, ^31^P NMR, COSY, HMQC, HSQC, and HMBC spectra of new compounds (PDF)
