# Supplementary material for: Solid-Phase Synthesis of Glycosyl Phosphate Repeating Units via Glycosyl Boranophosphates as Stable Intermediates
Source: Org Lett. 2023 May 19;25(21):3927–31. doi: 10.1021/acs.orglett.3c01293 (PMC10243113; doi:10.1021/acs.orglett.3c01293)

Supporting Information

Solid-Phase Synthesis of Glycosyl Phosphate Repeating Units via Glycosyl Boranophosphates as Stable Intermediates

Kazuki Sato,[†,] Kazumasa Muramoto,[†] Tomoya Hagio,[†] Rintaro Iwata Hara,[†,‡] Takeshi Wada[†,]**

[†]Department of Medicinal and Life Sciences, Faculty of Pharmaceutical Sciences, Tokyo University of Science, 2641 Yamazaki, Noda, Chiba 278-8510, Japan

[‡]Department of Neurology and Neurological Science, Graduate School of Medicinal and Dental Sciences, Tokyo Medical and Dental University, 1-5-45 Yushima, Bunkyo-ku, Tokyo 113-8519, Japan

E-mail: kazuki_sato@rs.tus.ac.jp; twada@rs.tus.ac.jp

Table of Contents

1. General information **S3**

2. Experimental section **S4**

3. RP-HPLC profiles of crude disaccharide 1-phosphate and boranophosphate **S13**

4. RP-HPLC profiles of crude tetrasaccharide 1-phosphate **S17**

5. RP-HPLC profiles of crude octasaccharide and decasaccharide 1-phosphate **S19**

6. ^{1}H, ^{13}C, ^{31}P NMR, COSY, HMQC, HSQC, HMBC NMR spectra **S20**

1. General information

All the reactions were conducted under Ar atmosphere. Dry organic solvents were prepared by the appropriate relevant procedures. The ^{1}H NMR spectra were recorded at 400 or 500 MHz with tetramethylsilane (δ 0.0 ppm) as the internal standard in $CDCl_3$ or at 500 MHz or 600 MHz with MeCN (δ 2.06 ppm) as an internal standard in D_2O. The ^{13}C NMR spectra were recorded at 101 or 126 MHz in $CDCl_3$, which were used as the internal standards at δ 77.0 ppm. COSY, HMQC, HSQC, and HMBC were recorded on a 400 or 500 MHz spectrometer. The ^{31}P NMR spectra were recorded at 162 MHz or 202 MHz with H_3PO_4 (δ 0.0) as the external standard in $CDCl_3$. Structural assignments were made with additional information from COSY, HMQC, HSQC, and HMBC experiments. Hydrogen multiplicity information was obtained by DEPT spectra. IR spectra were obtained using an ATR-IR spectrometer. Optical rotations were measured on a polarimeter and given in units of (deg·mL)/(g·dm). Analytical TLC was performed on commercial glass plated 0.25 mm thickness silica gel layer. Manual silica gel column chromatography was performed using spherical, neutral, 63–210 µm silica gel unless otherwise noted. Automated silica gel column chromatography was performed on amino silica gel (Yamazen UNIVERSAL Premium column (30 µm)) (Yamazen Corporation) using automated flash chromatography system W-prep 2XY (Yamazen Corporation). The solid-phase synthesis was carried out manually using a glass filter (10 × 50 mm) with a stopper at the top and a stopcock at the bottom as a reaction vessel, and compounds synthesized by this method were analyzed and purified by RP-HPLC and identified by ESI MS. The detections in RP-HPLC were achieved at 286 nm at a temperature of 30 °C and a flow rate of 0.5 mL/min using a C18 column (100 Å, 3.9 × 150 mm) unless otherwise noted. The isolated yields of the synthesized oligomers were estimated by UV-Vis spectroscopy using a molar absorption constant of the spacer at 286 nm ($\varepsilon = 2.22 \times 10^3$ L mol^{-1} cm^{-1})[1]. The quantities of the products obtained by solid-phase synthesis were too small to acquire ^{13}C NMR spectra.

2. Experimental section

Scheme S1. Synthesis of phosphoramidite monomer **2**

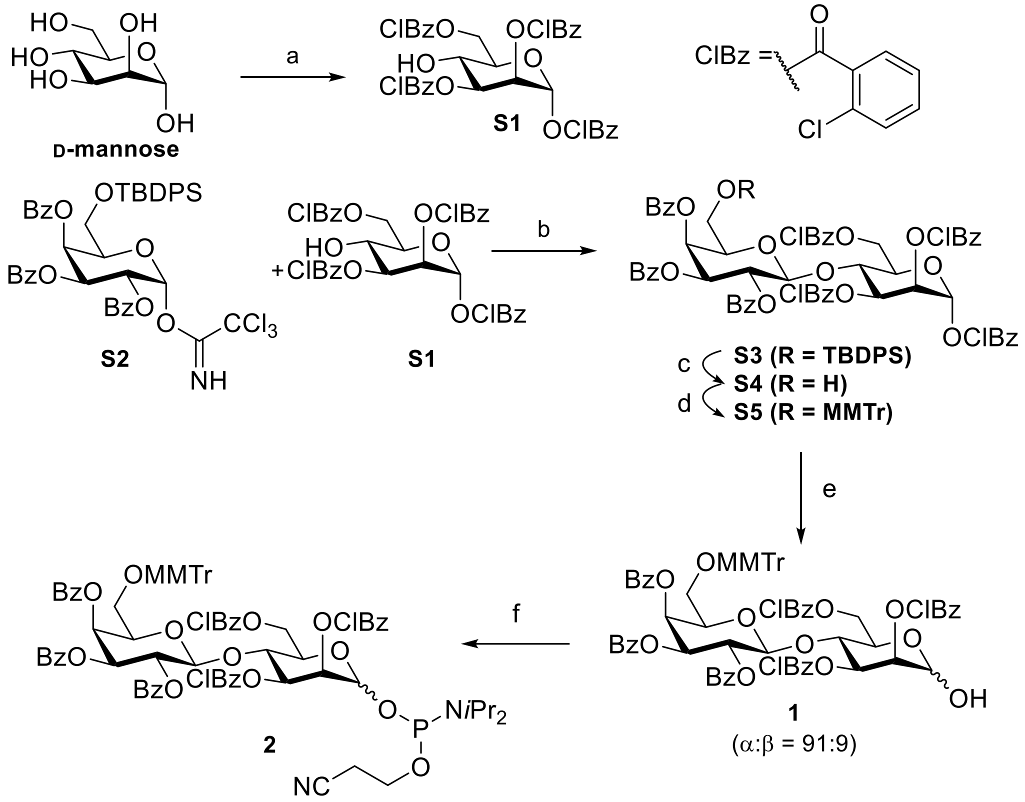

a) *o*-Chlorobenzoyl chloride (3.8 equiv), pyridine, −40 °C, 2 h, 37%; b) TMSOTf (0.3 equiv), CH_2Cl_2, 0 °C, 1 h; c) TBAF•$3H_2O$ (3.0 equiv), AcOH (3.0 equiv), THF, 0 °C to rt, 4 h, 59% over 2 steps; (d) MMTrCl (3.0 equiv), pyridine, rt, 20 h, 97%; (e) $MeNH_2$ (11 equiv), THF–MeOH (15:1, v/v), −30 °C, 2 h, 93%; (f) 2-cyanoethyl *N*,*N*-diisopropylchlorophosphoramidite (1.5 equiv), *i*Pr_2NEt (3.0 equiv), 1,4-dioxane–CH_2Cl_2 (9:1, v/v), rt, 14 h, 72%.

1, 2, 3, 6-*O*-Tetra (*o*-chlorobenzoyl)-α-D-mannopyranose (S1)

MS4A, D-mannose (0.90 g, 5.0 mmol), and dry pyridine (40 mL) were successively added to a reaction vessel and then, *o*-chlorobenzoyl chloride (2.4 mL, 19 mmol) in dry pyridine (9.6 mL) was added dropwise over 10 min at –40 °C to the mixture while stirring using an ethanol bath. The mixture was further stirred for 2 h, and the reaction was quenched by addition of MeOH (10 mL). The mixture was then diluted with $CHCl_3$ (100 mL) and washed with saturated $NaHCO_3$ aqueous solutions (3×100 mL). The organic layer was dried over Na_2SO_4, filterer, and concentrated under reduced pressure. The residue was purified by automated silica gel column chromatography (amino silica, 40 g, L size) using a linear gradient of EtOAc–hexane (50:50–90:10, v/v) as an eluent. The fractions containing compound **S1** were collected. On the other hand, the fraction containing compound **S1** and byproduct(s) were collected and concentrated under reduced pressure. The residue was purified by automated silica gel column chromatography (amino silica, 16 g, M size) using a linear gradient of EtOAc–hexane (33:67–90:10, v/v) as eluent. The fractions containing compound **S1** were collected, and the combined fractions were concentrated under reduced pressure to afford compound **S1** as a colorless foam (1.37 g, 1.87 mmol, 37% yield).

$[\alpha]_D^{20}$ +19.8 (*c* 0.70, $CHCl_3$); IR (neat, cm^{-1}) 1718, 1592, 1437, 1276, 1239, 1102, 1034, 950, 739, 682; ^1H NMR (500 MHz, $CDCl_3$) δ 7.99–7.96 (m, 1H, Ar), 7.86 (td, *J* = 1.0, 2.7 Hz, 1H, Ar), 7.85–7.84 (m, 1H, Ar), 7.82 (ddd, *J* = 0.5, 1.5, 7.8 Hz, 1H, Ar), 7.54–7.49 (m, 2H, Ar), 7.45–7.38 (m, 7H, Ar), 7.29–

7.26 (m, 1H, Ar), 7.22–7.17 (m, 2H, Ar), 6.58 (d, *J* = 1.9 Hz, 1H, H-1), 5.87 (dd, *J* = 2.0, 3.3 Hz, 1H, H-2), 5.78 (dd, *J* = 3.3, 9.9 Hz, 1H, H-3), 4.85 (dd, *J* = 3.9, 12.3 Hz, H-6), 4.63 (dd, *J* = 4.6, 12.2 Hz, 1H, H-6′), 4.42 (dt, *J* = 3.6, 9.9 Hz, 1H, H-4), 4.34 (ddd, *J* = 2.3, 3.6, 10.0 Hz, 1H, H-5), 2.96 (d, *J* = 4.1 Hz, -OH);
^{13}C NMR (126 MHz) δ 165.9, 165.3, 164.1, 163.1 (C=O), 134.2, 134.2, 134.0, 133.8 (C(4), Ar), 133.6, 133.2, 133.0, 132.8, 132.2, 131.9, 131.9, 131.8, 131.5, 131.2, 131.1, 131.0 (CH, Ar), 129.4, 129.1, 128.7, 128.4 (C(4), Ar), 126.9, 126.7, 126.6, 126.6 (CH, Ar), 91.7 ($^1J_{C\text{-}H}$ = 178 Hz, C-1), 73.5 (C-5), 72.6 (C-3), 69.5 (C-2), 65.4 (C-4), 63.6 (C-6);
HRMS (ESI-QTOF) *m/z*: calcd for $[M + NH_4]^+$ $C_{34}H_{28}Cl_4NO_{10}^+$, 750.0462, found 750.0462.

***o*-Chlorobenzoyl-*O*-(2,3,4-tri-*O*-benzoyl-β-D-galactopyranosyl)-(1→4)-*O*-2,3,6-tri-*O*-(*o*-chlorobenzoyl)-α-D-mannopyranoside (S4)**

Compound **S1** (9.93 g, 13.5 mmol) and 2,3,4-tri-*O*-benzoyl-6-*O*-TBDPS-α-D-galactopyranosyl trichloroacetamidate **S2**[2] (15.4 g, 17.6 mmol) were dried by repeated co-evaporation by dry toluene and dissolved in dry CH_2Cl_2 (80 mL). TMSOTf (0.73 mL, 4.1 mmol) in dry CH_2Cl_2 (54.5 mL) was added dropwise over 10 min at 0 °C using an ice bath while stirring. After 1 h, the reaction was quenched with addition of a saturated $NaHCO_3$ aqueous solution (5 mL) and the mixture was washed with saturated $NaHCO_3$ aqueous solutions three times. The aqueous layers were combined and extracted with $CHCl_3$ once. The organic layers were combined, dried over Na_2SO_4, filterer, and concentrated under reduced pressure. The residue was roughly purified by manual silica gel column chromatography (neutral silica, 400 g) using EtOAc–hexane (1:3–1:1, v/v) as an eluent to yield crude compound **S3** (19.2 g). A part of crude mixture (18.9 g) and AcOH (2.2 mL, 39.3 mmol) were dissolved in dry THF (221 mL) and tetrabutylammonium fluoride·$3H_2O$ (12.4 g, 39.3 mmol) in dry THF (39 mL) was added dropwise at 0 °C using an ice bath. The mixture was stirred for 4 h at rt. To the mixture, a saturated $NaHCO_3$ aqueous solution (5 mL) was added, and the mixture was concentrated under reduced pressure. The residue was dissolved in toluene (100 mL) and washed with saturated $NaHCO_3$ aqueous solutions (3 × 100 mL). The aqueous layers were combined and extracted with toluene (100 mL). The organic layers were combined, dried over Na_2SO_4, filterer, and concentrated under reduced pressure. The residue was purified by manual silica gel column chromatography three times using EtOAc–hexane (1:2–1:1, for the first time, 3:4–1:1, for the second time, 1:2–4:3, for the third time, v/v) as an eluent to yield compound **S4** as a colorless foam (9.59 g, 7.9 mmol, 59% yield from compound **S1**).
$[\alpha]_D^{25}$ +81.9 (*c* 0.31, $CHCl_3$);
^{1}H NMR ($CDCl_3$, 400 MHz) δ 8.07–7.99 (m, 4H, Ar), 7.96–7.93 (m, 1H), 7.87 (dd, *J* = 1.4, 8.0 Hz, 1H, Ar), 7.85–7.75 (m, 4H, Ar), 7.67–7.62 (m, 1H, Ar), 7.53–7.31 (m, 13H, Ar), 7.27–7.20 (m, 6H, Ar), 6.88–6.83 (m, 1H, Ar), 6.51 (d, *J* = 2.3 Hz, 1H, Man-H-1), 6.00 (dd, *J* = 3.3, 9.3 Hz, 1H, Man-H-3), 5.86–5.81 (m, 2H, Man-H-2, Gal-H-2), 5.66 (d, *J* = 3.2 Hz, 1H, Gal-H-4), 5.43 (dd, *J* = 3.4, 10.3 Hz, 1H, Gal-H-3), 4.99 (d, *J* = 8.0 Hz, 1H, Gal-H-1), 4.72 (t, *J* = 9.6 Hz, 1H, Man-H-4), 4.60–4.51 (m, 2H, Man-H-6, Man-H-6′), 4.23 (td, *J* = 2.4, 9.6 Hz, 1H, Man-H-5), 3.79 (t, *J* = 6.9 Hz, 1H, Gal-H-5), 3.26–3.16 (m, 1H, Gal-H-6), 3.14–3.05 (m, 1H, Gal-H-6′), 2.5–2.3 (br, 1H, -OH);
^{13}C NMR ($CDCl_3$, 101 MHz) δ 166.7, 165.4, 165.1, 165.0, 164.2, 164.2, 163.0 (C=O), 134.3, 134.1, 134.0 (C(4), Ar), 133.9, 133.6, 133.5, 133.3, 133.3, 132.9, 132.3, 132.1, 131.9, 131.4, 131.3, 131.1, 130.1, 129.8, 129.7 (CH, Ar), 129.4, 129.0 (C(4), Ar), 128.7, 128.6, 128.3, 126.9, 126.7, 126.7, 126.3 (CH, Ar), 101.4 (Gal-C-1), 91.3 (Man-C-1), 74.1 (Gal-C-5), 73.4 (Man-C-4), 72.0, 72.0 (Gal-C-3, Man-C-5), 70.0, 69.9, 69.9 (Man-C-2, Man-C-3, Gal-C-2), 68.6 (Gal-C-4), 62.8 (Man-C-6), 60.0 (Gal-C-6);
HRMS (ESI-FT-ICR) *m/z*: calcd for $[M + NH_4]^+$ $C_{61}H_{50}Cl_4NO_{18}^+$, 1224.1777, found 1224.1750.

***o*-Chlorobenzoyl-*O*-(2,3,4-tri-*O*-benzoyl-6-*O*-(4-methoxytrityl)-β-D-galactopyranosyl)-(1→4)-*O*-2,3,6-tri-*O*-(*o*-chlorobenzoyl)-α-D-mannopyranoside (S5)**

Compound **S4** (9.54 g, 7.9 mmol) was dissolved in dry pyridine (79 mL). 4-Methoxytrityl chloride (MMTrCl, 7.31 g, 23.7 mmol) was added to the mixture, and the mixture was stirred at rt for 20 h. The reaction was quenched by the addition of MeOH (20 mL) and the mixture was concentrated under reduced pressure. The residue was dissolved in EtOAc (100 mL) and washed with saturated $NaHCO_3$ aqueous solutions (3 × 100 mL). The aqueous layers were combined and back-extracted with EtOAc (2 × 100 mL). The organic layers were combined, dried over Na_2SO_4, filterer, and concentrated under reduced pressure. The residue was purified by silica gel column chromatography (neutral silica, 400 g) using EtOAc–hexane (1:2–1:1, v/v) as eluent to yield compound **S5** as colorless foam (11.3 g, 7.6 mmol, 97%).

$[\alpha]_D^{23}$ +52.5 (*c* 0.51, $CHCl_3$); IR (neat, cm^{-1}) 2932, 1728, 1592, 1473, 1452, 1437, 1280, 1242, 1095, 1069, 1038, 957, 825, 745, 704, 649, 613;
^{1}H NMR ($CDCl_3$, 400 MHz) δ 7.98 (d, *J* = 7.3 Hz, 2H, Ar), 7.94–7.90 (m, 2H, Ar), 7.85–7.81 (m, 3H, Ar), 7.76 (d, *J* = 7.3 Hz, 3H, Ar), 7.56 (t, *J* = 7.6 Hz, 1H, Ar), 7.49–7.28 (m, 14H, Ar), 7.27–7.17 (m, 8H, Ar), 7.15–7.06 (m, 9H, Ar), 7.00 (dt, *J* = 1.1, 7.7 Hz, 1H, Ar), 6.60–6.55 (m, 2H, Ar), 6.47 (d, *J* = 1.4 Hz, 1H, Man-1-H), 6.34 (t, *J* = 7.6 Hz, 1H, Ar), 6.12 (d, *J* = 3.2 Hz, 1H, Gal-H-4), 5.86–5.81 (m, 2H, Man-H-2, Man-H-3), 5.69 (dd, *J* = 8.0, 10.3 Hz, 1H, Gal-H-2), 5.55 (dd, *J* = 3.2, 10.5 Hz, 1H, Gal-H-3), 4.94 (d, *J* = 7.8 Hz, 1H, Gal-H-1), 4.75 (t, *J* = 10.1 Hz, 1H, Man-H-4), 4.62–4.49 (m, 2H, Man-H-6, 6′), 4.17–4.12 (m, 1H, Man-H-5), 4.01 (dd, *J* = 5.0, 9.6 Hz, Gal-H-5), 3.62 (s, 3H, -OC<u>H</u>$_3$), 3.25 (dd, *J* = 4.8, 8.5 Hz, 1H, Gal-H-6), 3.11 (t, *J* = 9.2 Hz, 1H, Gal-H-6′);
^{13}C NMR ($CDCl_3$, 101 MHz) δ 165.4, 165.1, 165.1, 164.7, 164.4, 163.9, 162.9 (C=O), 158.3, 143.9, 143.4, 134.6, 134.3, 134.2, 133.7 (C(4), Ar), 133.5 (CH, Ar), 133.5 (C(4), Ar), 133.4, 133.1, 133.1, 132.9, 132.7, 132.3, 132.0, 131.8, 131.4, 131.0, 130.9, 130.2, 129.9, 129.7, 129.7 (CH, Ar), 129.4, 129.2, 129.1, 128.8, 128.7 (C(4), Ar), 128.5 (CH, Ar), 128.4 (C(4), Ar), 128.3, 128.2, 128.2, 128.1, 127.8, 127.7, 126.9, 126.7, 126.7, 126.6, 126.3, 113.0 (CH, Ar), 101.3 (Gal-C-1), 91.2 (Man-C-1), 86.4 (-<u>C</u>Ar$_3$), 72.5 (Gal-C-5), 72.4 (Man-C-4), 72.3 (Man-C-5), 72.0 (Gal-C-3), 70.1, 70.0, 69.8 (Man-C-2, Man-C-3, Gal-C-2), 67.5 (Gal-C-4), 62.7 (Man-C-6), 59.1 (Gal-C-6), 55.0 (-O<u>C</u>H$_3$);
HRMS (ESI-FT-ICR) *m/z*: calcd for $[M + NH_4]^+$ $C_{81}H_{62}Cl_4NaO_{19}^+$, 1501.2532, found 1501.2535.

(2,3,4-Tri-*O*-benzoyl-6-*O*-(4-methoxytrityl)-β-D-galactopyranosyl)-(1→4)-*O*-2,3,6-tri-*O*-(*o*-chlorobenzoyl)-D-mannopyranose (1, α, β mixture)

To a solution of compound **S5** (4.65 g, 3.1 mmol) in dry THF (16 mL), a $MeNH_2$ (1.4 mL, 34 mmol) solution in THF–MeOH (17.5 mL, 88:12, v/v) was added dropwise over 5 min at −30 °C using a methanol bath. The mixture was stirred for 2 h at −30 °C using a methanol bath, diluted with cold toluene (40 mL), and then concentrated under reduced pressure at 0 °C using an ice bath. The residue was purified by silica gel column chromatography (amino silica, 200 g) using EtOAc–hexane (11:7–4:1, v/v) as eluent to yield compound **1** as a colorless foam (3.93 g, 2.93 mmol, 93%, α:β = 91:9).
$[\alpha]_D^{23}$ = +37.8 (*c* 0.45, $CHCl_3$); IR (neat, cm^{-1}) 3463, 1727, 1593, 1510, 1492, 1475, 1451, 1437, 1281, 1244, 1090, 1069, 1040, 904, 833, 745, 704, 666, 650;
^{1}H NMR ($CDCl_3$, 500 MHz, for β isomer, only representative signals are listed) δ 7.98–7.95 (m, 2H, Ar), 7.88 (dd, *J* = 1.7, 7.9 Hz, 1H, Ar), 7.84–7.78 (m, 3H, Ar), 7.76–7.70 (m, 3H, Ar), 7.58–7.54 (m, 1H), 7.45–7.31 (m, 9H, Ar), 7.30–7.03 (m, 18H, Ar), 6.99 (dt, *J* = 1.7, 7.6 Hz, 1H, Ar), 6.61–6.56 (m, 2H, Ar), 6.41 (dt, *J* = 1.1, 7.6 Hz, β-1H, Ar), 6.35 (dt, *J* = 1.0, 7.6 Hz, 1H, Ar), 6.08–6.06 (m, 1H, Gal-H-4), 5.82 (dd, *J* = 0.9, 3.1 Hz, β-1H, Man-H-2), 5.78 (dd, *J* = 3.2, 9.9 Hz, 1H, Man-H-3), 5.68–5.64 (m, 2H, Man-H-2, Gal-H-2), 5.63 (dd, *J* = 7.9, 10.3 Hz, β-1H, Gal-H-3), 5.52 (dd, *J* = 3.4, 10.3 Hz, Gal-H-3), 5.44 (dd, *J* = 3.1, 9.8 Hz, β-1H, Man-H-3), 5.32 (brs, 1H, Man-H-1), 5.06 (d, *J* = 8.2 Hz, β-1H, Man-H-1), 4.92 (d, *J* = 8.0 Hz, 1H, Gal-H-1), 4.88 (d, *J* = 7.9 Hz, β-1H, Gal-H-1), 4.64 (t, *J* = 9.9 Hz, 1H, Man-H-4), 4.60–4.51 (m, 2H, Man-H-6, Man-H-6′), 4.21–4.17 (m, 1H, Man-H-5), 3.95 (dd, *J* = 5.5, 9.1 Hz, 1H, Gal-H-

5), 3.61 (s, 3H, -OCH_3), 3.53–3.48 (m, β-1H, OH), 3.26 (dd, *J* = 5.0, 8.7 Hz, 1H, Gal-H-6), 3.17 (brs, 1H, -OH), 3.12 (t, *J* = 9.0 Hz, 1H, Gal-H-6′);
^{13}C NMR ($CDCl_3$, 126 MHz, for β isomer, only representative signals are listed) δ 165.5, 165.4, 165.2, 164.7, 164.6, 164.0 (C=O), 158.4, 144.0, 143.5, 134.7, 134.2, 133.8, 133.5 (C(4), Ar), 133.3, 133.2, 133.1, 132.9, 132.8, 132.5, 132.2, 132.0, 131.8, 131.1, 131.0, 130.8, 130.3, 129.9, 129.8, 129.8 (CH, Ar), 129.6, 129.4, 129.3, 128.9, 128.8, 128.8 (C(4), Ar), 128.5, 128.4, 128.3, 128.2, 128.1, 127.8, 127.7, 126.8, 126.7, 126.5, 126.3, 113.1 (CH, Ar), 101.1 (Gal-C-1), 101.1 ((β-Gal-C-1), 92.9 (β-Man-C-1, $^1J_{CH}$ = 163 Hz), 92.0 (Man-C-1, $^1J_{CH}$ = 173 Hz), 86.5 (-CAr_3), 72.9 (Man-C-4), 72.5 (Gal-C-5), 72.3 (β-Man-C-3), 72.1 (Gal-C-3), 71.8 (β-Man-C-3), 71.8 (β-Man-C-2), 71.7 (Gal-C-2), 70.1 (Man-C-2), 69.8, 69.5 (Man-C-3, Gal-C-5), 67.5 (Gal-C-4), 63.1 (Gal-C-6), 59.3 (Man-C-6);
HRMS (ESI-QTOF) *m/z*: calcd for $[M + NH_4^+]^+$ $C_{74}H_{63}Cl_3NO_{18}^+$, 1358.3105, found 1358.3104.

2-Cyanoethyl (2,3,4-Tri-*O*-benzoyl-6-*O*-(4-methoxytrityl)-β-D-galactopyranosyl)-(1→4)-*O*-2,3,6-tri-*O*-(*o*-chlorobenzoyl)-D-mannopyranosyl *N, N*-diisopropyl phosphoramidite (2, α, β mixture)

Compound **1** (5.28 g, 3.9 mmol) was dried by repeated co-evaporation with dry 1,4-dioxane and dissolved in dry 1,4-dioxane–CH_2Cl_2 (39 mL, 9:1, v/v). DIPEA (2 mL, 11.5 mmol), and 2-cyanoethyl *N,N*-diisopropylchlorophosphoramidite (1.3 mL, 5.9 mmol) were successively added to the mixture at rt. The mixture was stirred for 14 h and the reaction was quenched by the addition of H_2O (1 mL). The mixture was diluted with EtOAc (50 mL) and washed with saturated $NaHCO_3$ aqueous solutions (3×80 mL). The aqueous layers were combined and extracted with EtOAc (50 mL). The organic layers were combined, dried over Na_2SO_4, filterer, and concentrated under reduced pressure. The residue was purified by silica gel column chromatography (amino silica, 80 g) using toluene–hexane (97:3, v/v) as eluent to yield compound **2** as a colorless foam (4.40 g, 2.8 mmol, 72%, α:β = 89:11).
$[\alpha]_D^{23}$ +42.7 (*c* 0.50, $CHCl_3$); IR (neat, cm^{-1}) 2978, 2934, 1729, 1593, 1510, 1451, 1437, 1280, 1246, 1107, 1092, 1069, 1026, 974, 831, 746, 704, 643;
^{1}H NMR (500 MHz, $CDCl_3$, for β-isomer, only representative signals are listed) δ 8.02–7.98 (m, α-2H, Ar), 7.97–7.93 (m, β-2H, Ar), 7.90 (dd, *J* = 1.7, 7.9 Hz, β-4H, Ar), 7.87–7.69 (m, α-7H, β-3H, Ar), 7.60–7.52 (m, α-1H, β-1H, Ar), 7.48–7.29 (m, α-10H, β-10H, Ar), 7.25–7.16 (m, α-8H, β-8H, Ar), 7.15–7.04 (m, α-9H, β-9H, Ar), 7.02–6.95 (m, α-1H, β-1H, Ar), 6.60–6.56 (m, α-2H, β-2H, Ar), 6.45–6.40 (m, β-1H, Ar), 6.37 (dt, *J* = 1.0, 8.2 Hz, α-0.5H, Ar), 6.33 (dt, *J* = 0.9, 7.9 Hz, α-0.5H, Ar), 6.11 (dd, *J* = 0.5, 3.3 Hz, α-0.5H, Gal-H-4), 6.09 (dd, *J* = 0.5, 3.3 Hz, α-0.5H, Gal-H-4), 6.08–6.06 (m, β-1H, Gal-H-4), 5.73 (dd, *J* = 3.2, 9.8 Hz, α-0.5H, Man-H-3), 5.70–5.64 (m, α-1.5H, Man-H-3, Gal-H-2), 5.64–5.61 (m, α-0.5H, Man-H-2), 5.56–5.50 (m, α-1.5H, Man-H-2, Gal-H-3), 5.37 (dd, *J* = 1.9, 8.7 Hz, α-0.5H, Man-H-1), 5.36 (dd, *J* = 1.9, 8.7 Hz, α-0.5H, Man-H-1), 4.90 (d, *J* = 8.0 Hz, α-0.5H, Gal-H-1), 4.89 (d, *J* = 8.0 Hz, α-0.5H, Gal-H-1), 4.88 (d, *J* = 7.9 Hz, β-0.5H, Gal-H-1), 4.85 (d, *J* = 7.9 Hz, β-0.5H, Gal-H-1), 4.71 (dd, *J* = 1.9, 12.1 Hz, β-1H, Gal-H-3), 4.65 (t, *J* = 9.9 Hz, α-0.5H, Man-H-4), 4.62–4.55 (m, α-1.5H, Man-H-4, Man-H-6), 4.53–4.43 (m, α-1H, Man-H-6′), 4.11–4.07 (m, α-0.5H, Man-H-5), 4.03–3.82 (m, α-2H, Man-H-5, Gal-H-5, -OCH_2CH_2CN), 3.79–3.50 (m, α-5.5H, -OCH_2CH_2CN, -$NCH(CH_3)_2$, -OCH_3), 3.24 (dd, *J* = 5.0, 8.5 Hz, α-0.5H, Gal-H-6), 3.18 (dd, *J* = 4.9, 8.6 Hz, α-0.5H, Gal-H-6), 3.14–3.04 (m, α-1H, Gal-H-6′), 2.70–2.67 (m, α-1H, -OCH_2CH_2CN), 2.41 (dt, *J* = 3.0, 6.6 Hz, α-1H, -OCH_2CH_2CN), 1.24 (d, *J* = 6.8 Hz, α-3H, -$NCH(CH_3)_2$), 1.19 (d, *J* = 6.8 Hz, α-3H, -$NCH(CH_3)_2$), 1.16 (d, *J* = 6.8 Hz, α-3H, -$NCH(CH_3)_2$), 1.02 (d, *J* = 6.8 Hz, α-3H, -$NCH(CH_3)_2$);
^{13}C NMR for α-isomer (126 MHz, $CDCl_3$) δ 165.4, 165.3, 165.3, 165.1, 165.1, 164.8, 164.6, 164.5, 164.0, 163.9 (C=O), 158.3, 143.9, 143.9, 143.4, 134.6, 134.6, 134.1, 134.0, 133.8, 133.8, 133.5, 133.5 (C(4), Ar), 133.4, 133.3, 133.1, 133.1, 132.9, 132.9, 132.8, 132.7, 132.5, 132.4, 132.3, 132.1, 131.9, 131.9, 131.8, 131.0, 131.0, 130.8, 130.7, 130.3, 130.3, 130.0, 129.8, 129.7, 129.7 (CH, Ar), 129.7, 129.6, 129.5, 129.4, 129.4, 129.3, 129.3, 129.0, 128.9, 128.9, 128.8, 128.8 (C(4), Ar), 128.5, 128.5, 128.4, 128.3, 128.2, 128.1, 128.1, 127.8, 127.7, 126.7, 126.5, 126.2, 126.2 (CH, Ar), 117.5 (-OCH_2CH_2CN, one of diastereomers),

117.1 (-$OCH_2CH_2\underline{C}N$, one of diastereomers), 113.0, 113.0 (CH, Ar), 101.4 (Gal-C-1, one of diastereomers), 101.2 (Gal-C-1, one of diastereomers), 92.8 (d, $^2J_{C\text{-}P}$ = 19.7 Hz, $^1J_{C\text{-}H}$ = 172 Hz, Man-C-1, one of diastereomers), 92.3 (d, $^2J_{C\text{-}P}$ = 20.6 Hz, $^1J_{C\text{-}H}$ = 172 Hz, Man-C-1, one of diastereomers), 86.4 (-$\underline{C}(Ar)_3$), 73.1 (Man-C-4, one of diastereomers), 72.5 (Man-C-4, one of diastereomers), 72.1 (Gal-C-5, one of diastereomers), 72.1 (Gal-C-5, one of diastereomers), 72.0 (Man-C-2, one of diastereomers), 71.8 (d, $^3J_{C\text{-}P}$ = 3.6 Hz, Man-C-2, one of diastereomers), 70.5 (Man-C-5, one of diastereomers), 70.4 (Man-C-5, one of diastereomers), 70.2 (Gal-C-2, one of diastereomers), 70.1 (Gal-C-2, one of diastereomers), 70.0 (Man-C-3, one of diastereomers), 69.8 (Man-C-3, one of diastereomers), 67.6 (Gal-C-4), 63.1 (Man-C-6, one of diastereomers), 63.0 (Man-C-6, one of diastereomers), 59.2 (Gal-C-6, one of diastereomers), 59.1 (Gal-C-6, one of diastereomers), 58.9 (d, $^2J_{C\text{-}P}$ = 20.0 Hz, -P-O-$\underline{C}H_2CH_2CN$, one of diastereomers), 58.8 (d, $^2J_{C\text{-}P}$ = 20.2 Hz, -P-O-$\underline{C}H_2CH_2CN$, one of diastereomers), 55.0 (-$O\underline{C}H_3$), 43.8, 43.7, 43.7, 43.6 (-$N\underline{C}H(CH_3)_2$), 24.6, 24.6, 24.5, 24.5, 24.3, 24.2 (-$NCH(\underline{C}H_3)_2$), 20.3 (d, $^3J_{C\text{-}P}$ = 7.2 Hz, -$OCH_2\underline{C}H_2CN$, one of diastereomers), 20.0 (d, $^3J_{C\text{-}P}$ = 7.9 Hz, -$OCH_2\underline{C}H_2CN$, one of diastereomers);
representative signals for β-isomer: δ 93.6 (d, $^2J_{C\text{-}P}$ = 19.2 Hz, Man-C-1, one of diastereomers), 93.3 (d, $^2J_{C\text{-}P}$ = 25.2 Hz, Man-C-1, one of diastereomers);
^{31}P NMR ($CDCl_3$, 202 MHz) for α-isomer: δ 151.7, 150.2, for β-isomer: δ 153.8, 151.1;
HRMS (ESI-QTOF) *m/z*: calcd for $[M + NH_4]^+$ $C_{83}H_{80}Cl_3N_3O_{19}P^+$, 1558.4184, found; 1558.4183.

Solid-phase synthesis of disaccharide 1-phosphate (Scheme 2)

The CPG-loaded *O*-MMTr protected hydroquinone spacer (30.8 μmol/g, 0.50 μmol), via a succinyl linker (**3**), was treated in a reaction vessel with 1% TFA in dry CH_2Cl_2 (4 × 15 s, 1 mL each) and washed with dry CH_2Cl_2 (4 × 1 mL) and MeCN (3 × 1 mL). Thereafter, it was dried in vacuo for 10 min. Then, the disaccharide 1-phosphoramidite monomer (**2**, α:β = 93:7, 30.9 mg, 20 μmol), which was dried *in vacuo* overnight, was added to the reaction vessel and dried *in vacuo* for 3 min. A 0.5 M solution of 4,5-dicyanoimidazole (DCI, 8.9 mg, 75 μmol) in dry MeCN (150 μL), which was dried over MS 3Å overnight, was added under Ar atmosphere to the reaction vessel. After 10 min, the CPG was washed with dry MeCN (3 × 1 mL) and dried in vacuo for 5 min. The resultant phosphite was oxidized upon treatment with a 0.5 M solution of (+)-(8,8-dichlorocamphorylsulfonyl)oxaziridine (DCSO) (22.4 mg, 75 μmol) in dry MeCN (150 μL) and the reaction vessel was left for 5 min. Then, the CPG was washed with dry MeCN (3 × 1 mL) and dry CH_2Cl_2 (4 × 1 mL) and the detritylation reaction was carried out using 1% TFA in dry CH_2Cl_2 (4 × 15 s, 1 mL each). Subsequently, a 10% Et_3N solution in CH_2Cl_2 (1 mL, v/v) was added to the reaction vessel. After 1 h, the CPG was washed with CH_2Cl_2 (4 × 1 mL). The CPG was then treated with a 40% $MeNH_2$ aqueous solution (4 mL) at rt for 4 h, filtered, and washed with EtOH. The filtrate and the washings were combined, concentrated under reduced pressure, and the obtained residue was analyzed by RP-HPLC, which was performed with a linear gradient of 0%–10% MeCN for 60 min in a 0.1 M ammonium acetate buffer (pH 7.0) at 30 °C using a column oven. Also, the three tenths of the mixture was purified by RP-HPLC using same conditions described above, and the amount of the isolated products were calculated by UV absorbance. α-isomer of **4**: colorless amorphous solid, 106 nmol, 71%, β-isomer of **4**: colorless amorphous solid, 32 nmol, 21%

Characterization data of compound **4**
^1H NMR (600 MHz, D_2O) α-isomer: δ 7.05–7.00 (m, 4H), 5.43 (dd, *J* = 1.7, 7.6 Hz, 1H), 4.44 (d, *J* = 7.8 Hz, 1H), 4.24–4.17 (m, 4H), 4.12 (t, *J* = 4.4 Hz, 2H), 4.01–4.00 (m, 1H), 3.98–3.95 (m, 1H), 3.94–3.89 (m, 5H), 3.87–3.78 (m, 3H), 3.77–3.72 (m, 2H), 3.66 (dd, *J* = 3.4, 10.0 Hz, 1H), 3.55 (dd, *J* = 7.9, 9.9 Hz, 1H);
β-isomer: δ 7.04–7.01 (m, 4H), 5.11 (d, *J* = 7.9 Hz, 1H), 4.41 (d, *J* = 7.7 Hz, 1H), 4.25–4.21 (m, 4H), 4.12 (t, *J* = 4.7 Hz, 2H), 4.02–4.00 (m, 1H), 3.92–3.89 (m, 4H), 3.82–3.69 (m, 6H), 3.66 (dd. *J* = 3.3, 9.9 Hz, 1H), 3.53 (dd, *J* = 7.7, 9.8 Hz, 1H), 3.50–3.47 (m, 1H);

HRMS (ESI-QTOF) *m/z*: calcd for $[M-H]^-$ $C_{22}H_{34}O_{17}P^-$ 601.1539, found α-isomer; 601.1530, β-isomer; 601.1528;
HPLC (ODS, 0.1 M ammonium acetate buffer/MeCN = 100/0–90/10, flow rate = 0.5 mL/min, detection = 286 nm) tR = 28.6 min (α-isomer), 29.8 min (β-isomer).

Characterization data of compound **6** (colorless amorphous solid)
^{1}H NMR (500 MHz, D_2O): δ 7.05–6.99 (m, 4H), 4.18 (t, *J* = 4.6 Hz, 2H), 4.12 (t, *J* = 4.4 Hz, 2H), 4.07–4.03 (m, 2H), 3.90 (t, *J* = 4.6 Hz, 2H), 3.20 (q, *J* = 7.4 Hz, 6H), 1.28 (t, *J* = 7.3 Hz, 9H);
HRMS (ESI-FT-ICR) *m/z*: calcd for $[M-H]^-$ $C_{10}H_{14}O_7P^-$ 277.0483, found; 277.0492;
HPLC (ODS, 0.1 M ammonium acetate buffer/MeCN = 100/0–90/10 over 60 min, flow rate = 0.5 mL/min, detection = 286 nm) tR = 21.1 min.

Scheme S2. Solid-phase synthesis of disaccharide 1-boranophosphate **S6**.

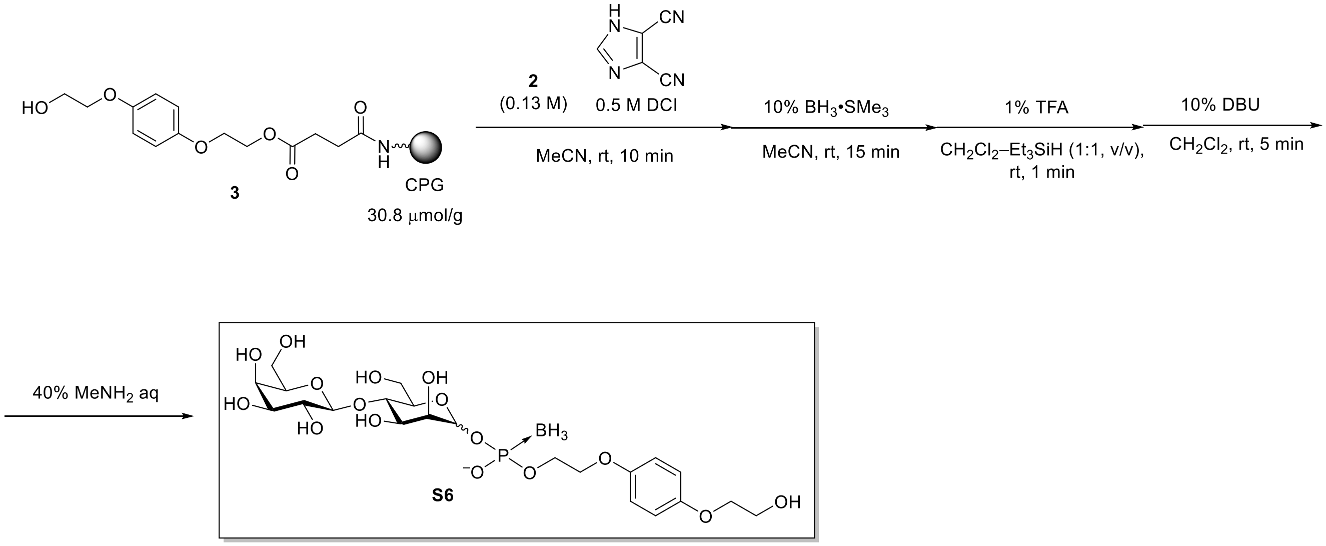

Solid-phase synthesis of disaccharide 1-boranophosphate (S6, Scheme S2)

The CPG-loaded *O*-MMTr protected hydroquinone spacer (30.8 μmol/g, 0.50 μmol), via a succinyl linker (**3**), was treated in a reaction vessel with 1% TFA in dry CH_2Cl_2 (4 × 15 s, 1 mL each) and washed with dry CH_2Cl_2 (4 × 1 mL) and MeCN (3 × 1 mL). Thereafter, it was dried in vacuo for 10 min. Then, the disaccharide 1-phosphoramidite monomer (**2**, α:β = 93:7, 30.9 mg, 20 μmol), which was dried *in vacuo* overnight, was added to the reaction vessel and dried *in vacuo* for 3 min. A 0.5 M solution of DCI (8.9 mg, 75 μmol) in dry MeCN (150 μL), which was dried over MS 3Å overnight, was added under Ar atmosphere to the reaction vessel. After 10 min, the CPG was washed with dry MeCN (3 × 1 mL) and dried in vacuo for 5 min. The resultant phosphite was boronated upon treatment with a $BH_3 \cdot SMe_2$ (100 μL) solution in dry MeCN (900 μL) and the reaction vessel was shaken for 15 min. Then, the CPG was washed with dry MeCN (3 × 1 mL) and dry CH_2Cl_2 (4 × 1 mL) and the detritylation reaction was carried out using 1% TFA in dry CH_2Cl_2–Et_3SiH (1:1, v/v, 4 × 15 s, 1 mL each). Subsequently, a 10% 1,8-diazabicyclo[5.4.0]undec-7-ene solution in MeCN (1 mL, v/v) was added to the reaction vessel. After 5 min, the CPG was washed with MeCN (4 × 1 mL) and then treated with a 40% $MeNH_2$ aqueous solution (4 mL) at rt for 4 h, filtered, and washed with EtOH. The filtrate and the washings were combined, concentrated under reduced pressure, and the obtained residue was analyzed by RP-HPLC, which was performed with a linear gradient of 0%–20% MeCN for 60 min in a 0.1 M triethylammonium acetate buffer (pH 7.0) at 30 °C a column oven. Also, the three tenths of the mixture was purified by RP-HPLC using same conditions described above, and the amount of the isolated product were calculated by UV absorbance. α, β mixture: colorless amorphous solid, 75 nmol, 50%

Characterization data of compound **S6**

^{1}H NMR (600 MHz, D_2O): δ 7.04–7.00 (m, α, β-4H), 5.50–5.46 (m, α-1H, mixture of diastereomers), 5.20–5.16 (m, β-1H, mixture of diastereomers), 4.44 (d, J = 8.2 Hz, α-0.5H, one of diastereomers), 4.44 (d, J = 7.8 Hz, α-0.5H, one of diastereomers), 4.42 (d, J = 8.2 Hz, β-1H), 4.24–4.17 (m, α, β-4H), 4.12 (t, J = 4.6 Hz, α, β-2H), 3.99–3.97 (m, α, β-2H), 3.96–3.89 (m, α, β-5H), 3.86–3.70 (m, α, β-3H), 3.66 (dd. J = 3.4, 10.0 Hz, α, β-1H), 3.55 (dd, J = 7.9, 9.9 Hz, α, β-1H), 3.20 (q, J = 7.3 Hz, α, β-6H), 1.28 (t, J = 7.3 Hz, α, β-9H), 0.6–0.0 (br, α, β-3H);

HRMS (ESI-QTOF) *m/z*: calcd for $[M - H]^-$ $C_{22}H_{37}BO_{16}P^-$, 599.1918, found; 599.1900.

HPLC (ODS, 0.1 M TEAA buffer/MeCN = 100/0–80/20 over 60 min, flow rate = 0.5 mL/min, detection = 286 nm) tR = 38.5–39.9 min.

Solid-phase synthesis of disaccharide 1-phosphate via boranophosphate (Table 1, entry 1, Scheme 3)

The CPG-loaded *O*-MMTr protected hydroquinone spacer (30.8 μmol/g, 0.50 μmol), via a succinyl linker (**3**), was treated in a reaction vessel with 1% TFA in dry CH_2Cl_2 (4 × 15 s, 1 mL each) and washed with dry CH_2Cl_2 (4 × 1 mL) and MeCN (3 × 1 mL). Thereafter, it was dried in vacuo for 10 min. Then, the disaccharide 1-phosphoramidite monomer (**2**, α:β = 93:7, 30.9 mg, 20 μmol), which was dried *in vacuo* overnight, was added to the reaction vessel and dried *in vacuo* for 3 min. A 0.5 M solution of DCI (8.9 mg, 75 μmol) in dry MeCN (150 μL), which was dried over MS 3Å overnight, was added under Ar atmosphere to the reaction vessel. After 10 min, the CPG was washed with dry MeCN (3 × 1 mL) and dried in vacuo for 5 min. The resultant phosphite was boronated upon treatment with a $BH_3 \cdot SMe_2$ (100 μL) solution in dry MeCN (900 μL) and the reaction vessel was shaken for 15 min. Then, the CPG was washed with dry MeCN (3 × 1 mL) and dry CH_2Cl_2 (4 × 1 mL) and the detritylation reaction was carried out using 1% TFA in dry CH_2Cl_2–Et_3SiH (1:1, v/v, 4 × 15 s, 1 mL each). Subsequently, a 10% 1,8-diazabicyclo[5.4.0]undec-7-ene solution in MeCN (1 mL, v/v) was added to the reaction vessel. After 5 min, the CPG was washed with MeCN (4 × 1 mL). Thereafter, a 0.4 M DCSO solution (47.2 mg, 0.16 mmol) in dry MeCN (0.4 mL) was added to the reaction vessel. After 5 h, the solution was removed and the CPG was washed with dry MeCN (3 × 1 mL) and then treated with a 40% $MeNH_2$ aqueous solution (4 mL) at rt for 4 h, filtered, and washed with EtOH. The filtrate and the washings were combined, concentrated under reduced pressure, and the obtained residue was analyzed by RP-HPLC, which was performed with a linear gradient of 0%–10% MeCN for 60 min in a 0.1 M ammonium acetate buffer (pH 7.0) at 30 °C using a column oven.

Investigation for the preactivation conditions (Table 1, entries 2 and 3, Scheme 4)

The CPG-loaded *O*-MMTr protected hydroquinone spacer (26.4 μmol/g, 0.50 μmol), via a succinyl linker (**3**), was used for the experiments. The disaccharide 1-phosphate was synthesized by a similar procedure for Scheme 3, except for the condensation reaction conditions. Prior to the reaction with a hydroxy group on CPG, the disaccharide 1-phosphoramidite monomer (**2**, α:β = 91:9, 37.0 mg, 24 μmol), which was dried *in vacuo* overnight, was treated in a round bottom flask with a solution containing 3-phenyl propanol (3.6 mg, 4.8 μmol), DCI (10.6 mg, 90 μmol) in dry MeCN (180 μL). After stirring for 3 min, a part of the mixture (150 μL) was added to the reaction vessel for the solid-phase synthesis, and left for designated time (10 min for entry 2, 60 min for entry 3). The CPG was washed with dry MeCN (3 × 1 mL) and dried in vacuo for 5 min. The resultant phosphite was boronated upon treatment with a $BH_3 \cdot SMe_2$ (100 μL) solution in dry MeCN (900 μL) and the reaction vessel was shaken for 15 min. Then, the CPG was washed with dry MeCN (3 × 1 mL) and dry CH_2Cl_2 (4 × 1 mL) and the detritylation reaction was carried out using 1% TFA in dry CH_2Cl_2–Et_3SiH (1:1, v/v, 4 × 15 s, 1 mL each). Subsequently, a 10% 1,8-diazabicyclo[5.4.0]undec-7-ene solution in MeCN (1 mL, v/v) was added to the reaction vessel.

After 5 min, the CPG was washed with MeCN (4 × 1 mL). Thereafter, a 0.4 M DCSO solution (23.6 mg, 79 μmol) in dry MeCN (0.2 mL) was added to the reaction vessel. After 5 h, the solution was removed and the CPG was washed with dry MeCN (3 × 1 mL) and then treated with a 40% $MeNH_2$ aqueous solution (4 mL) at rt for 4 h, filtered, and washed with EtOH. The filtrate and the washings were combined, concentrated under reduced pressure, and the obtained residue was analyzed by RP-HPLC, which was performed with a linear gradient of 0%–10% MeCN for 60 min in a 0.1 M ammonium acetate buffer (pH 7.0) at 30 °C using a column oven.

Investigation for activators (Table 2, entries 1–3, Scheme 4)

The CPG-loaded *O*-MMTr protected hydroquinone spacer (26.4 μmol/g, 0.50 μmol), via a succinyl linker (**3**), was treated in a reaction vessel with 1% TFA in dry CH_2Cl_2 (4 × 15 s, 1 mL each) and washed with dry CH_2Cl_2 (4 × 1 mL) and MeCN (3 × 1 mL). Thereafter, it was dried in vacuo for 10 min. Prior to the reaction with a hydroxy group on CPG, the disaccharide 1-phosphoramidite monomer (**2**, α:β = 91:9, 37.0 mg, 24 μmol), which was dried *in vacuo* overnight, was treated in a round bottom flask with a solution containing 3-phenyl propanol (3.6 mg, 4.8 μmol) and activator (DCI, 10.6 mg, 90 μmol for entry 1, DCI, 21.3 mg, 180 μmol for entry 2, *N*-phenylimidazolium triflate (PhIMT), 52.8 mg, 180 μmol for entry 3) in dry MeCN (180 μL). After stirring for 3 min, a part of the mixture (150 μL) was added to the reaction vessel for the solid-phase synthesis, and left for 60 min. Then, the CPG was washed with dry MeCN (3 × 1 mL) and dried in vacuo for 5 min. The resultant phosphite was boronated upon treatment with a $BH_3\cdot SMe_2$ (100 μL) solution in dry MeCN (900 μL) and the reaction vessel was shaken for 15 min. Then, the CPG was washed with dry MeCN (3 × 1 mL). The capping of the unreacted hydroxy group was conducted by treatment with Ac_2O–*N*-methylimidazole–THF (2:1:7, v/v/v, 0.55 mL) for designated time (for entries 1 and 3, 1 min, for entry 2, 30 s) at rt. Then, the CPG was washed with dry THF (3 × 1 mL) and the detritylation reaction was carried out using 1% TFA in dry CH_2Cl_2–Et_3SiH (1:1, v/v, 4 × 15 s, 1 mL each). The condensation, boronation, and detritylation steps were conducted once more. Subsequently, a 10% 1,8-diazabicyclo[5.4.0]undec-7-ene solution in MeCN (1 mL, v/v) was added to the reaction vessel. After 5 min, the CPG was washed with MeCN (4 × 1 mL). Thereafter, a 0.4 M DCSO solution (23.6 mg, 79 μmol) in dry MeCN (0.2 mL) was added to the reaction vessel. After 5 h, the solution was removed and the CPG was washed with dry MeCN (3 × 1 mL) and then treated with a 40% $MeNH_2$ aqueous solution (4 mL) at rt for 4 h, filtered, and washed with EtOH. The filtrate and the washings were combined, concentrated under reduced pressure, and the obtained residue was analyzed by RP-HPLC, which was performed with a linear gradient of 0%–10% MeCN for 60 min in 0.1 M ammonium acetate buffer (pH 7.0) at 30 °C using a column oven. For entry 3, a fifth of the mixture was purified by RP-HPLC using same conditions described above, and the amount of the isolated product was calculated by UV absorbance. The obtained compound **7** was colorless amorphous solid, 59 nmol, 59%.

Characterization data of compound **7**
^{1}H NMR (600 MHz, D_2O): δ 7.04–7.00 (m, 4H), 5.45–5.42 (m, 2H), 4.46 (d, J = 8.0 Hz, 1H), 4.44 (d, J = 7.8 Hz, 1H), 4.24–4.18 (m, 4H), 4.12 (t, J = 4.6 Hz, 2H), 4.07–4.04 (m, 3H), 4.03–4.00 (m, 2H), 3.99–3.79 (m, 15H), 3.76–3.72 (m, 2H), 3.70–3.64 (m, 2H), 3.55 (dd, J = 7.9, 10.0 Hz, 2H);
HRMS (ESI-QTOF) *m/z*: calcd for $[M - 2H]^{2-}$ $C_{34}H_{54}O_{30}P_2^{2-}$, 502.1093, found; 502.1080.
HPLC (ODS, 0.1 M ammonium acetate buffer/MeCN = 100/0–90/10, flow rate = 0.5 mL/min, detection = 286 nm) tR = 23.6 min

General procedures for the synthesis of repeating units (n = 4 and 5, Table 2 entries 4 and 5, Scheme 4)

The CPG-loaded *O*-MMTr protected hydroquinone spacer (for entry 4, 26.4 μmol/g, 0.50 μmol, for entry 5, 24.8 μmol/g, 0.50 μmol), via a succinyl linker (3), was treated in a reaction vessel with 1% TFA

in dry CH_2Cl_2 (4 × 15 s, 1 mL each) and washed with dry CH_2Cl_2 (4 × 1 mL) and MeCN (3 × 1 mL). Thereafter, it was dried in vacuo for 10 min. Prior to the reaction with a hydroxy group on CPG, the disaccharide 1-phosphoramidite monomer (**2**, for entry 4, α:β = 91:9, for entry 5, α:β = 89:11, 37.0 mg, 24 μmol), which was dried *in vacuo* overnight, was treated in a round bottom flask with a solution containing 3-phenyl propanol (3.6 mg, 4.8 μmol), PhIMT (52.8 mg, 180 μmol) in dry MeCN (180 μL). After stirring for 3 min, a part of the mixture (150 μL) was added to the reaction vessel for the solid-phase synthesis, and left for 60 min. Then, the CPG was washed with dry MeCN (3 × 1 mL) and dried in vacuo for 2 min. The resultant phosphite was boronated upon treatment with a $BH_3 \cdot SMe_2$ (100 μL) solution in dry MeCN (900 μL) and the reaction vessel was shaken for 15 min. Then, the CPG was washed with dry MeCN (3 × 1 mL) and MeOH (4 × 1 mL). The capping of the unreacted hydroxy group was conducted by treatment with Ac_2O–*N*-methylimidazole–THF (2:1:7, v/v/v, 0.55 mL) for 1 min at rt. Then, the CPG was washed with dry THF (3 × 1 mL) and the detritylation reaction was carried out using 1% TFA in dry CH_2Cl_2–Et_3SiH (1:1, v/v, 4 × 15 s, 1 mL each). The condensation, boronation, capping, and detritylation steps were further conducted for designated number of times (for entry 4, twice, for entry 5, three times). Thereafter, the condensation, boronation, and detritylation steps were further conducted for once. Subsequently, a 10% 1,8-diazabicyclo[5.4.0]undec-7-ene solution in MeCN (1 mL, v/v) was added to the reaction vessel. After 5 min, the CPG was washed with MeCN (4 × 1 mL). Thereafter, a 0.4 M DCSO solution (23.6 mg, 79 μmol) in dry MeCN (0.2 mL) was added to the reaction vessel. After 5 h, the solution was removed and the CPG was washed with dry MeCN (3 × 1 mL) and then treated with a 40% $MeNH_2$ aqueous solution (4 mL) at rt for 4 h, filtered, and washed with EtOH. The filtrate and the washings were combined, concentrated under reduced pressure, and the obtained residue was analyzed by RP-HPLC, which was performed with a linear gradient of 5%–23% MeOH for 30 min in an aqueous solution containing 0.1 M 1,1,1,3,3,3-hexafluoro-2-propanol and 8 mM Et_3N at 60 °C using a column oven. Also, a fifth of the mixture was purified by RP-HPLC using same conditions described above, and the amount of the isolated product was calculated by UV absorbance. **8** (n = 4): colorless amorphous solid, 46 nmol, 46%, **9** (n = 5): colorless amorphous solid, 36 nmol, 36%

Characterization data for compound **8**:
^{1}H NMR: δ 7.04–7.00 (m, 4H), 5.45–5.42 (m, 4H), 4.48–4.44 (m, 4H), 4.24–4.18 (m, 4H), 4.12 (t, *J* = 4.5 Hz, 2H), 4.07–3.79 (m, 40H), 3.77–3.73 (m, 2H), 3.70–3.65 (m, 4H), 3.58–3.54 (m, 4H), 3.19 (t, *J* = 7.3 Hz, 24H), 1.27 (t, *J* = 7.3 Hz, 36H).
HRMS (ESI-QTOF) *m/z*: calcd for $[M - 4H]^{4-}$ $C_{58}H_{94}O_{56}P_4^{4-}$, 452.5870, found; 452.5873.
HPLC (ODS, H_2O (containing 0.1 M 1,1,1,3,3,3-hexafluoro-2-propanol and 8 mM Et_3N)/MeCN = 95/5–77/23, flow rate = 0.5 mL/min, detection = 286 nm) tR = 6.1 min.

Characterization data for compound **9**:
^{1}H NMR: δ 7.04–7.00 (m, 4H), 5.46–5.42 (m, 5H), 4.48–4.43 (m, 5H), 4.24–4.18 (m, 4H), 4.12 (t, *J* = 4.5 Hz, 2H), 4.08–3.79 (m, 50H), 3.77–3.73 (m, 2H), 3.70–3.65 (m, 5H), 3.58–3.53 (m, 5H), 3.20 (t, *J* = 7.3 Hz, 30H), 1.27 (t, *J* = 7.3 Hz, 45H).
HRMS (ESI-QTOF) *m/z*: calcd for $[M - 5H]^{5-}$ $C_{70}H_{114}O_{69}P_5^{5-}$, 442.6825, found; 442.6826.
HPLC (ODS, H_2O (containing 0.1 M 1,1,1,3,3,3-hexafluoro-2-propanol and 8 mM Et_3N)/MeCN = 95/5–77/23, flow rate = 0.5 mL/min, detection = 286 nm) tR = 6.4 min.

<Reference of SI>

(1) Hara, R. I.; Yaoita, A.; Takeda, K.; Ueki, H.; Ishii, A.; Imoto, H.; Kobayashi, S.; Sano, M.; Noro, M.; Sato, K.; Wada, T. Solid-Phase Synthesis of Fluorinated Analogues of Glycosyl 1-Phosphate Repeating Structures from Leishmania Using the Phosphoramidite Method. *ChemistryOpen* **2018**, *7* (6), 439–446. https://doi.org/10.1002/open.201800030.

(2) Bhaduri, S.; Pohl, N. L. B. Fluorous-Tag Assisted Syntheses of Sulfated Keratan Sulfate Oligosaccharide Fragments. *Organic Letters* **2016**, *18* (6), 1414–1417. https://doi.org/10.1021/acs.orglett.6b00344.

3. RP-HPLC profiles of crude disaccharide 1-phosphate and boranophosphate

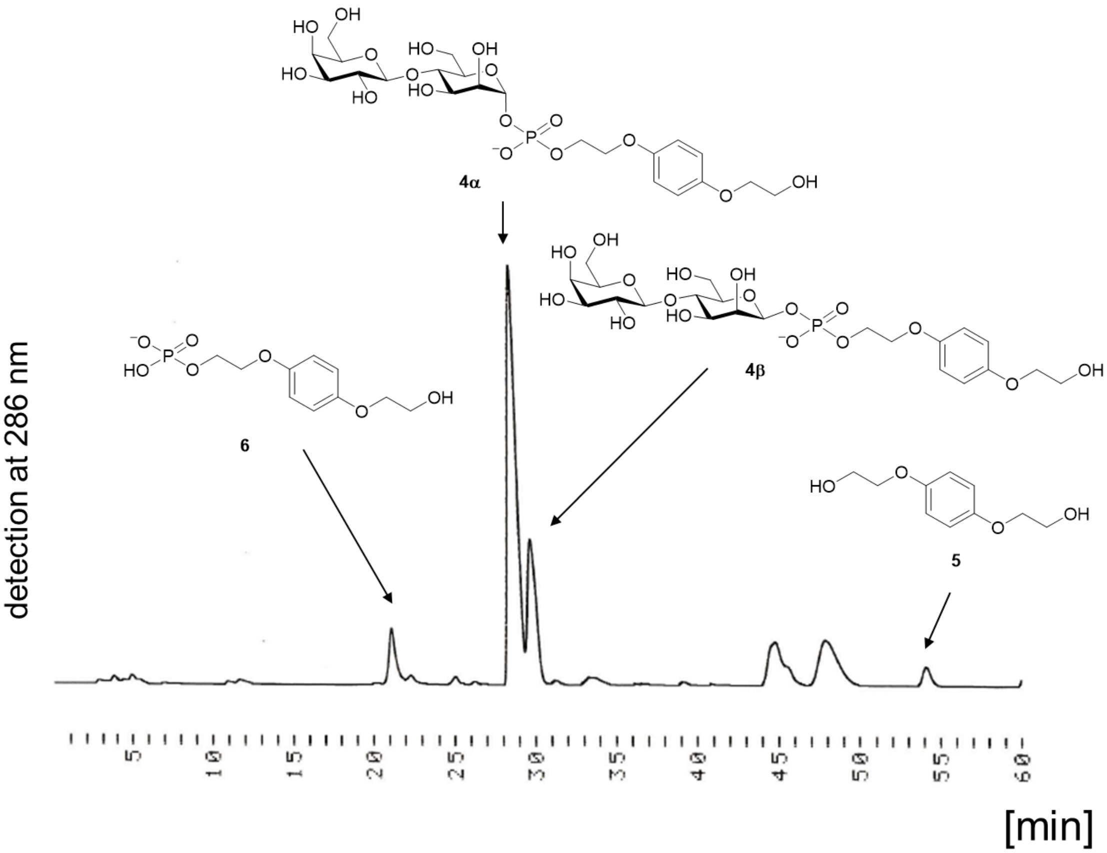

4α:**4β**:**5**:**6** = 67:23:3:7

Figure S1. RP-HPLC profile of the crude mixture of **4** with detection at 286 nm (Scheme 2). RP-HPLC was performed with a linear gradient of 0%–10% MeCN for 60 min in 0.1 M ammonium acetate buffer (pH 7.0) at 30 °C using a C18 column.

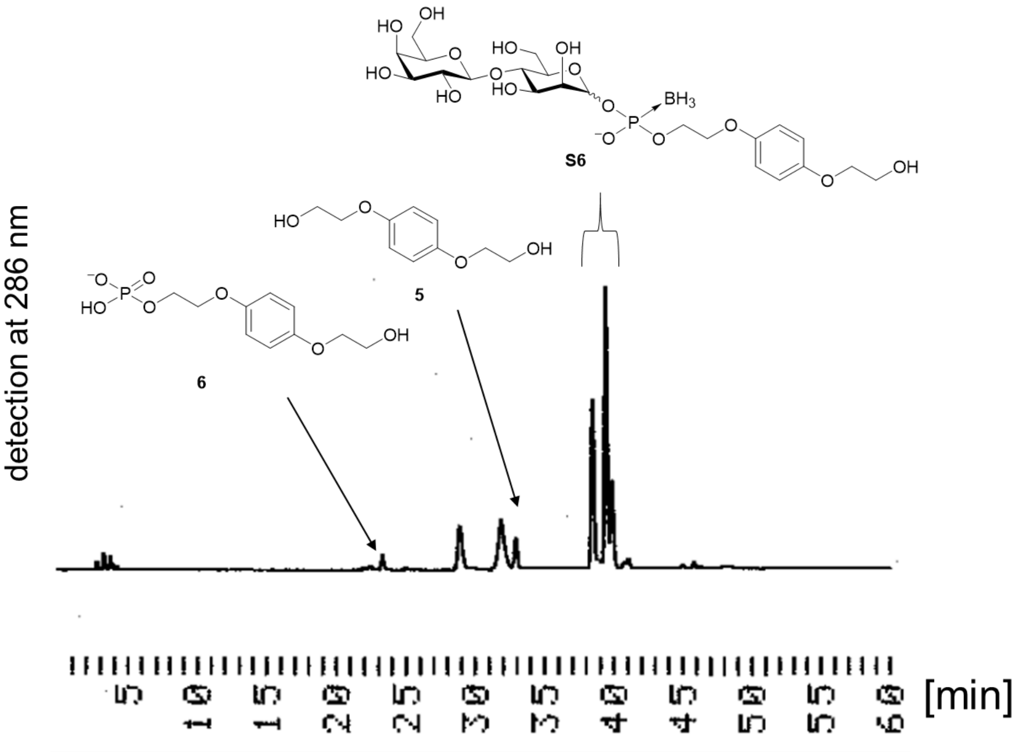

Figure S2. RP-HPLC profile of the crude mixture of **S6** with detection at 286 nm (Scheme S1). RP-HPLC was performed with a linear gradient of 0%–20% MeCN for 60 min in 0.1 M triethylammonium acetate buffer (pH 7.0) at 30 °C using a C18 column. Compound S6 was eluted as a mixture of diastereomers derived from the mannose anomeric position and the phosphorus atom.

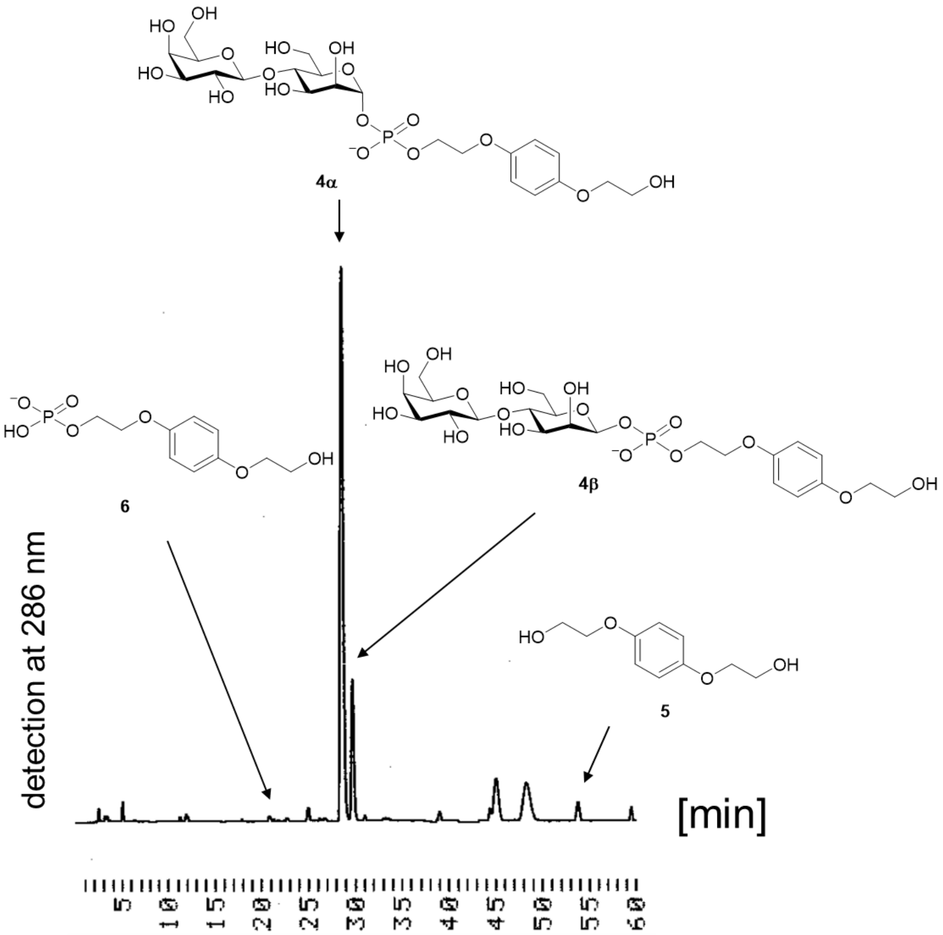

Figure S3. RP-HPLC profile of the crude mixture of **4** with detection at 286 nm (Scheme 3). RP-HPLC was performed with a linear gradient of 0%–10% MeCN for 60 min in 0.1 M ammonium acetate buffer (pH 7) at 30 °C using a C18 column.

Table 1, entry 2

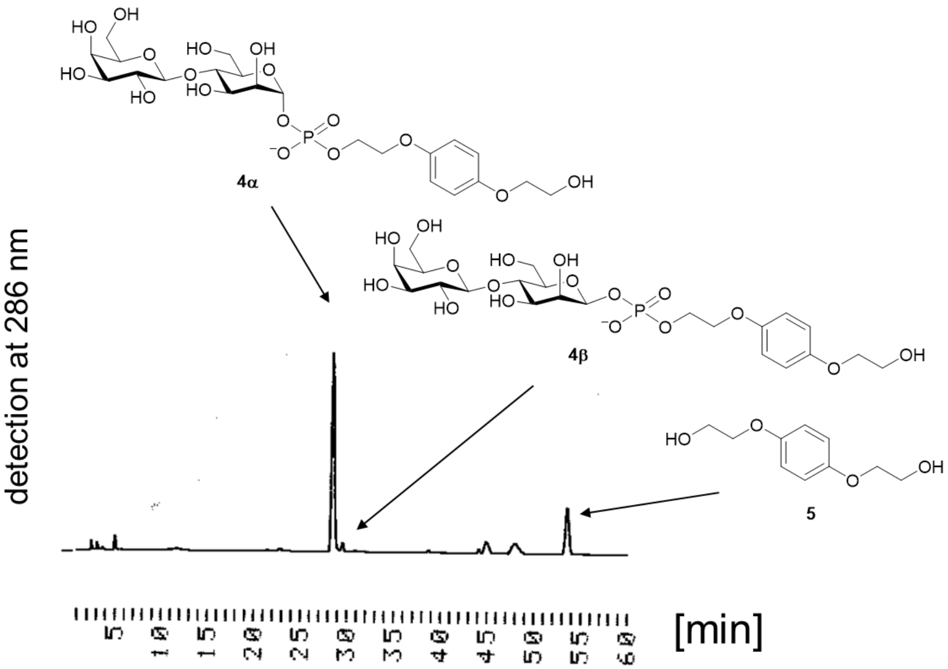

Table 1, entry 3

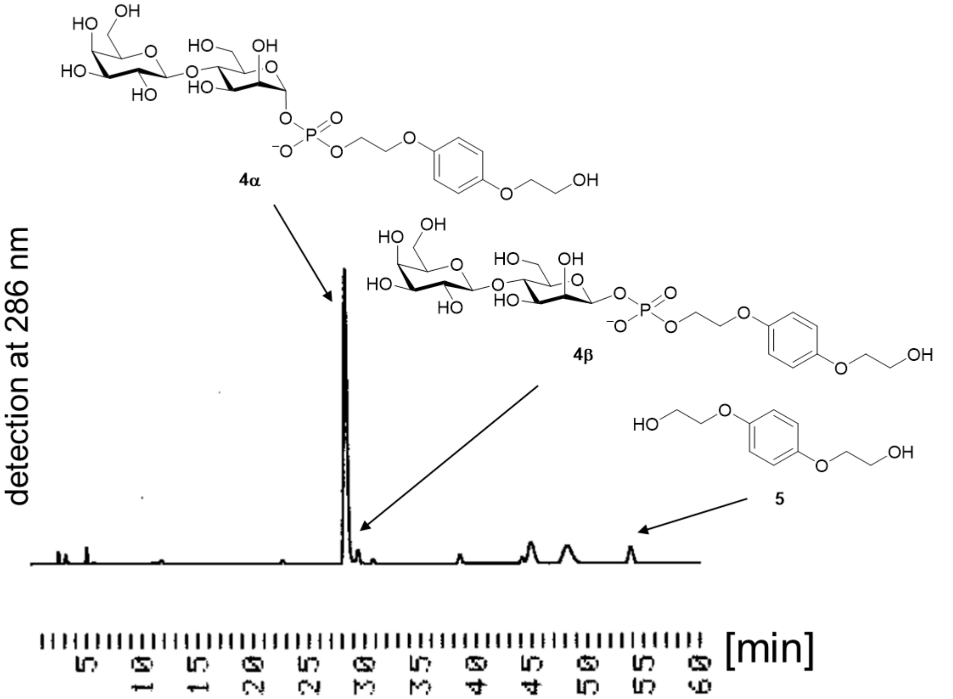

Figure S4. RP-HPLC profiles of the crude mixture of **4** with detection at 286 nm (Table 1). RP-HPLC was performed with a linear gradient of 0%–10% MeCN for 60 min in 0.1 M ammonium acetate buffer (pH 7) at 30 °C using a C18 column.

4. RP-HPLC profiles of crude tetrasaccharide 1-phosphate

Table 2, entry 1

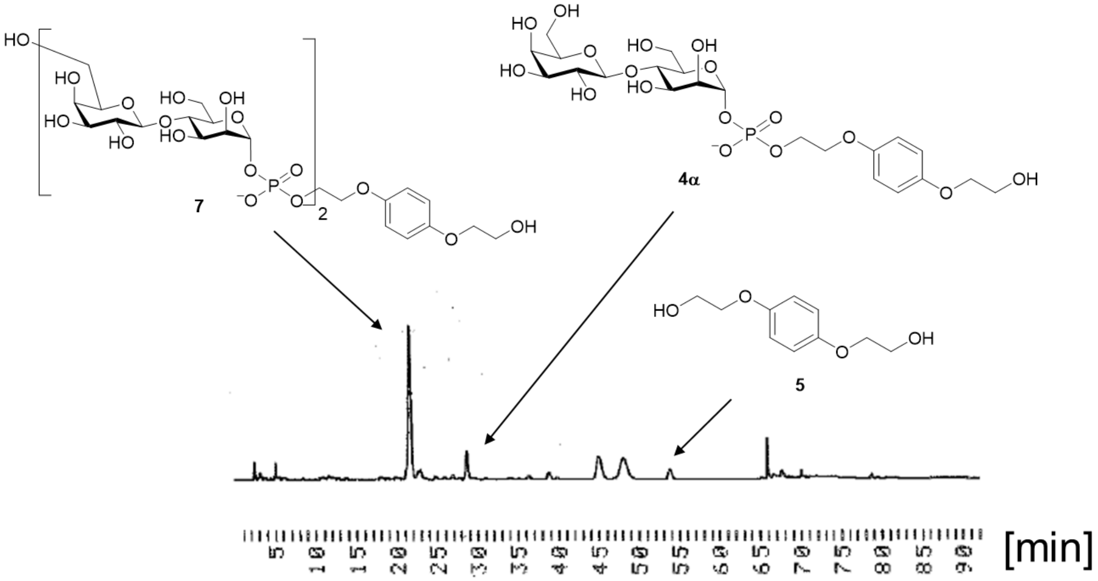

Table 2, entry 2

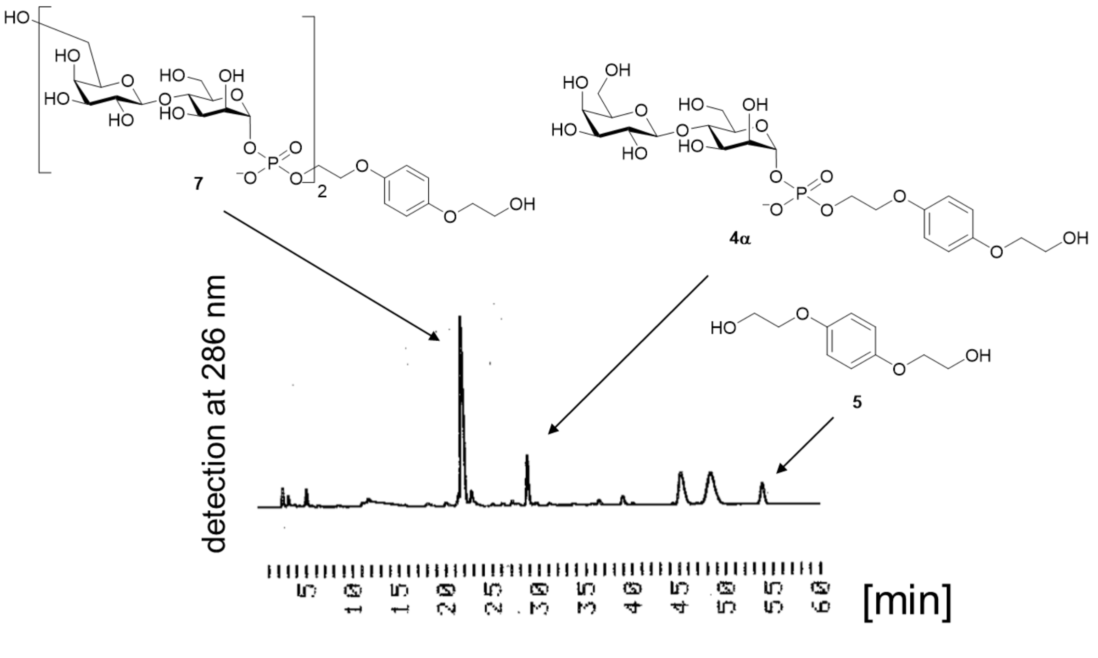

Table 2, entry 3

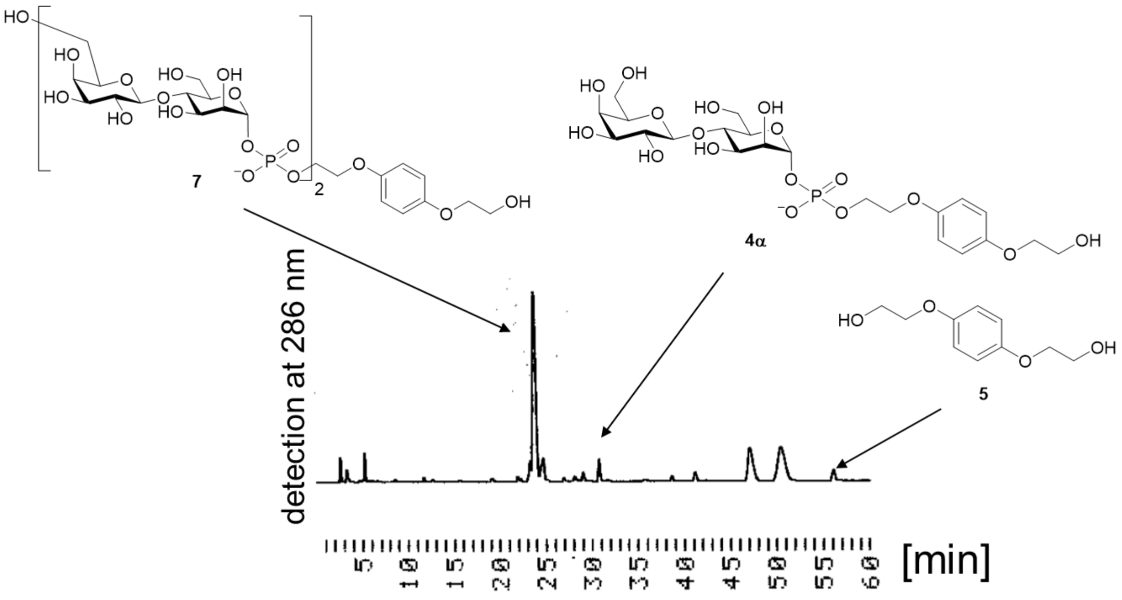

Figure S5. RP-HPLC profiles of the crude mixture of **7** with detection at 286 nm (Table 3). RP-HPLC was performed with a linear gradient of 0%–10% MeCN for 60 min in 0.1 M ammonium acetate buffer (pH 7) at 30 °C using a C18 column.

5. RP-HPLC profiles of crude octasaccharide and decasaccharide 1-phosphates

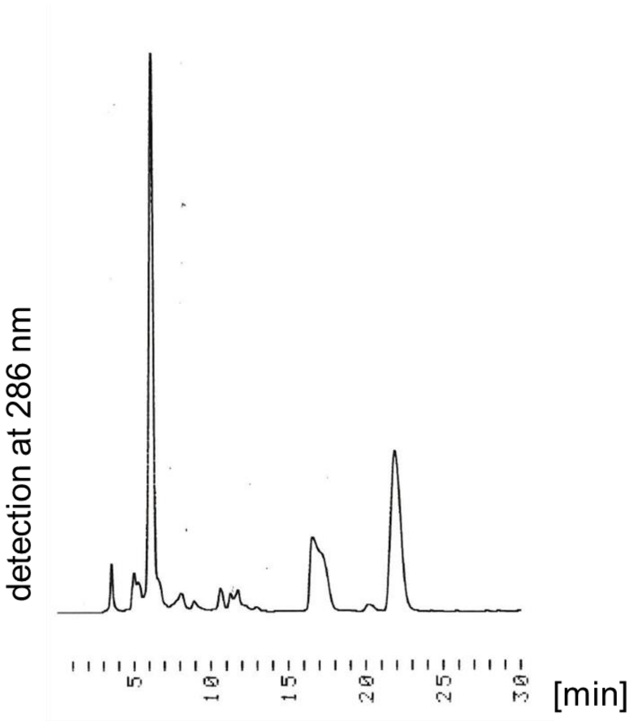

Figure S6. RP-HPLC profiles of the crude mixture of **8** with detection at 286 nm. RP-HPLC was performed with a linear gradient of 5%–23% MeOH for 30 min in an aqueous solution containing 0.1 M 1,1,1,3,3,3-hexafluoro-2-propanol and 8 mM Et_3N at 60 °C using a C18 column. Compound **8** was eluted at 6.4 min.

6. ^{1}H, ^{13}C, ^{31}P, COSY, HMQC, HSQC, HMBC NMR spectra

^{1}H NMR (500 MHz, $CDCl_3$)

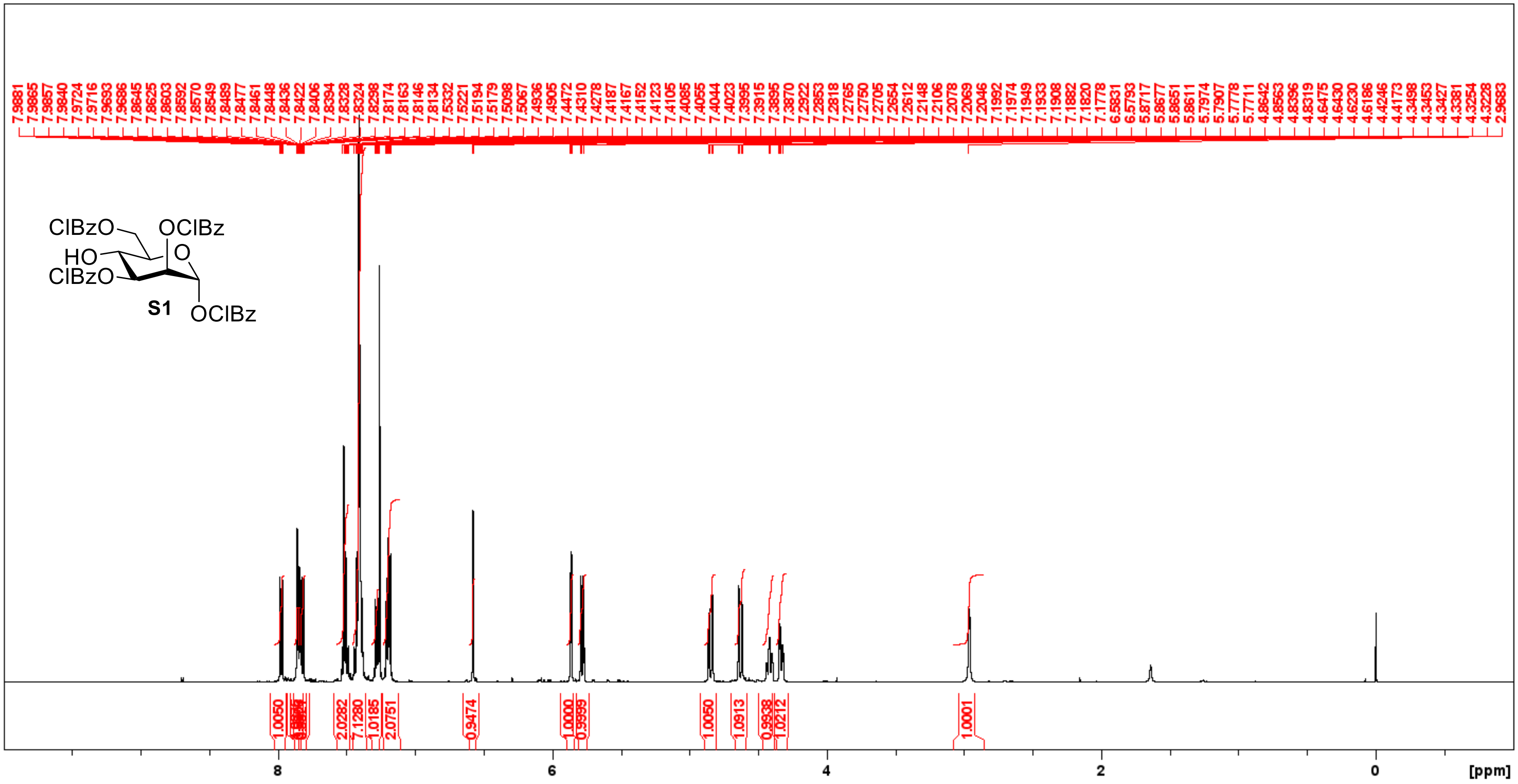

$^{13}C\ \{^1H\}$ NMR (126 MHz, $CDCl_3$)

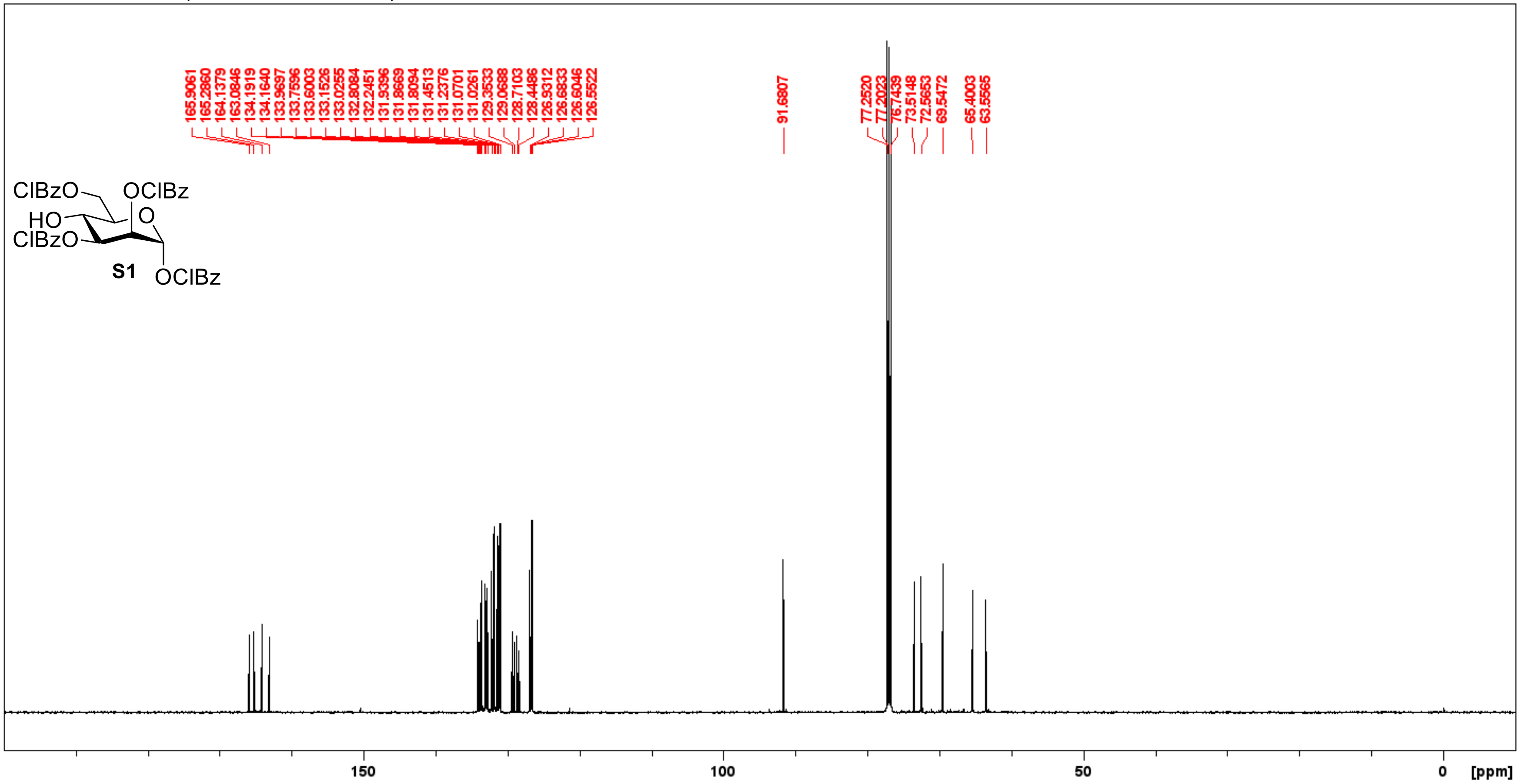

COSY ($CDCl_3$)

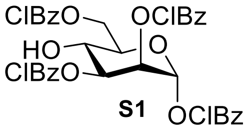

S1

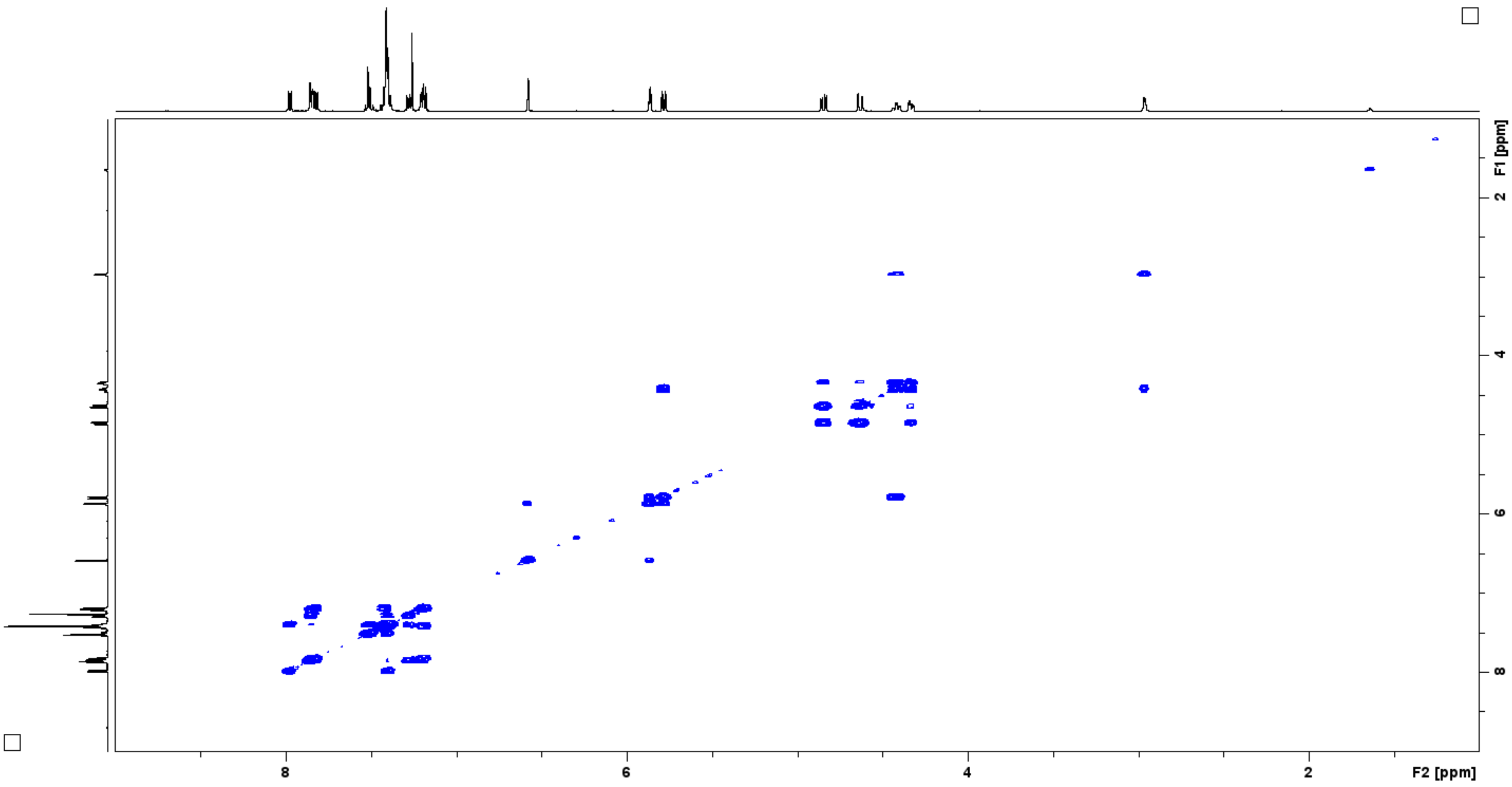

HSQC ($CDCl_3$)

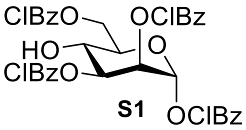

S1

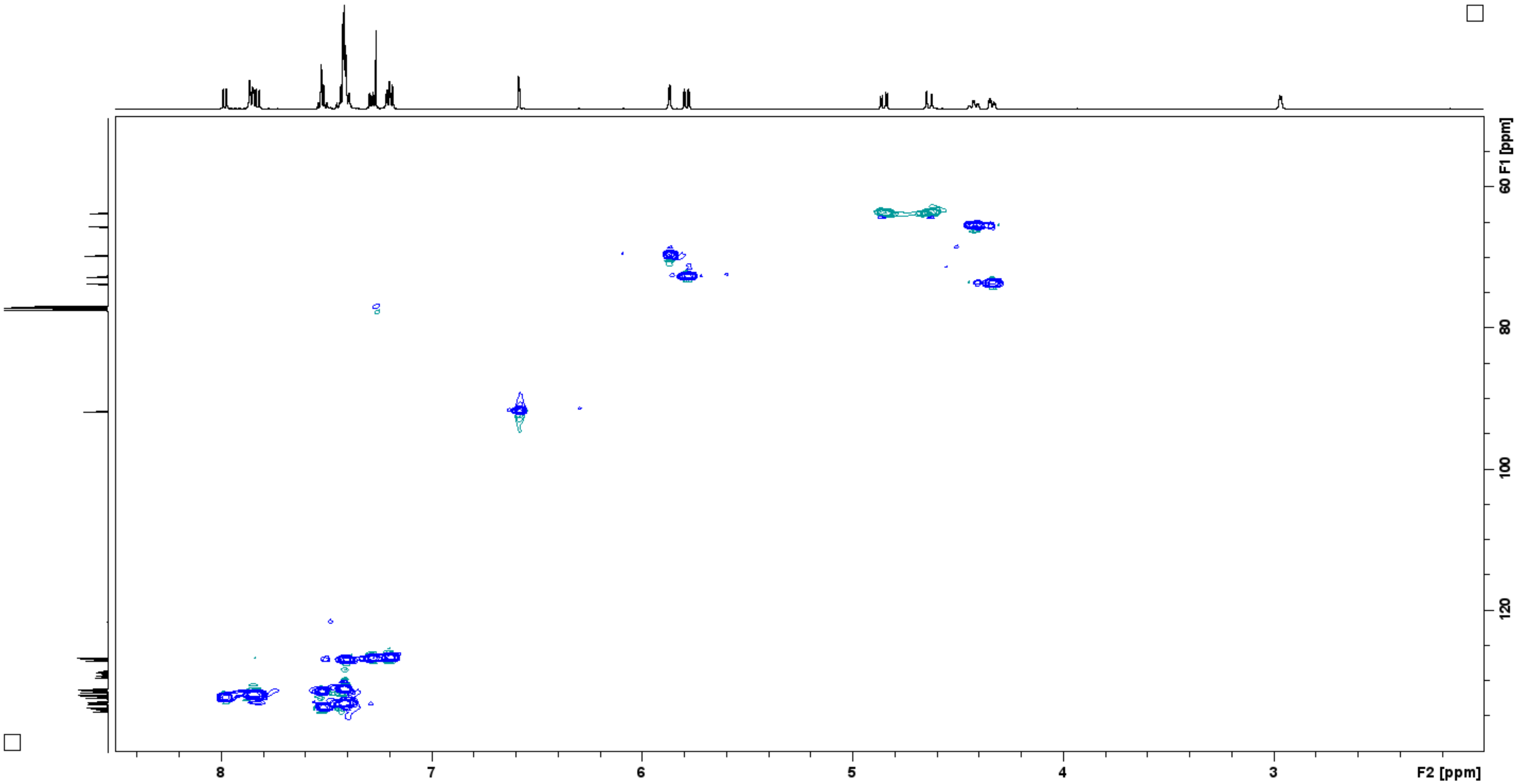

^{1}H NMR (400 MHz, CDCl$_3$)

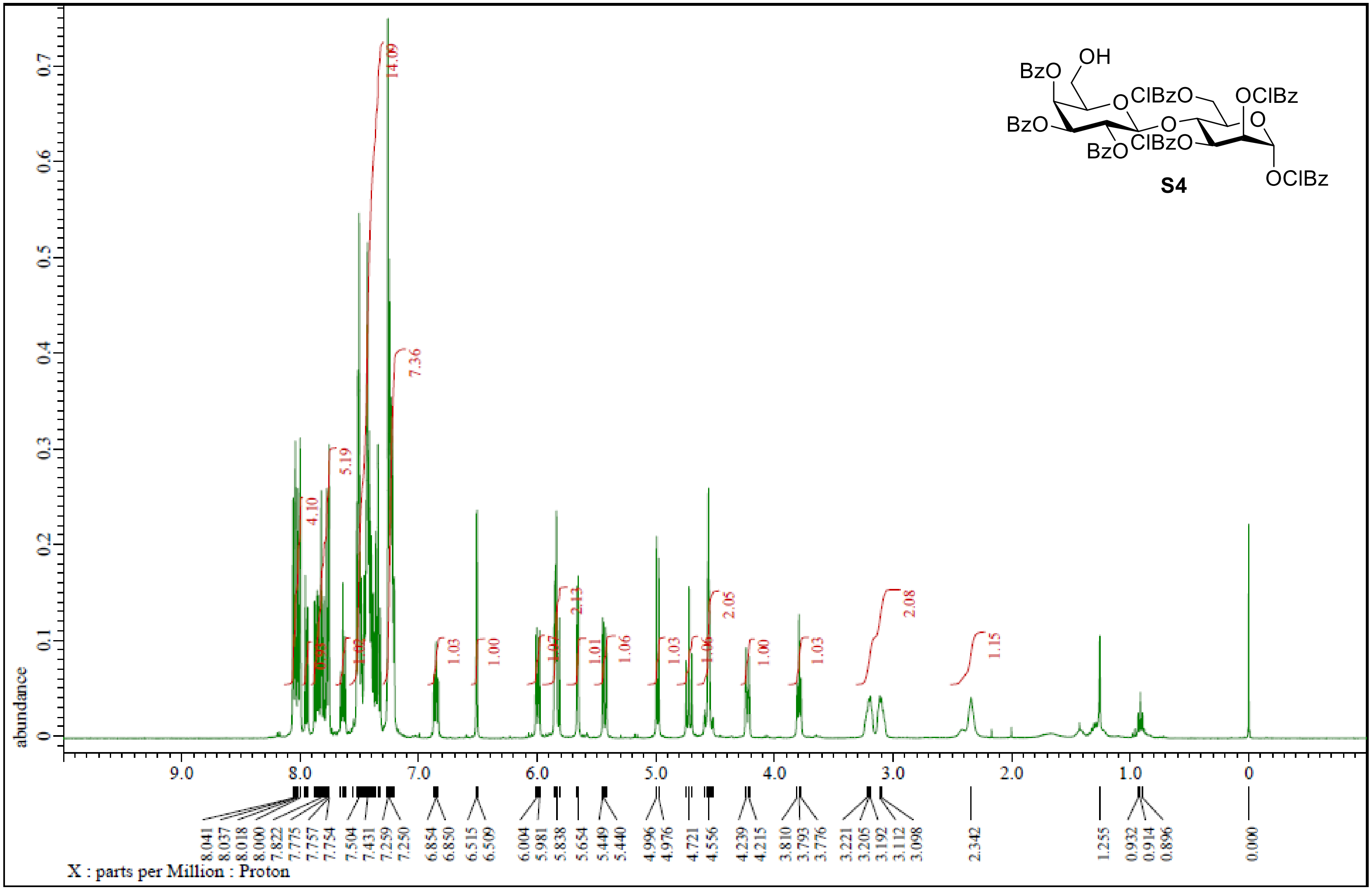

^{13}C {^{1}H} NMR (101 MHz, $CDCl_3$)

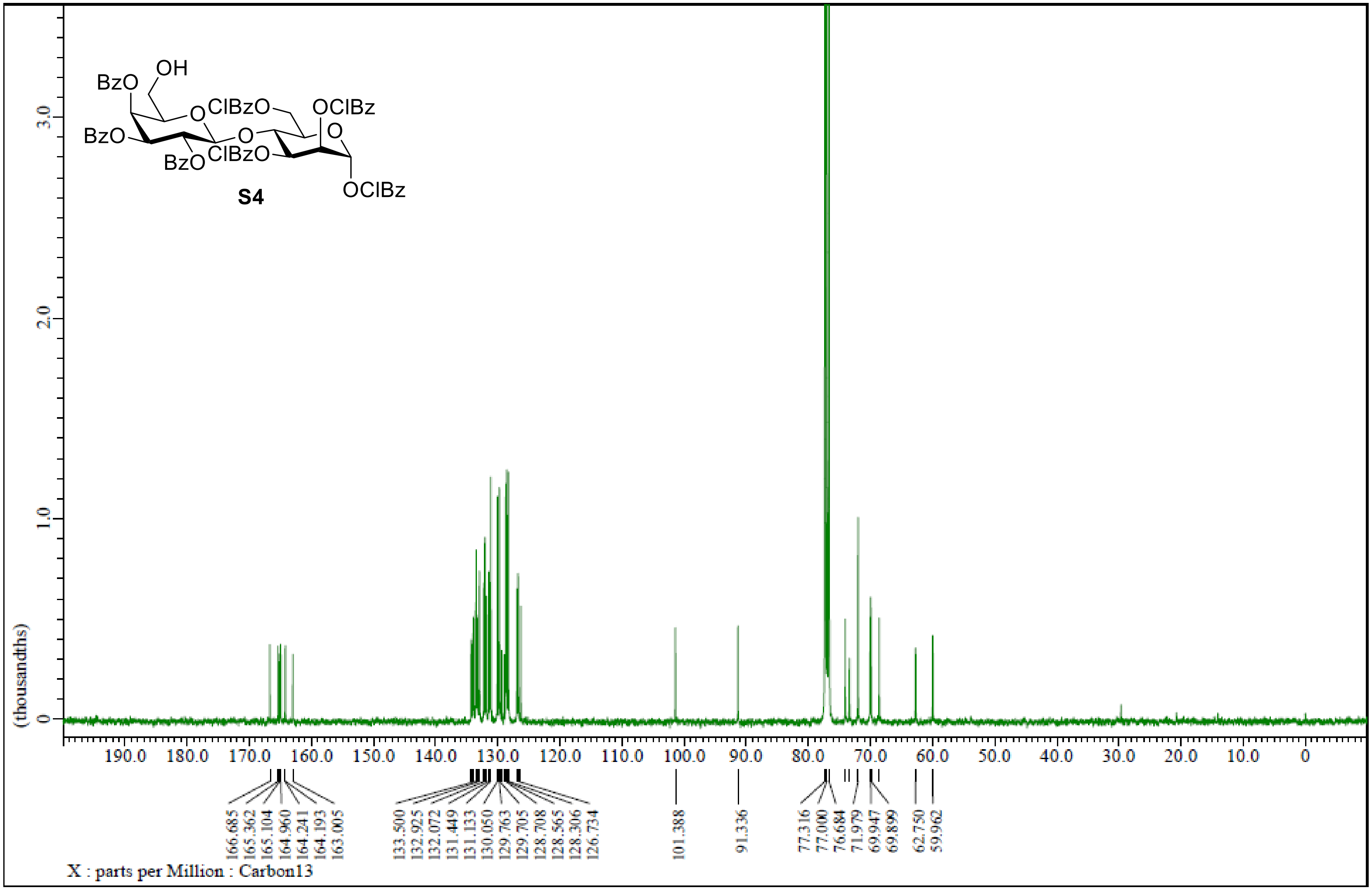

COSY ($CDCl_3$)

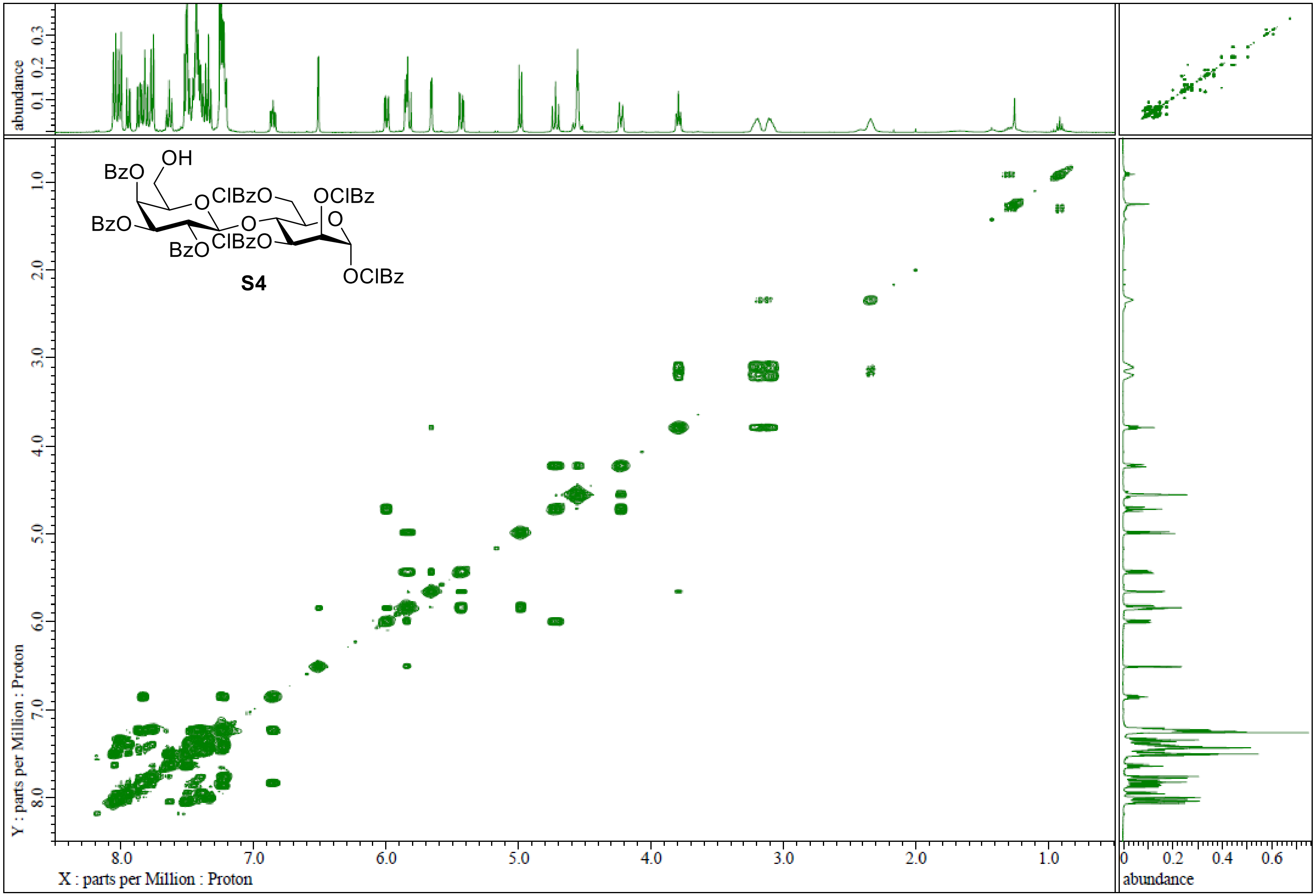
OH
BzO
BzO
BzO
O
ClBzO
O
ClBzO
OClBz
O
OClBz
S4
abundance
0.1
0.2
0.3
1.0
2.0
3.0
4.0
5.0
6.0
7.0
8.0
Y : parts per Million : Proton
X : parts per Million : Proton
0
0.4
0.6
abundance

HMQC (CDCl3)

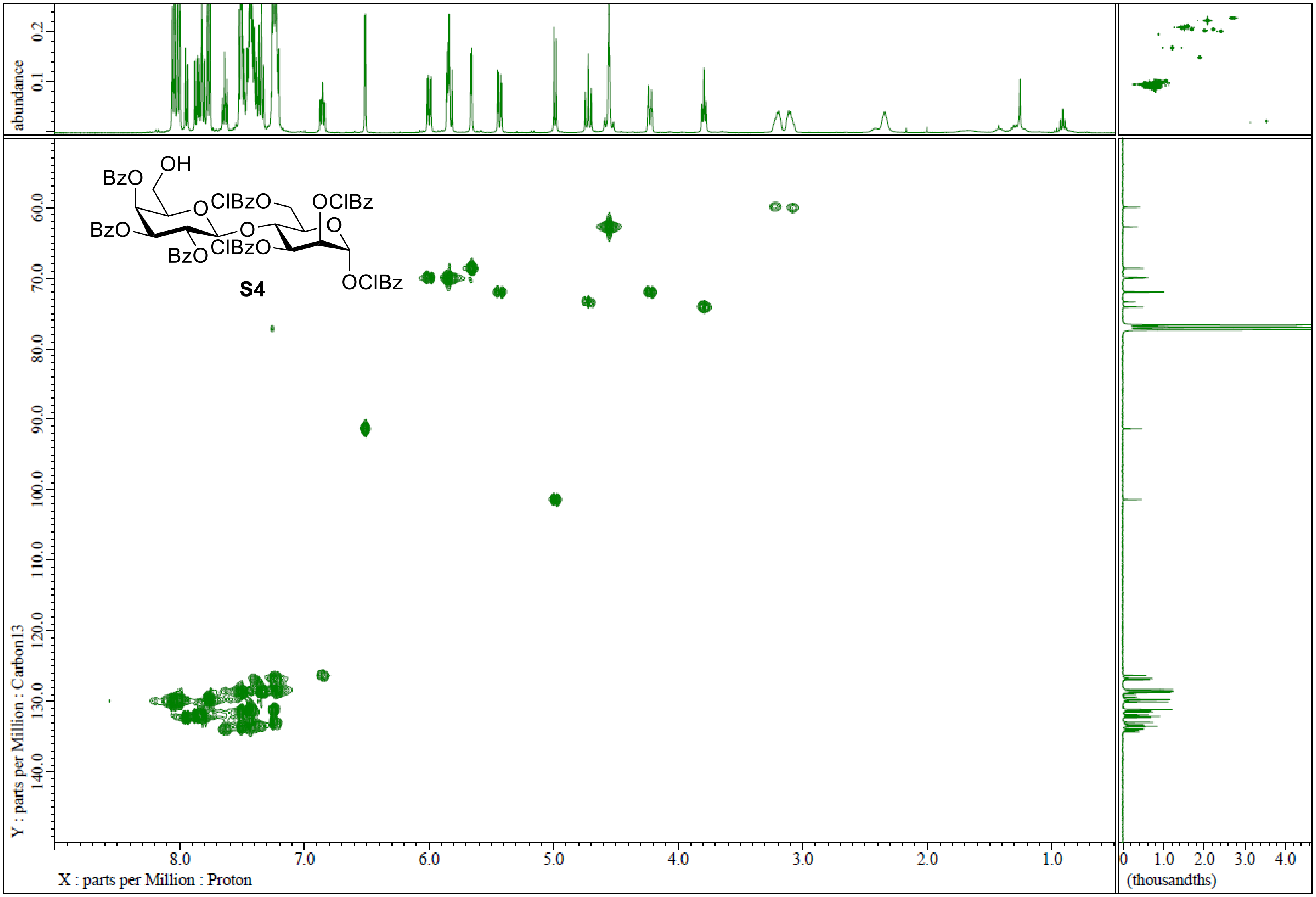

abundance
0.2
0.1
BzO
OH
O
ClBzO
OCIBz
BzO
O
O
BzO
ClBzO
OCIBz
S4
Y : parts per Million : Carbon13
60.0
70.0
80.0
90.0
100.0
110.0
120.0
130.0
140.0
8.0
7.0
6.0
5.0
4.0
3.0
2.0
1.0
X : parts per Million : Proton
0
1.0
2.0
3.0
4.0
(thousandths)

HMQC (CDCl$_3$, Zoom)

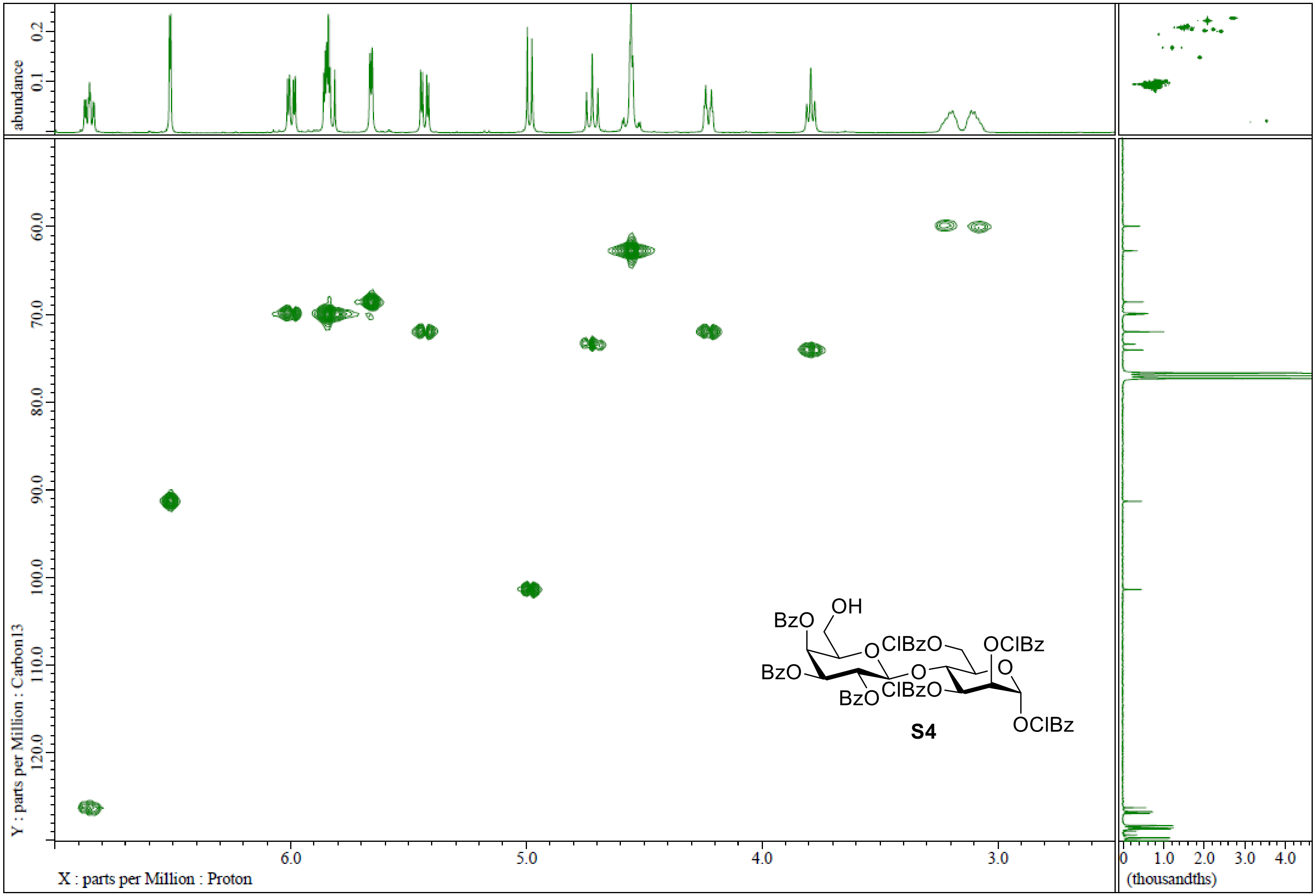

HMBC ($CDCl_3$)

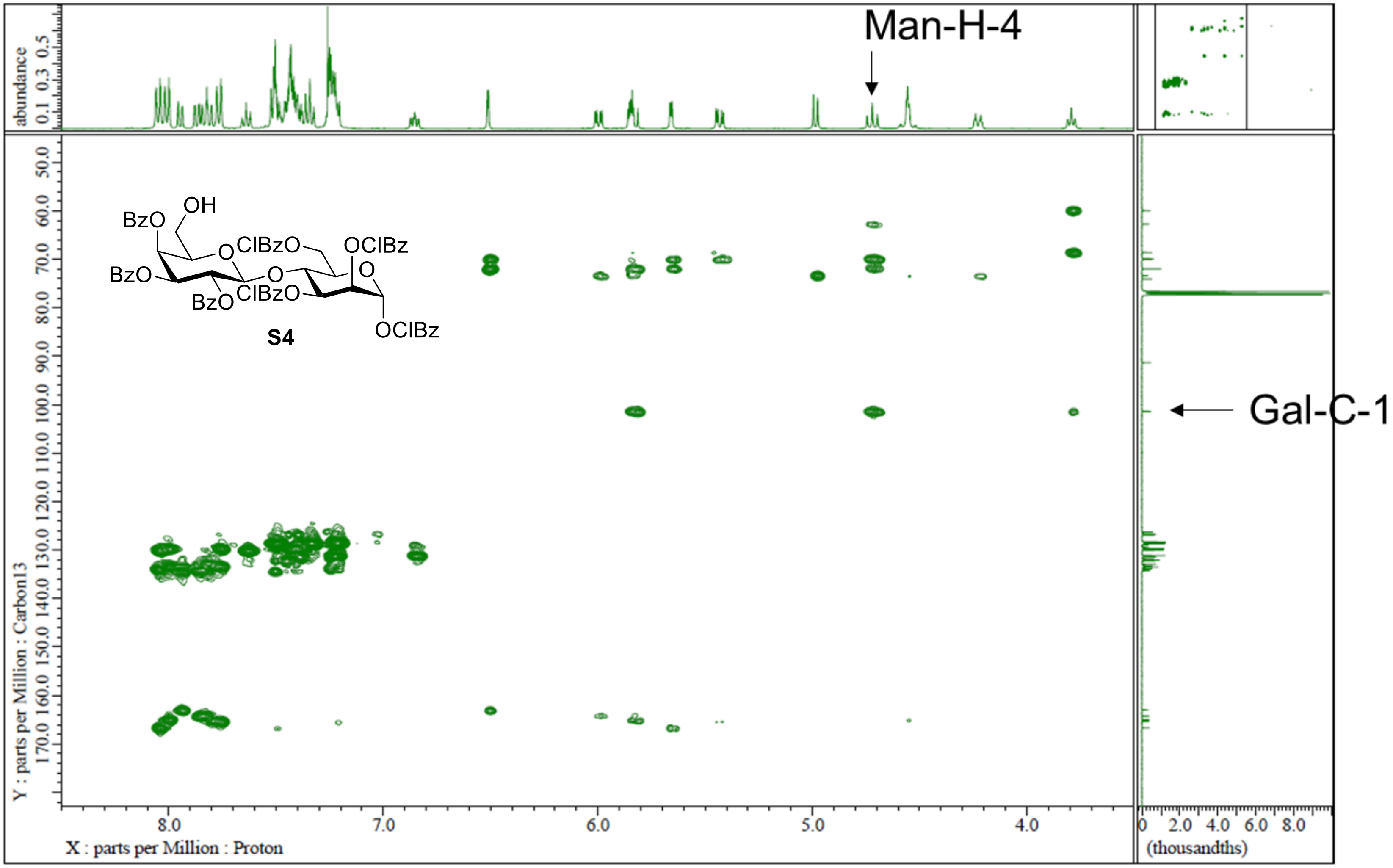
Man-H-4
Gal-C-1
BzO
OH
BzO
O
ClBzO
OClBz
O
O
BzO
ClBzO
OClBz
S4
abundance
Y : parts per Million : Carbon13
X : parts per Million : Proton
(thousandths)

^{1}H NMR (400 MHz, $CDCl_3$)

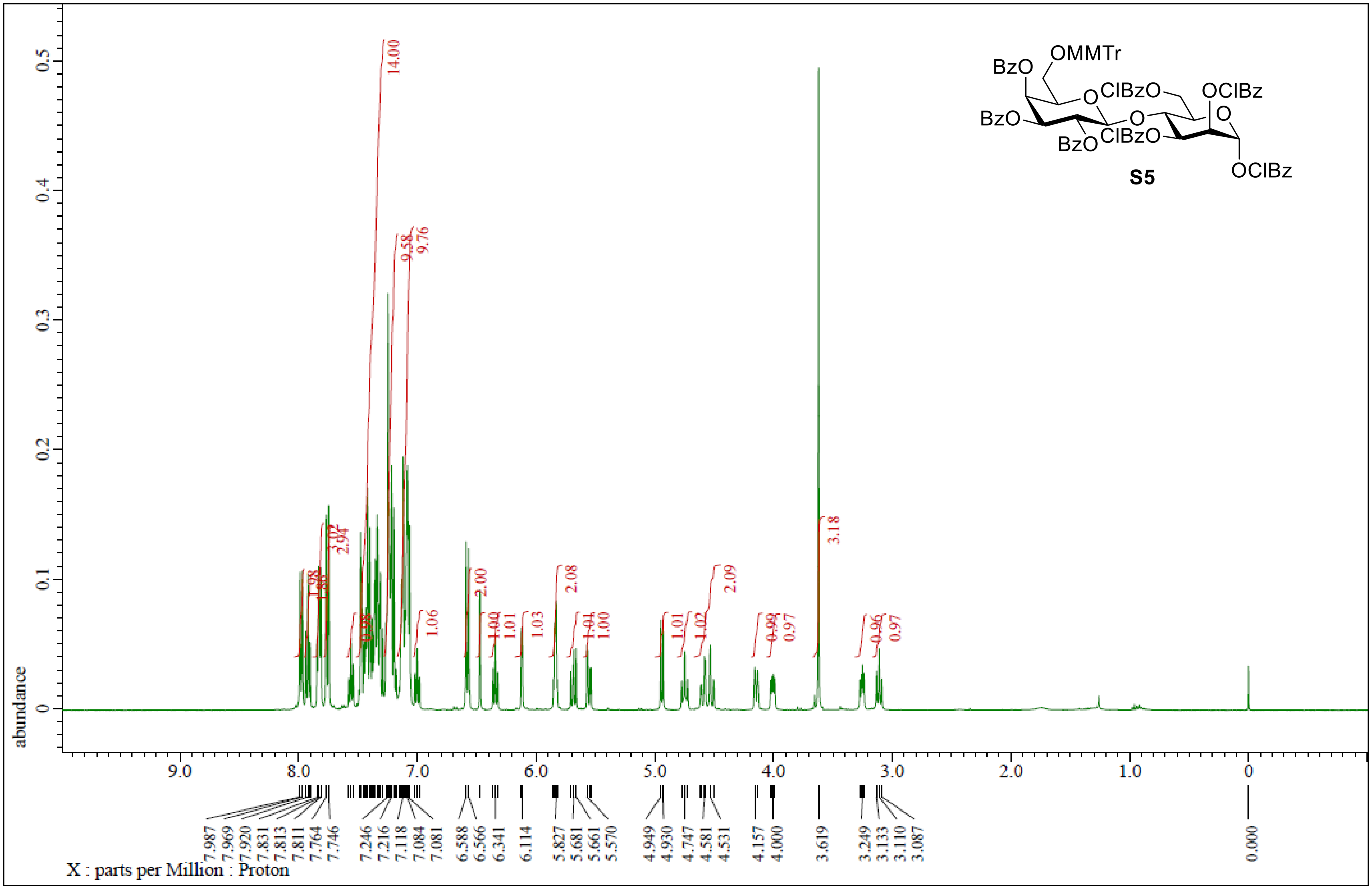

^{13}C {^{1}H} NMR (101 MHz, $CDCl_3$)

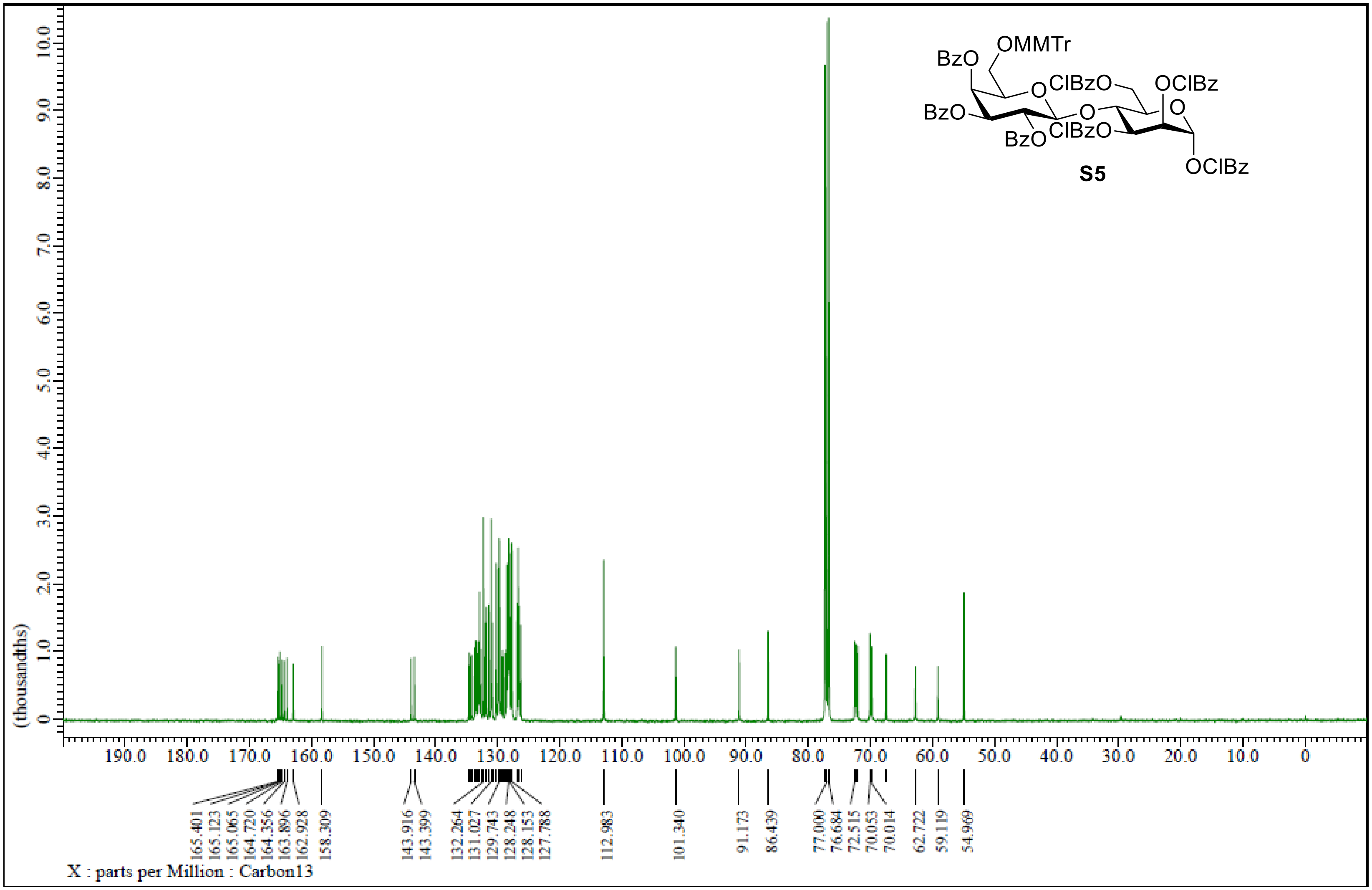

COSY ($CDCl_3$)

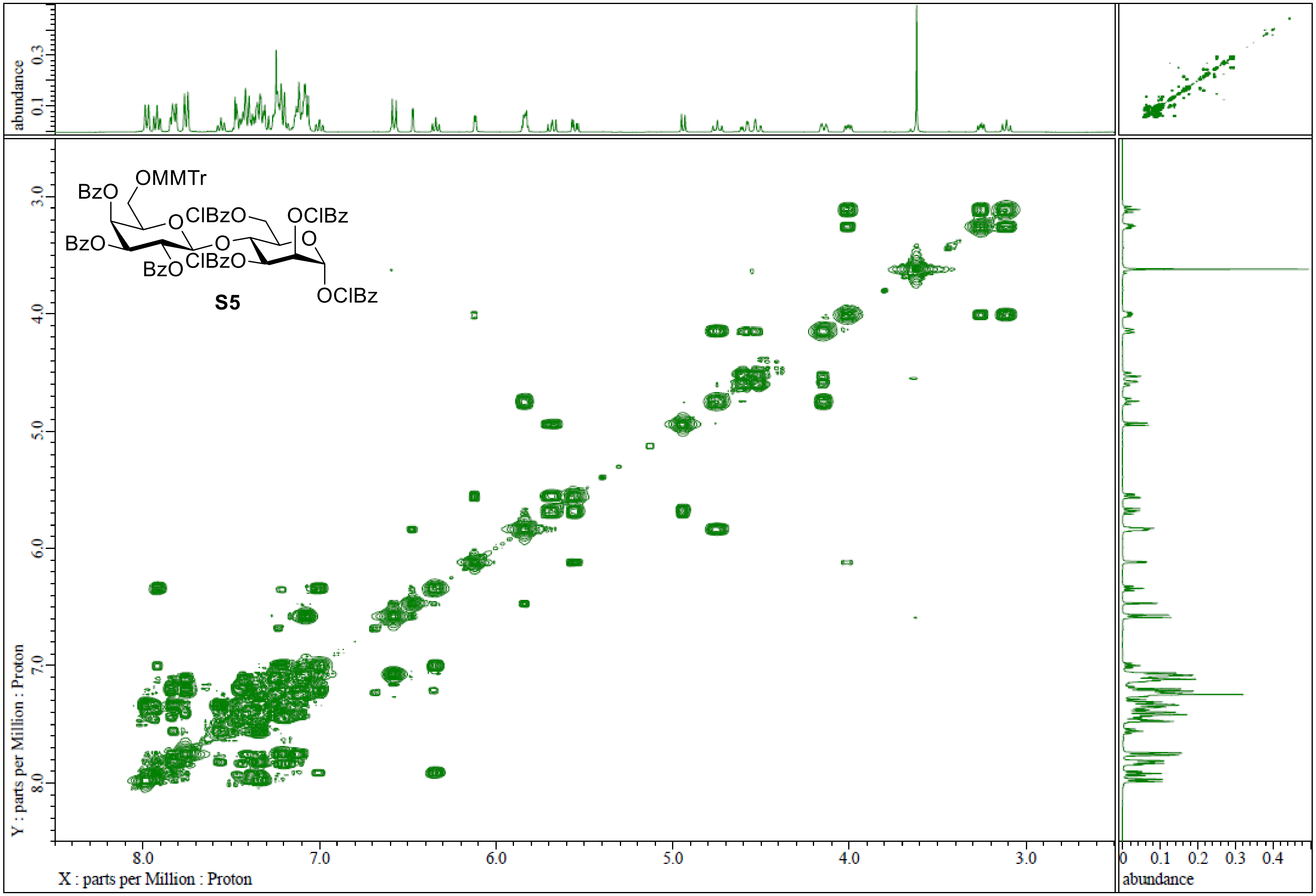

OMMTr
BzO
BzO
BzO
ClBzO
O
O
O
OClBz
O
ClBzO
OClBz
S5
abundance
0.1
0.3
Y : parts per Million : Proton
3.0
4.0
5.0
6.0
7.0
8.0
X : parts per Million : Proton
0
0.1
0.2
0.3
0.4
abundance

HMQC ($CDCl_3$)

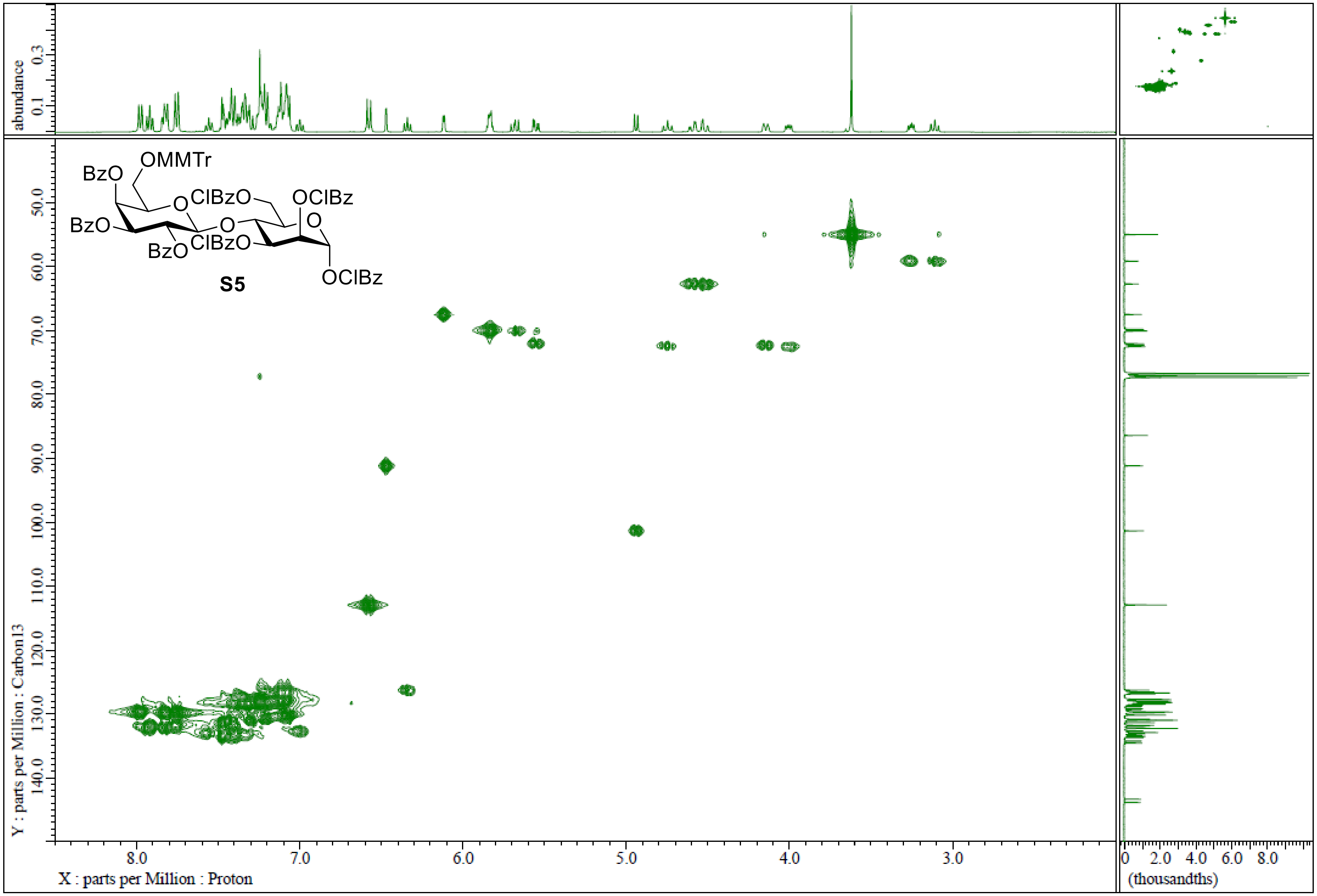

abundance
0.1
0.3
BzO
OMMTr
O
ClBzO
OClBz
BzO
O
O
BzO
ClBzO
OClBz
S5
Y : parts per Million : Carbon13
50.0
60.0
70.0
80.0
90.0
100.0
110.0
120.0
130.0
140.0
8.0
7.0
6.0
5.0
4.0
3.0
X : parts per Million : Proton
0
2.0
4.0
6.0
8.0
(thousandths)

^{1}H NMR (500 MHz, $CDCl_3$)

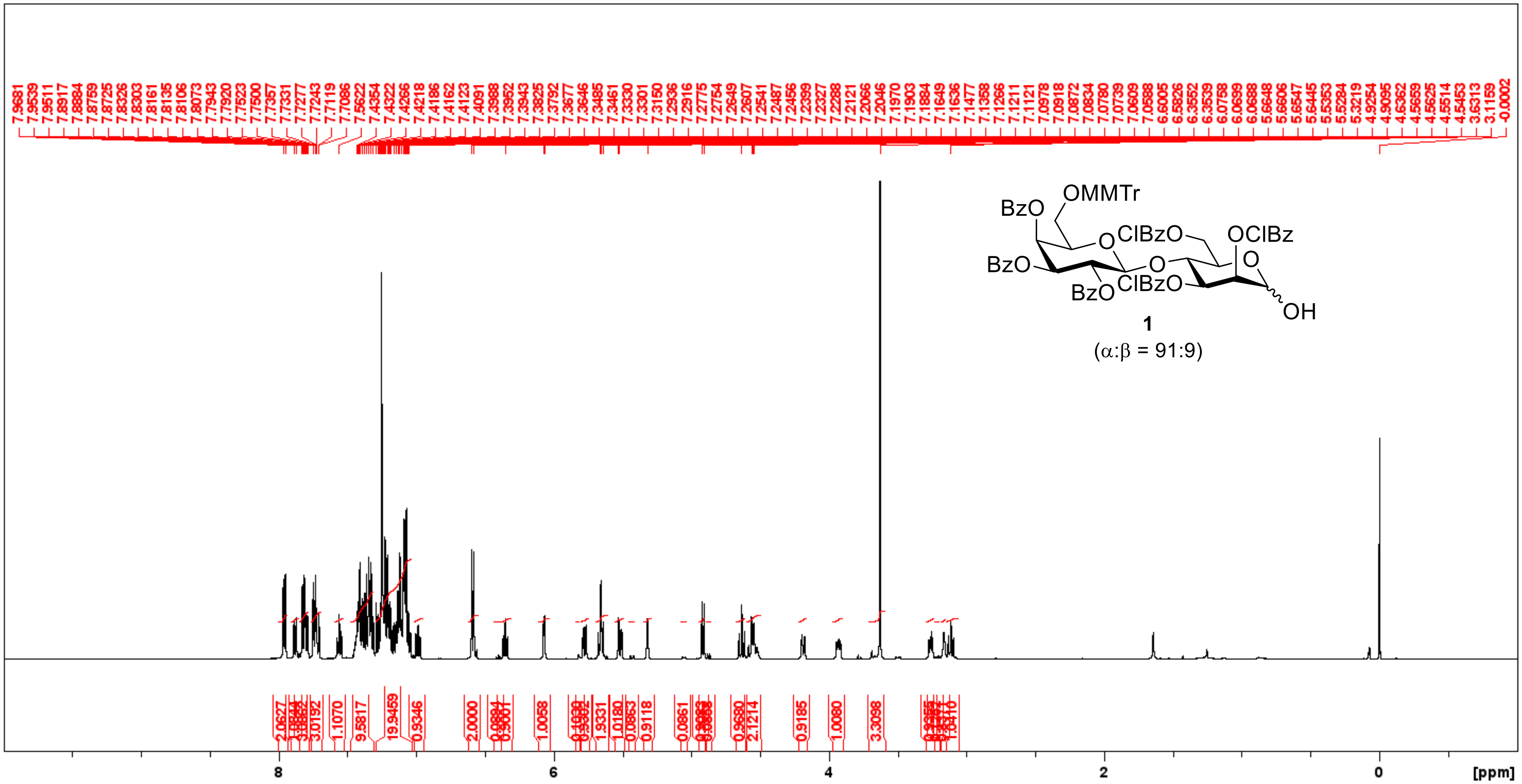

^{1}H NMR (500 MHz, $CDCl_3$, zoom)

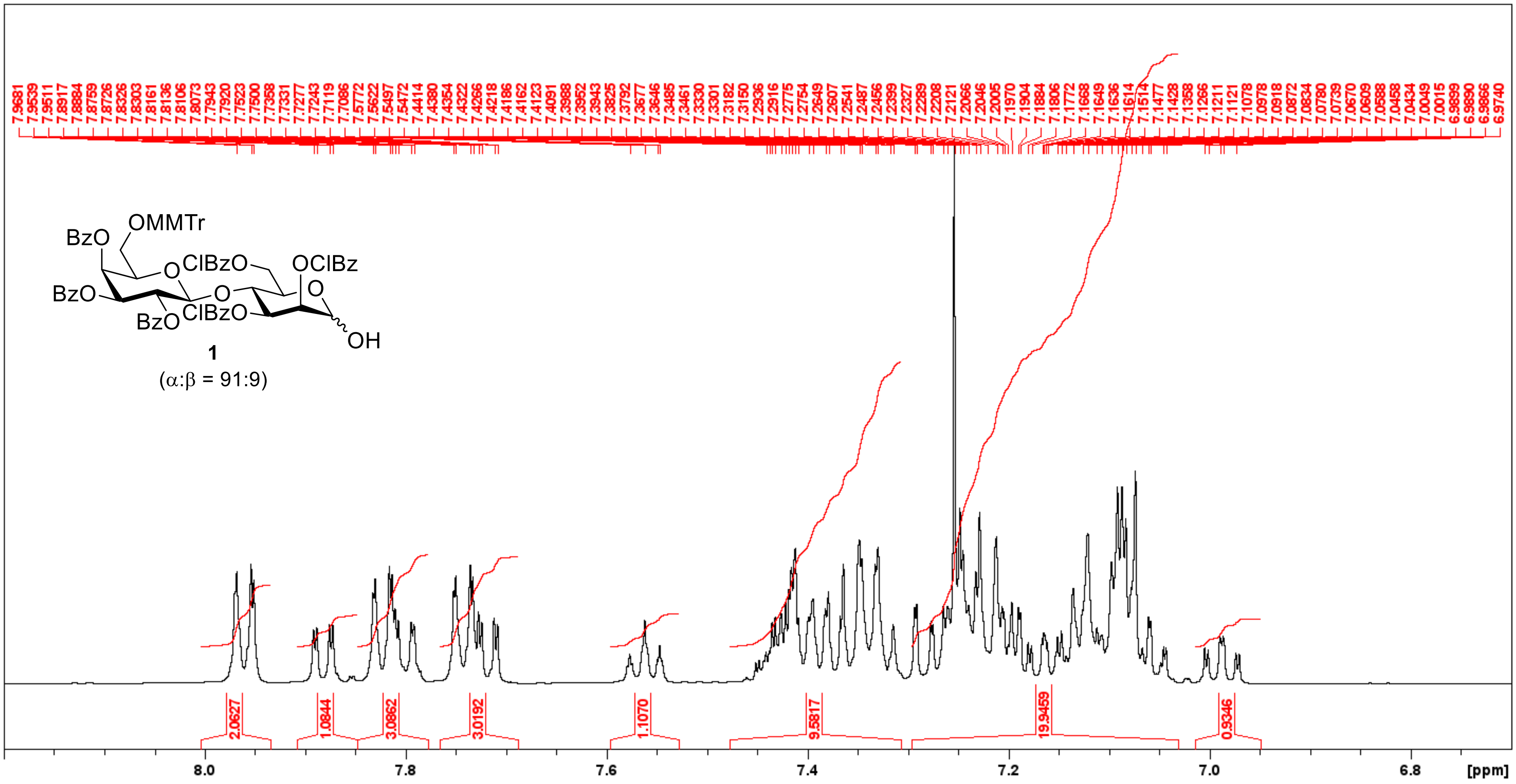

^{1}H NMR (500 MHz, $CDCl_3$, zoom)

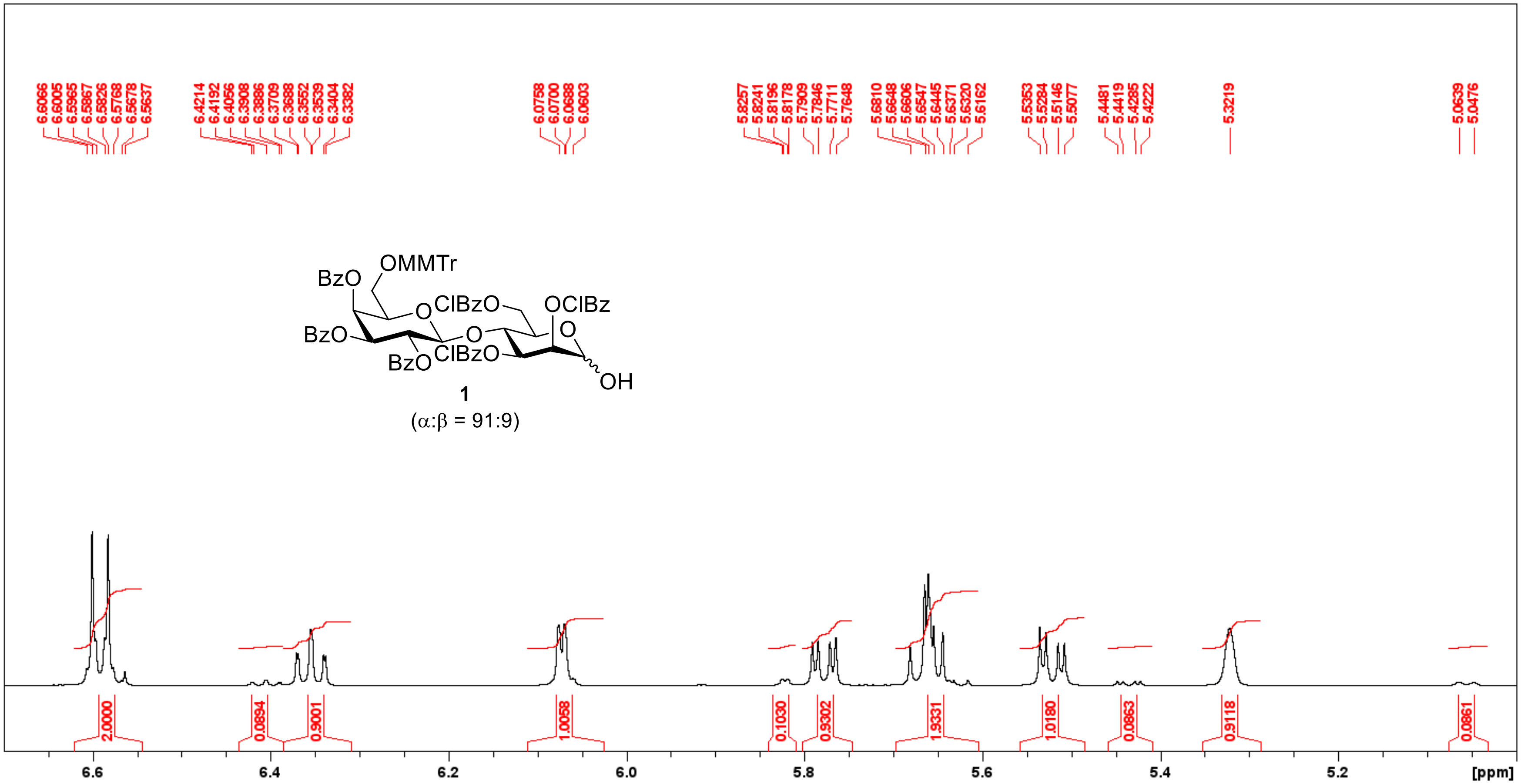

^{1}H NMR (500 MHz, $CDCl_3$, zoom)

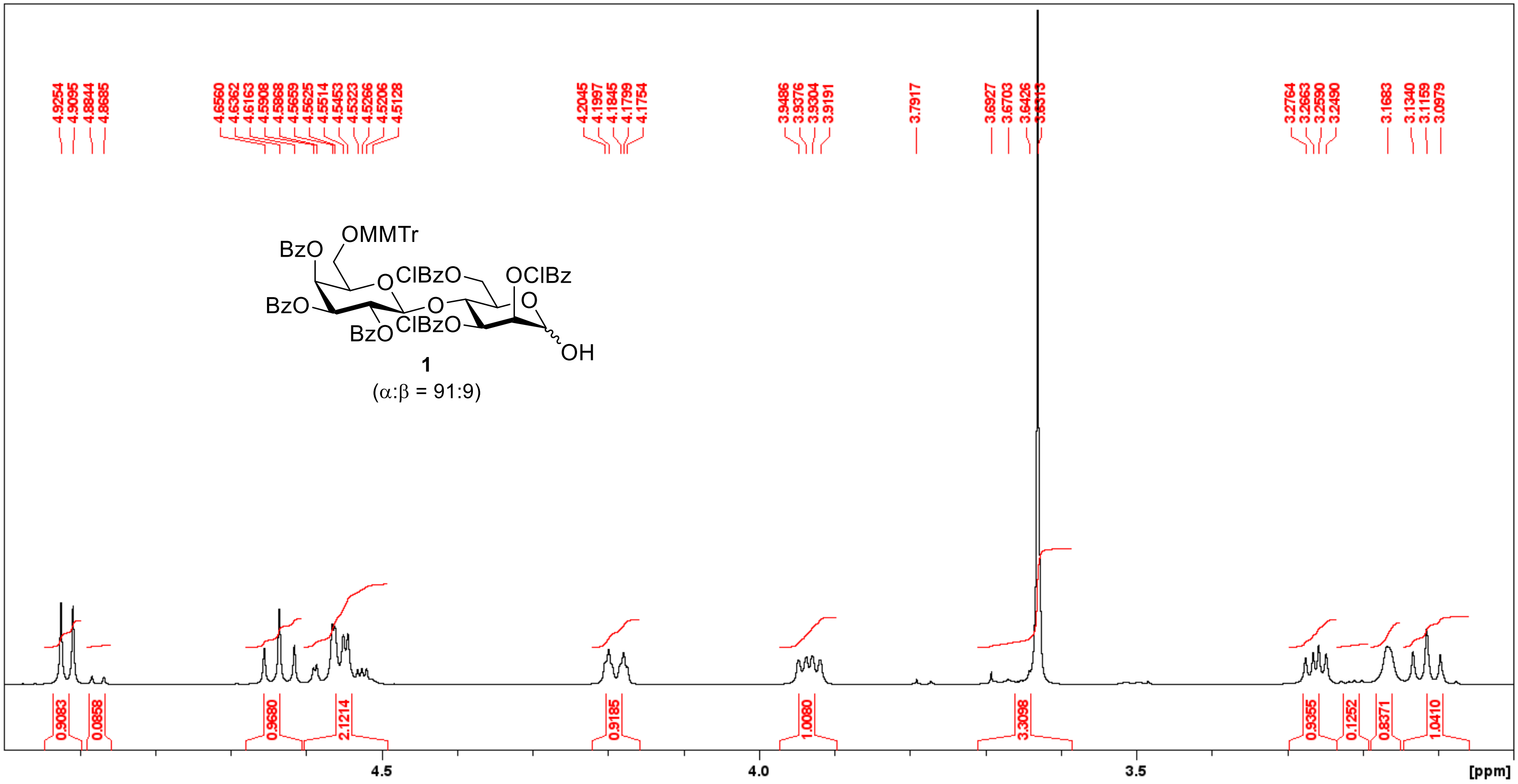

$^{13}C\{^1H\}$ NMR (126 MHz, $CDCl_3$)

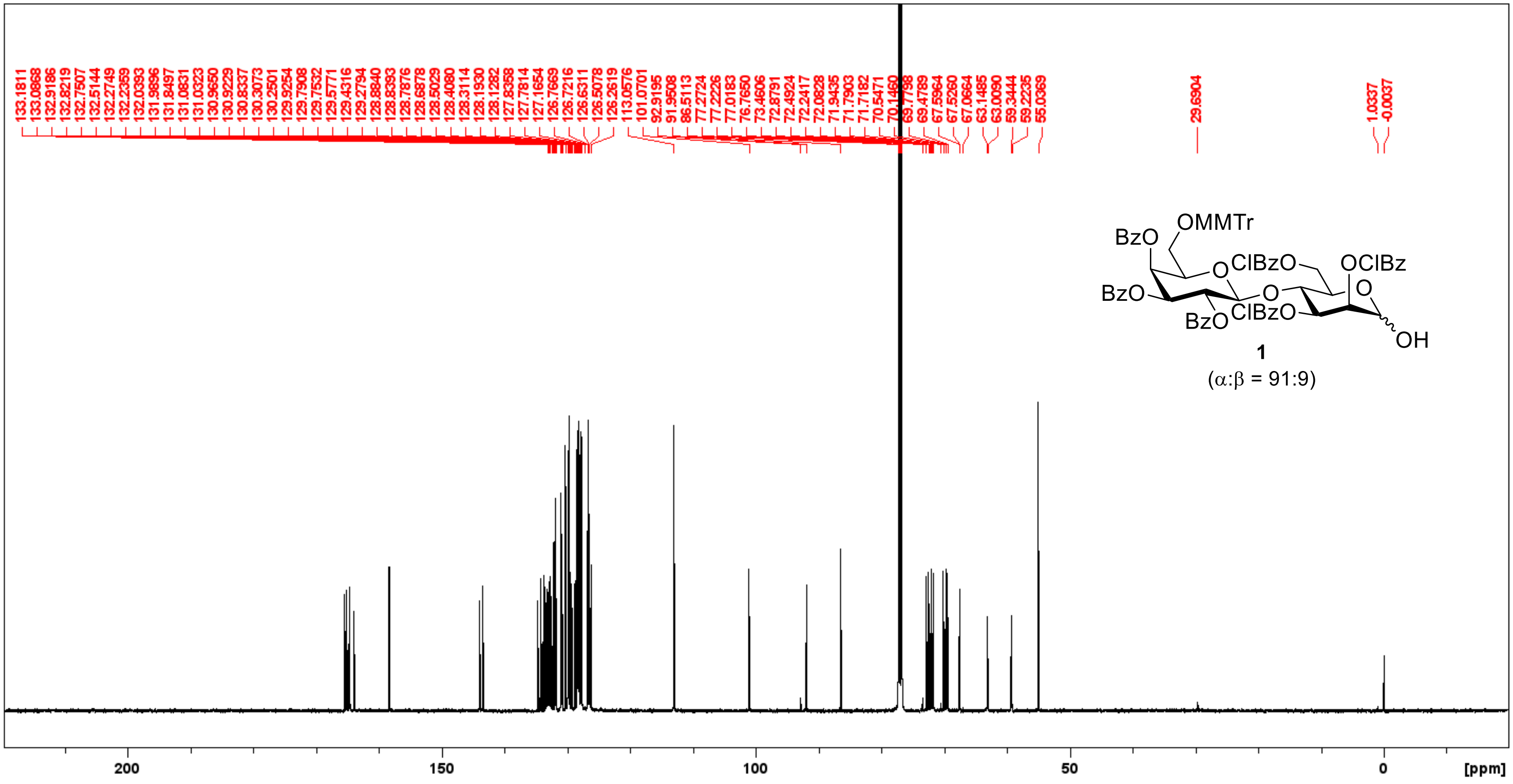

COSY ($CDCl_3$)

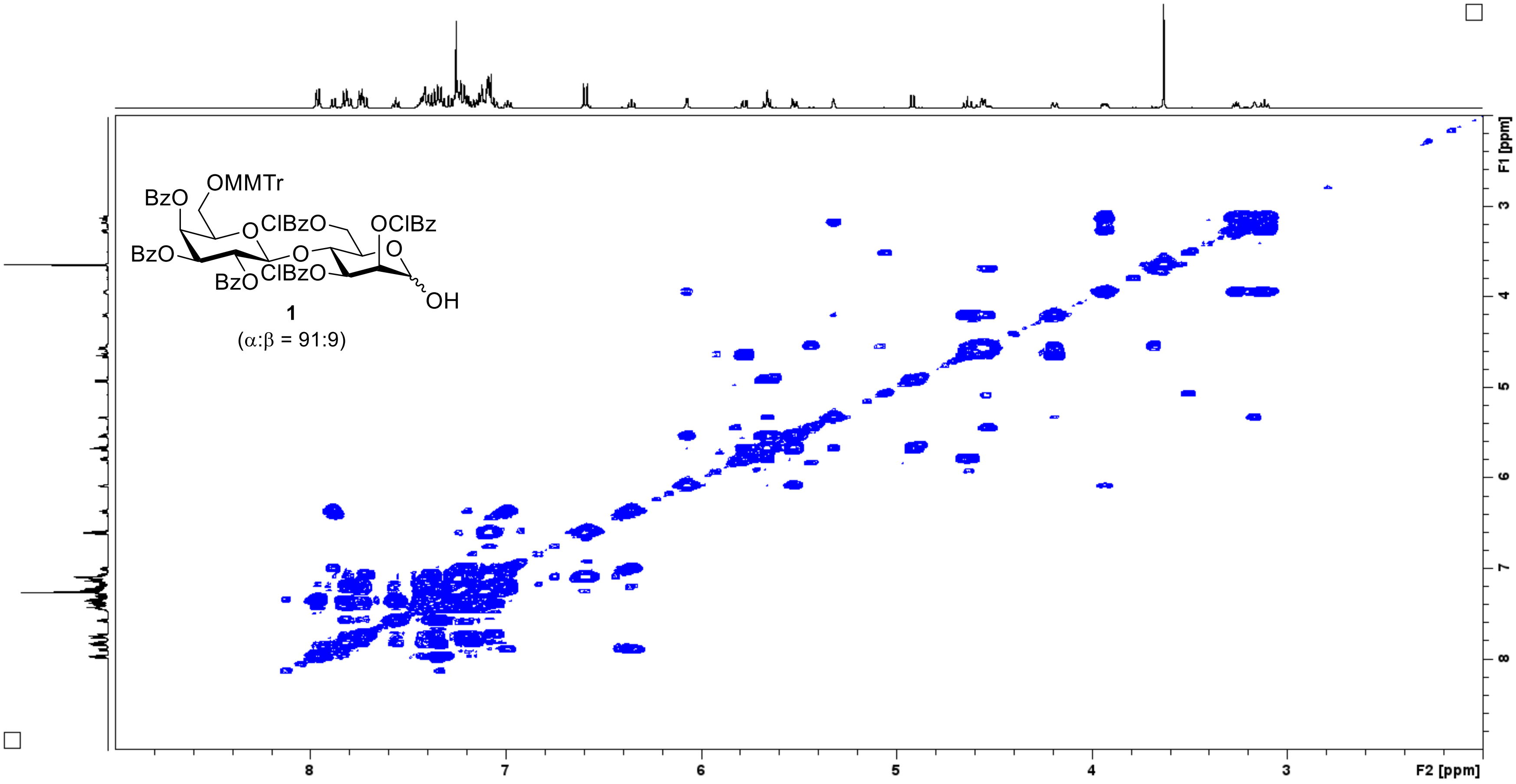
OMMTr
BzO
BzO
O
ClBzO
OClBz
O
O
BzO
ClBzO
OH
1
(α:β = 91:9)
F1 [ppm]
F2 [ppm]

HSQC (CDCl3)

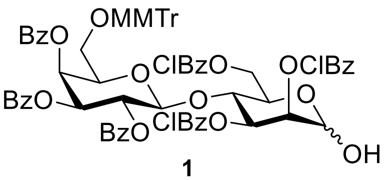

1

(α:β = 91:9)

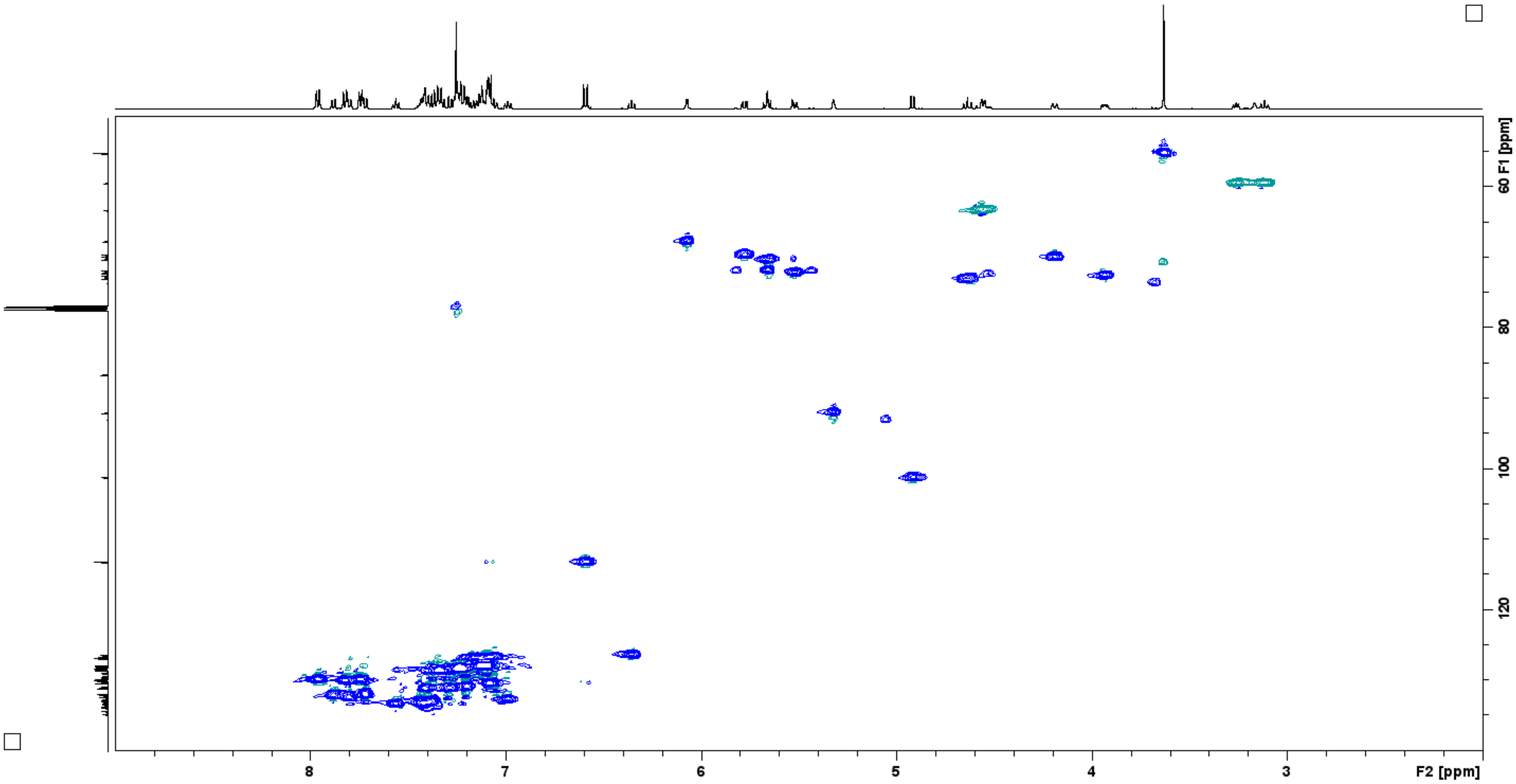

HMBC ($CDCl_3$)

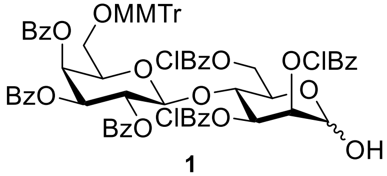

1

(α:β = 91:9)

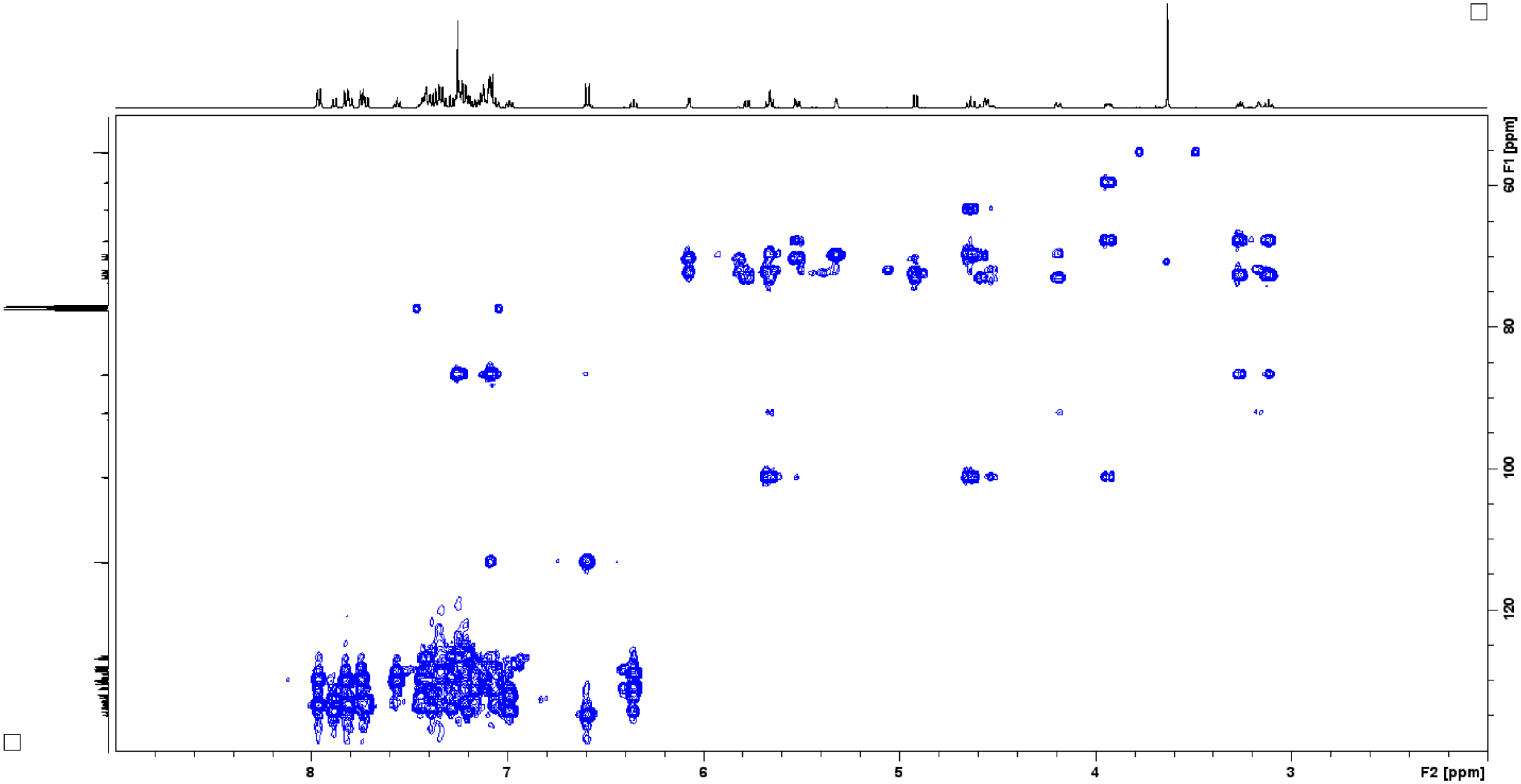

^{1}H NMR (500 MHz, CDCl$_3$)

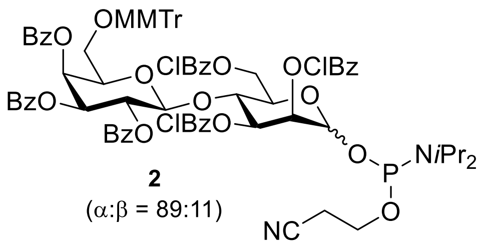

2

(α:β = 89:11)

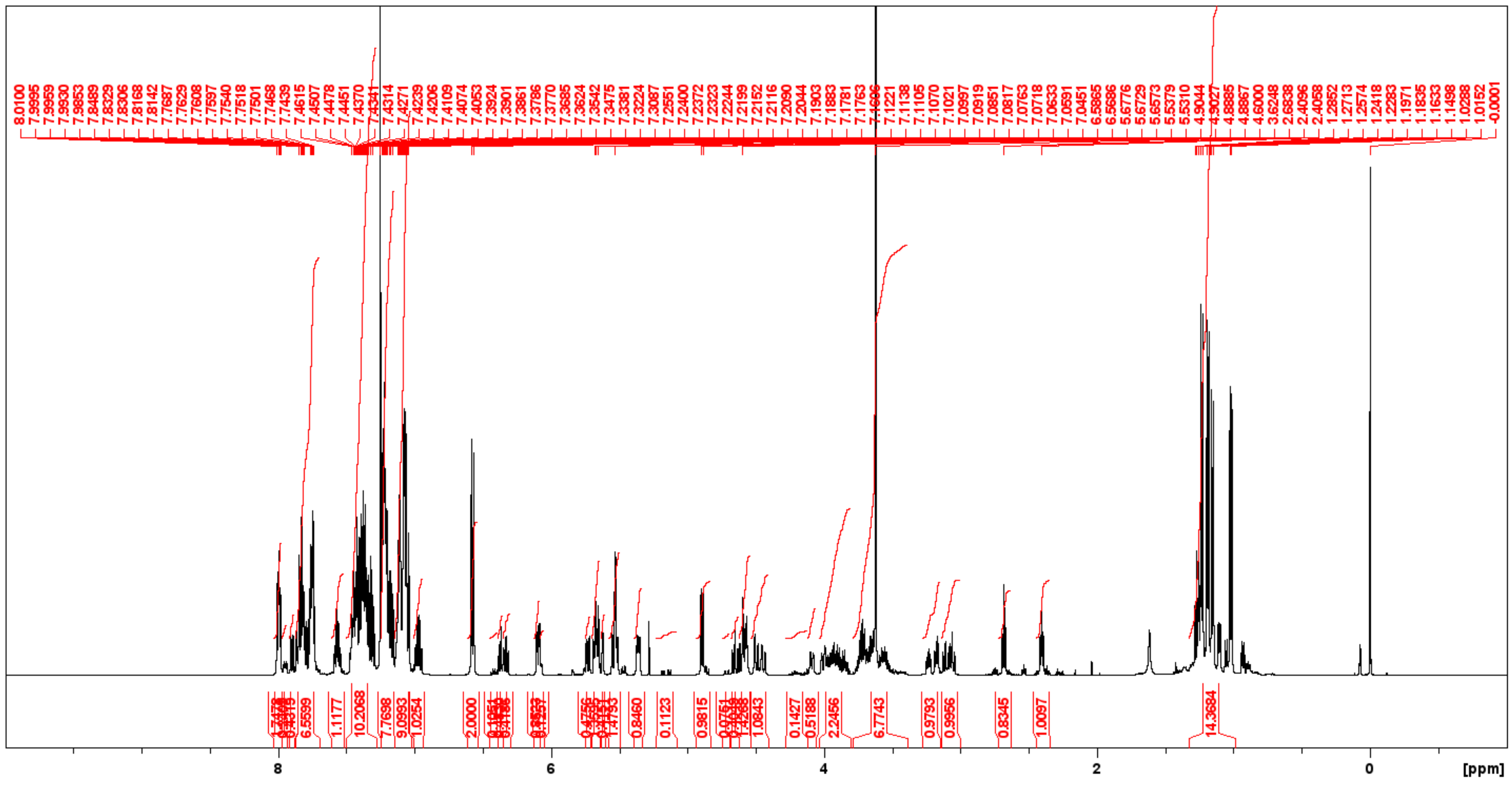

^{1}H NMR (500 MHz, $CDCl_3$, zoom)

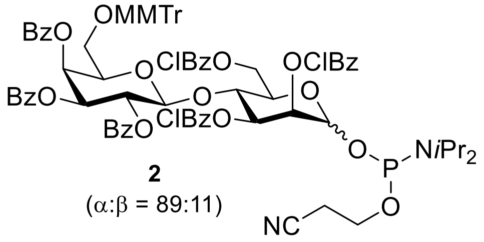

2
(α:β = 89:11)

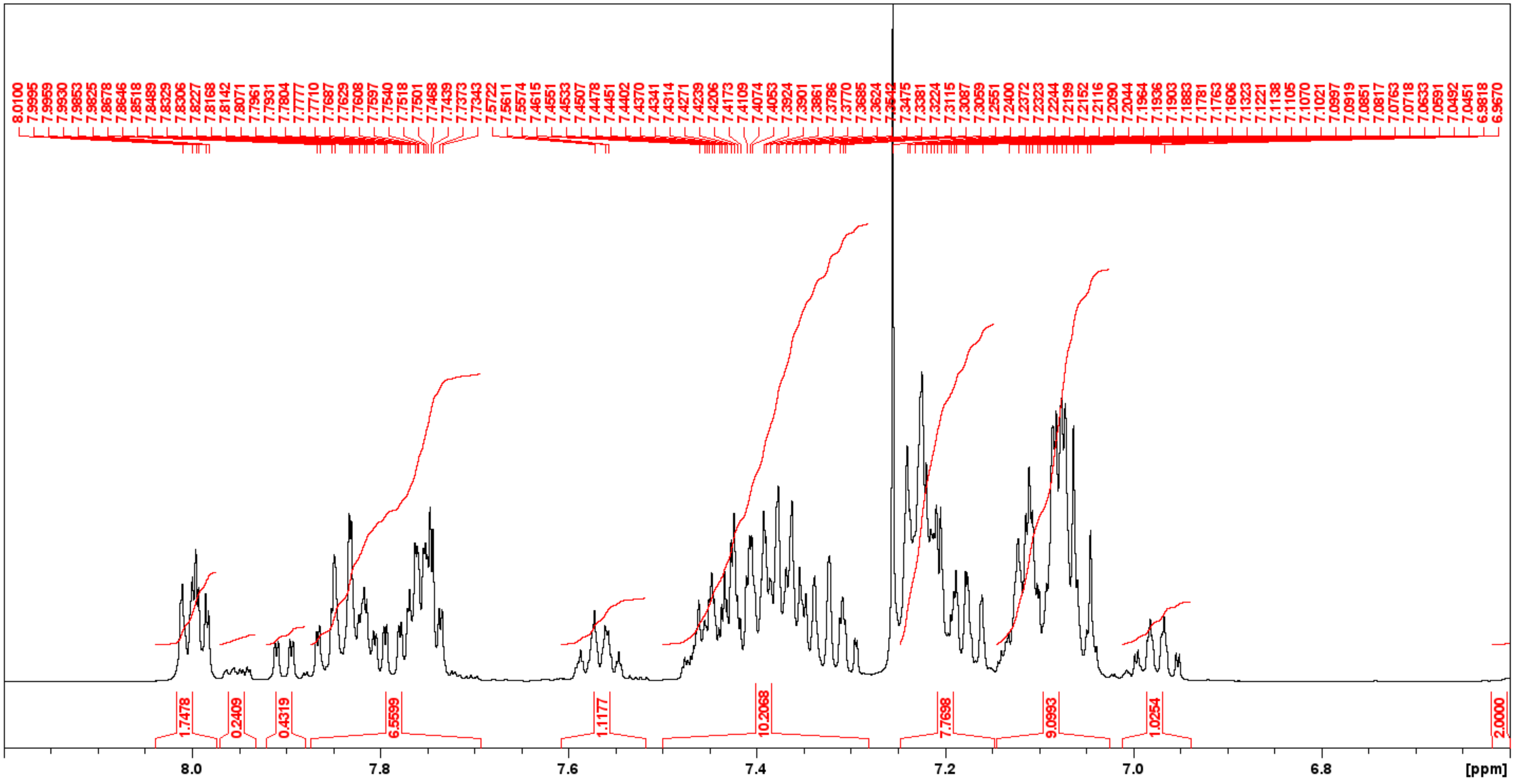

^{1}H NMR (500 MHz, CDCl3, zoom)

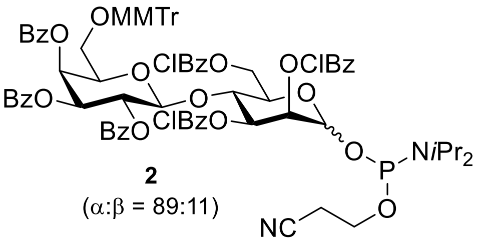

2

(α:β = 89:11)

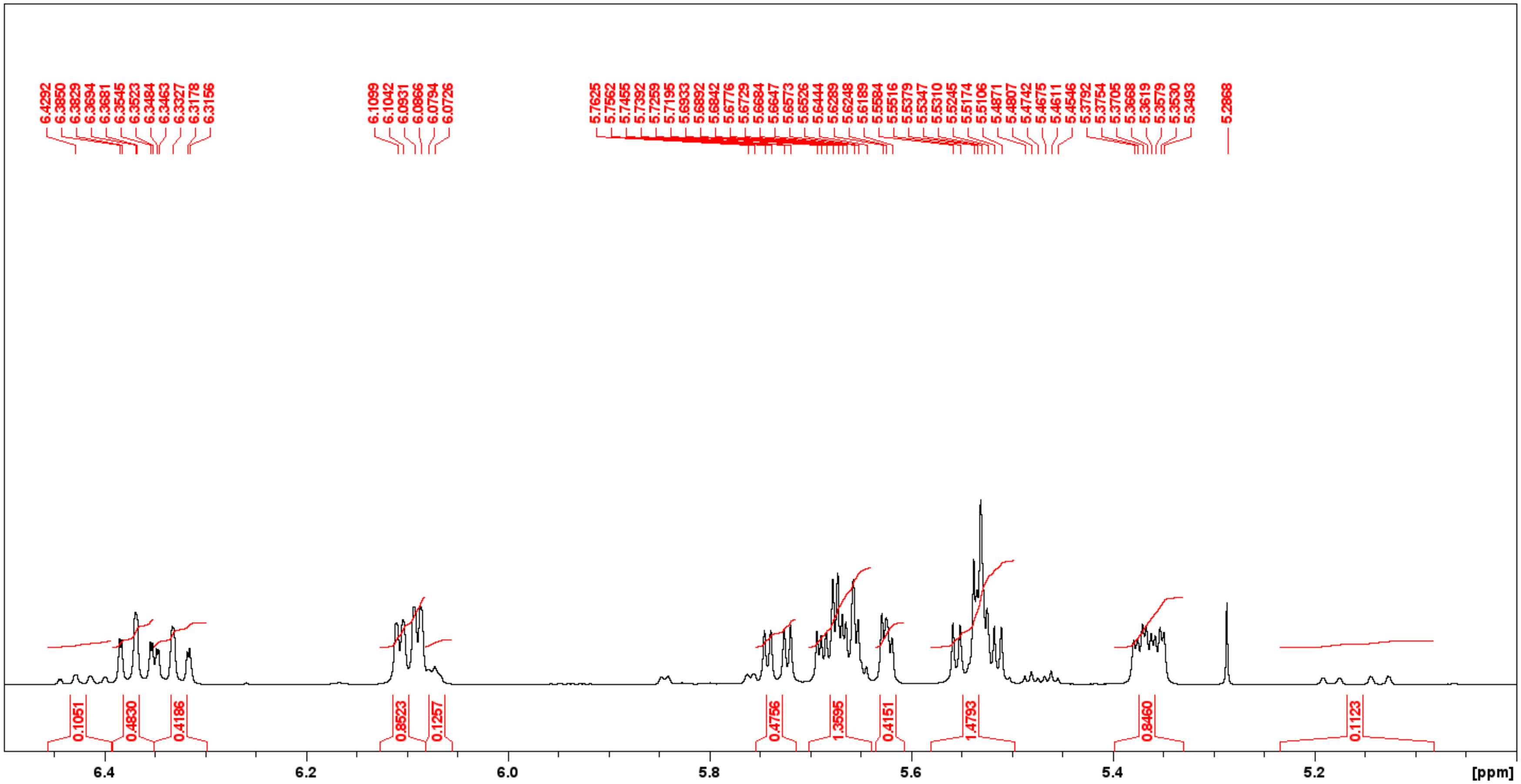

^{1}H NMR (500 MHz, CDCl$_3$, zoom)

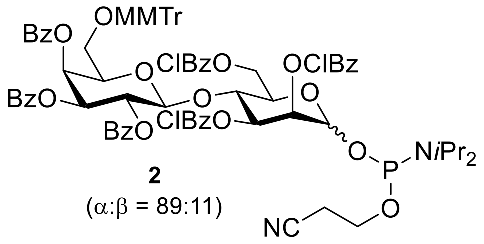

2

(α:β = 89:11)

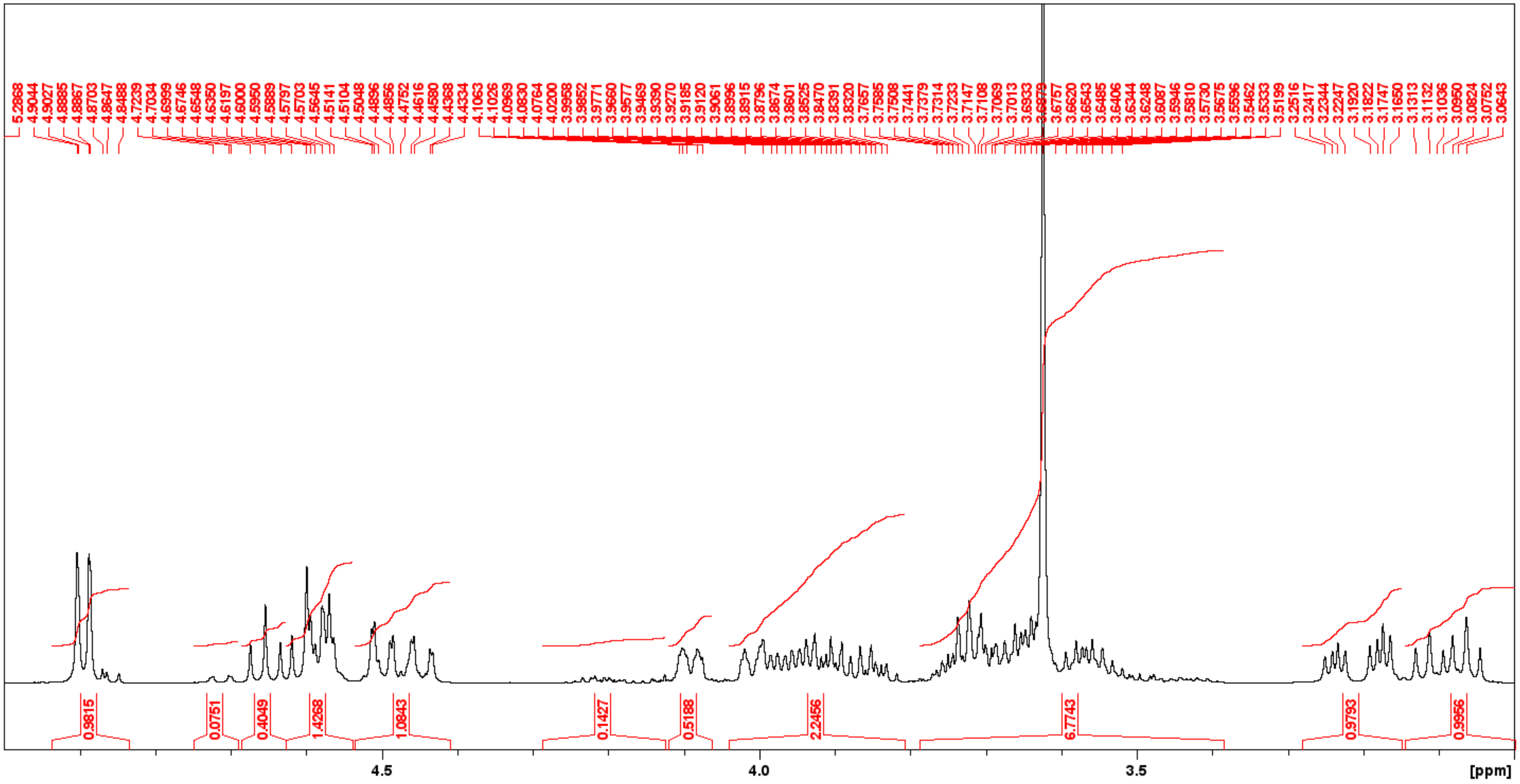

$^{13}C\ \{^1H\}$ NMR (126 MHz, $CDCl_3$)

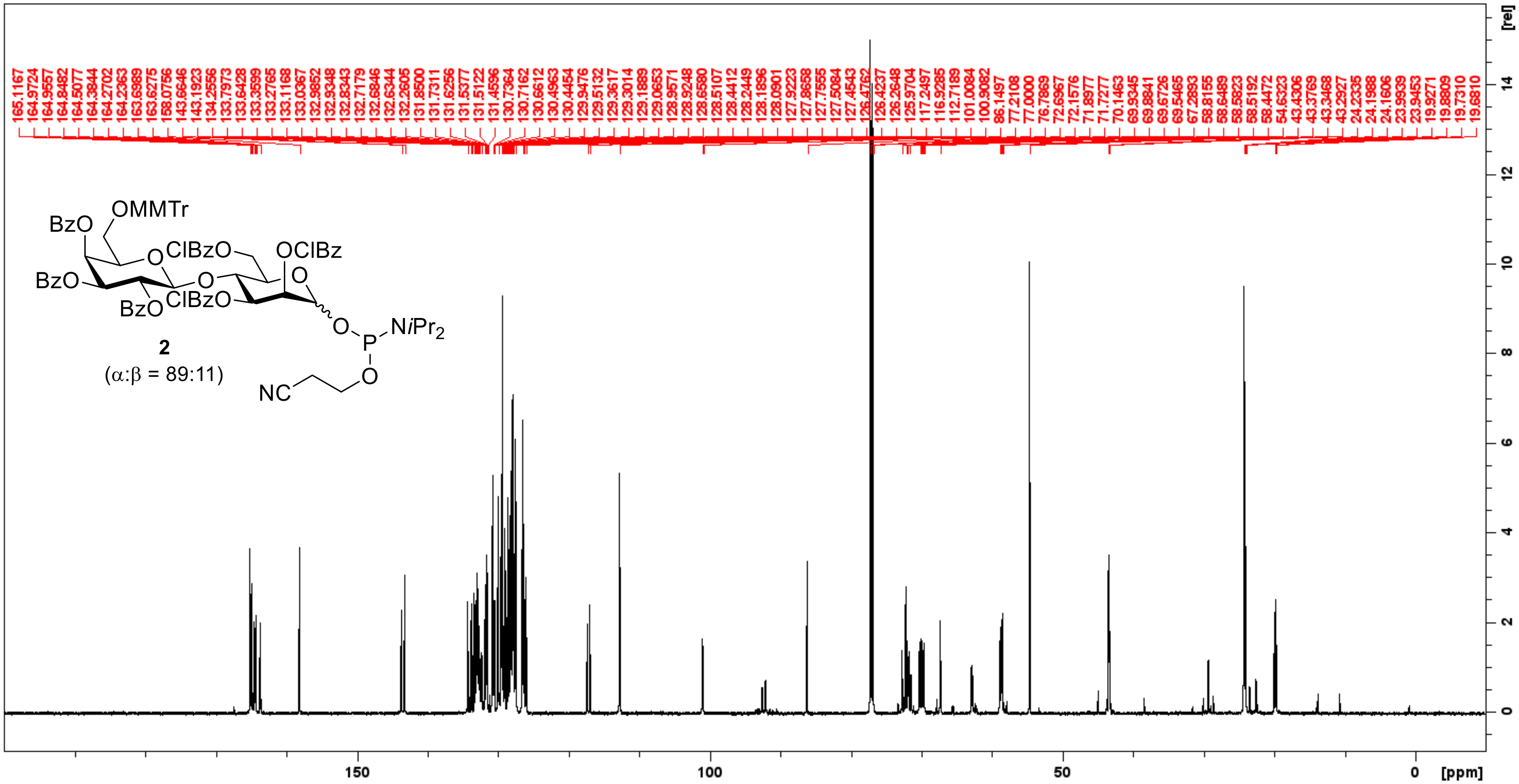

^{31}P NMR (202 MHz, CDCl$_3$)

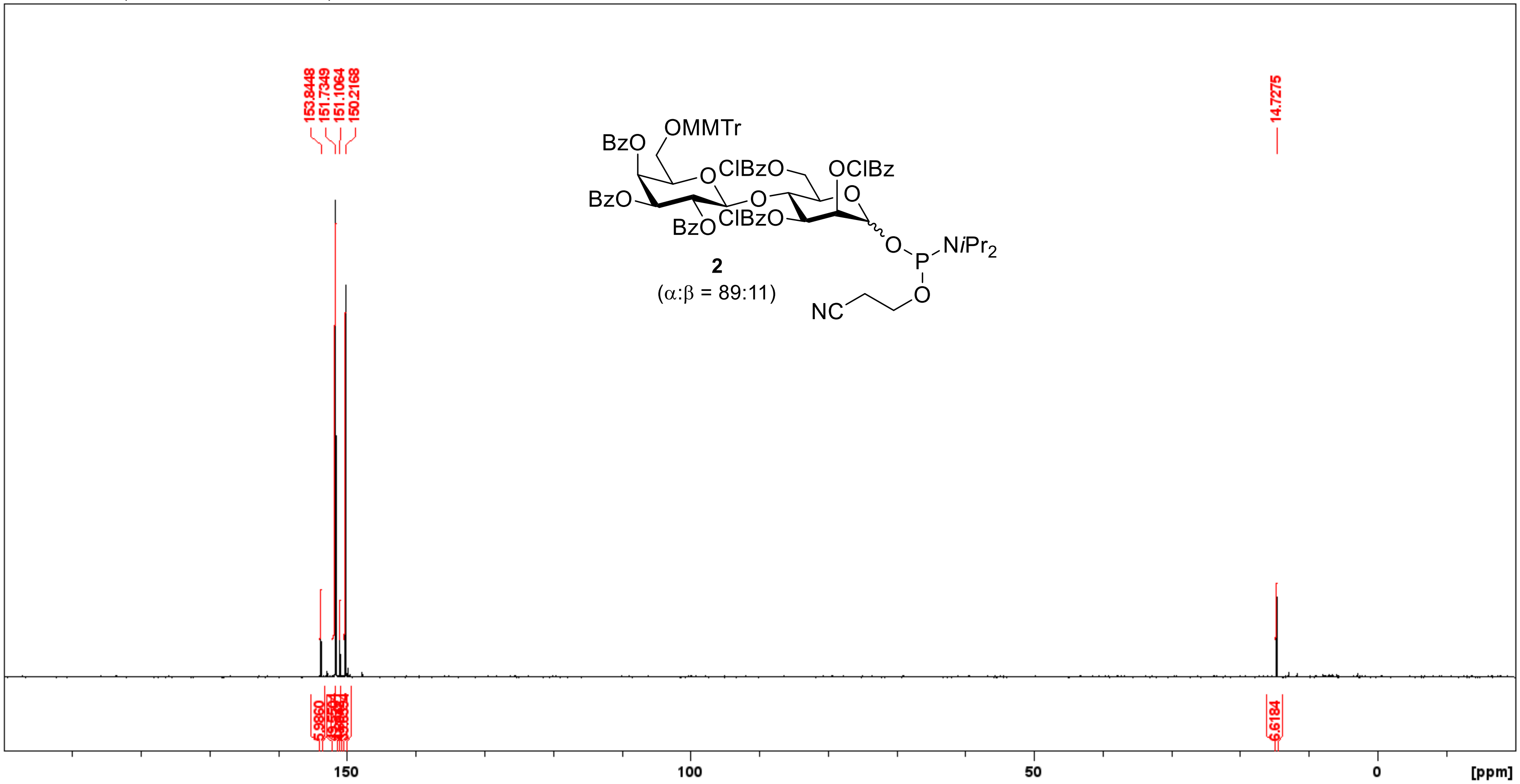

^{31}P NMR (202 MHz, $CDCl_3$, zoom)

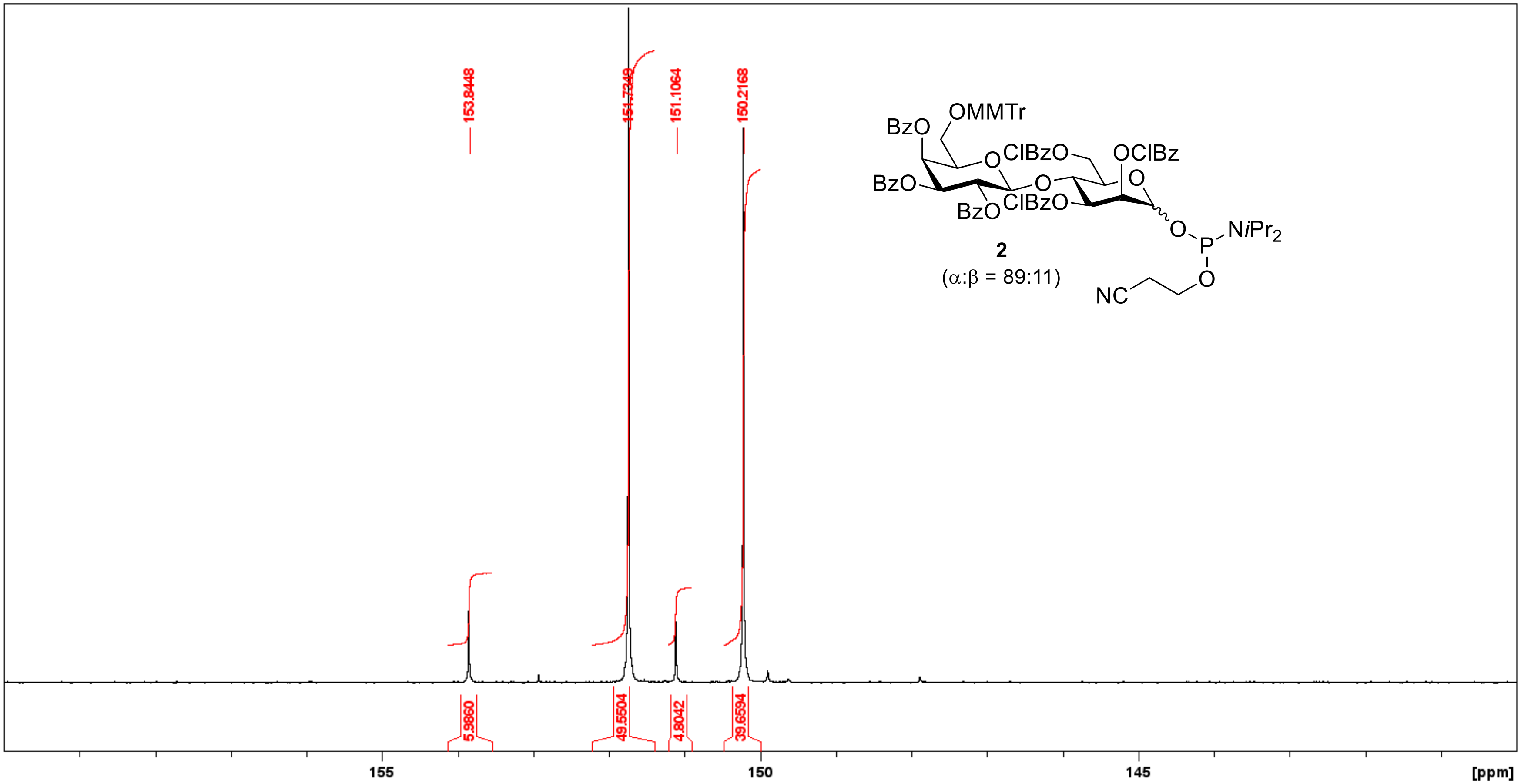

COSY (CDCl$_3$)

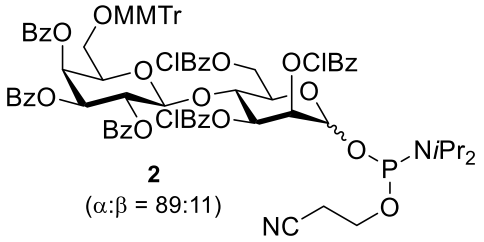

2

(α:β = 89:11)

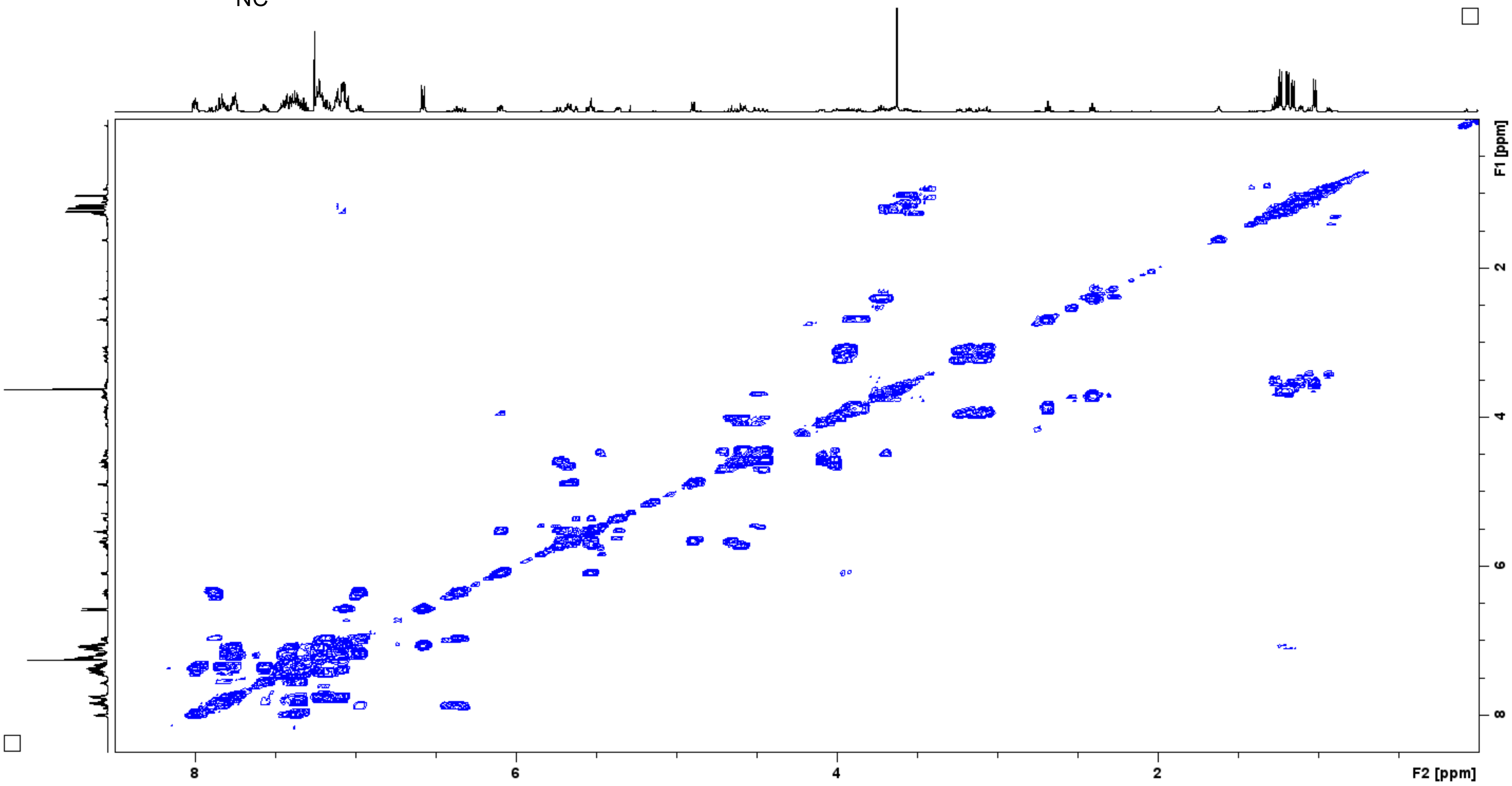

HSQC ($CDCl_3$)

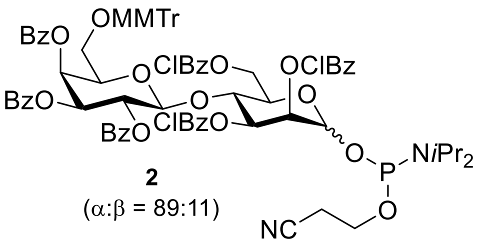

2
(α:β = 89:11)

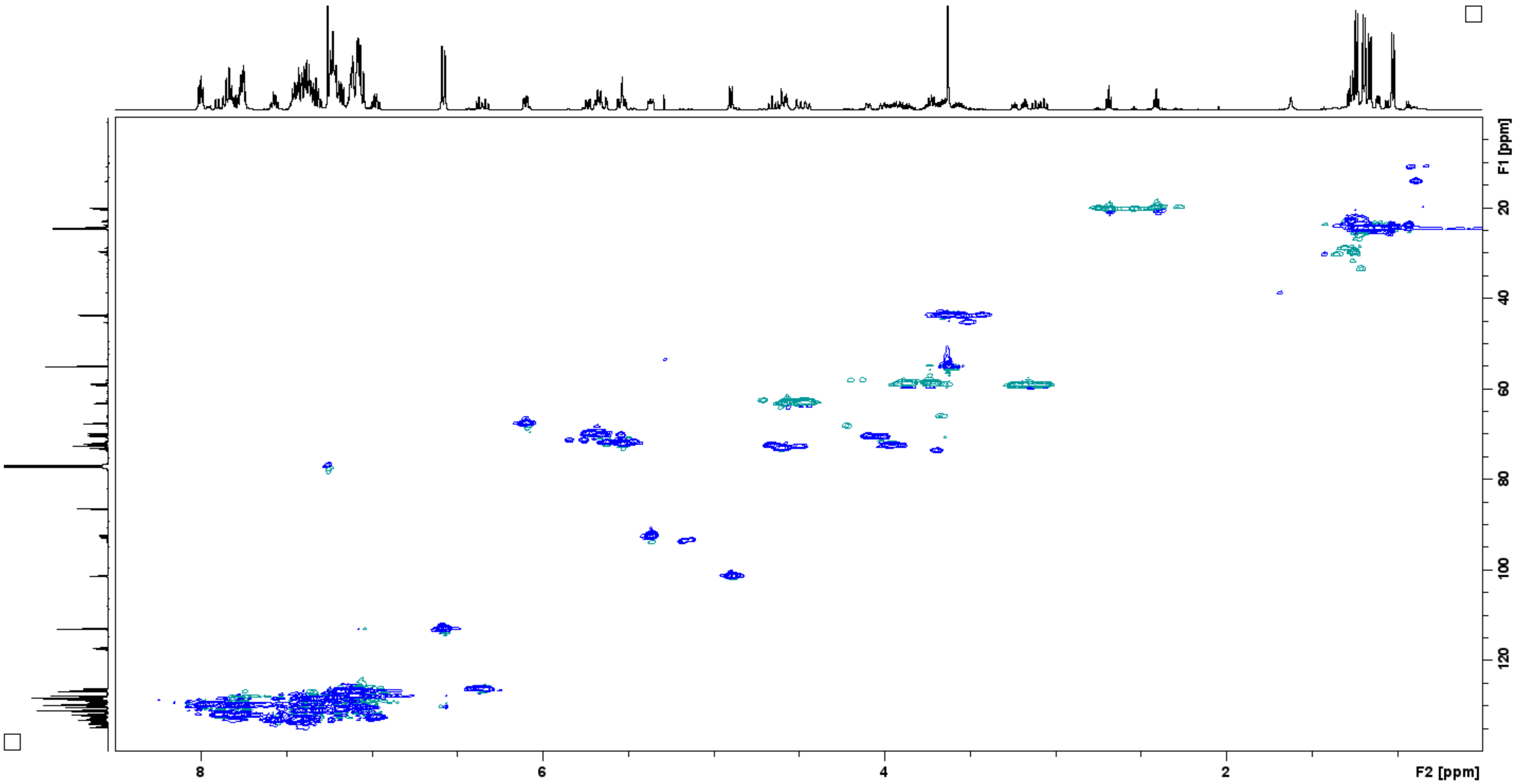

HMBC (CDCl3)

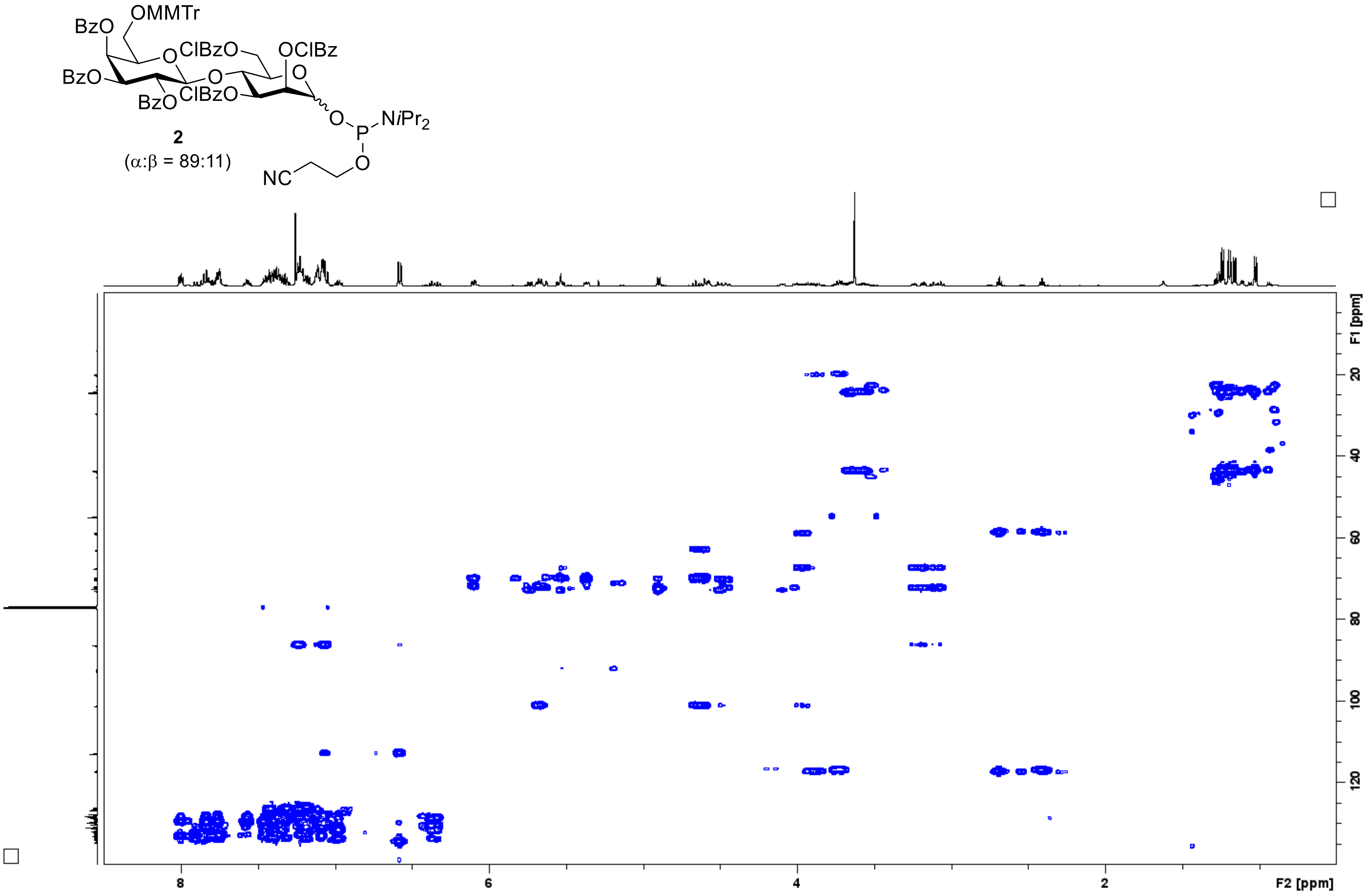

OMMTr
BzO
ClBzO
OClBz
BzO
BzO
ClBzO
O
P
NiPr2
NC
2
(α:β = 89:11)
F1 [ppm]
F2 [ppm]
20
40
60
80
100
120
8
6
4
2

^{1}H NMR (600 MHz, D_2O)

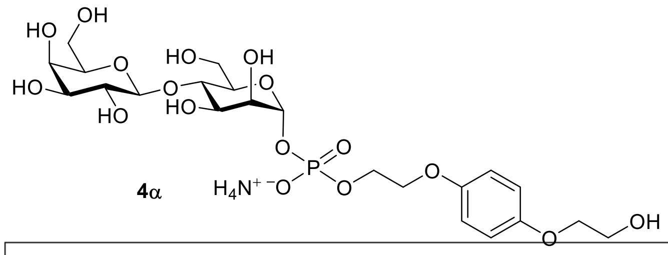

4α

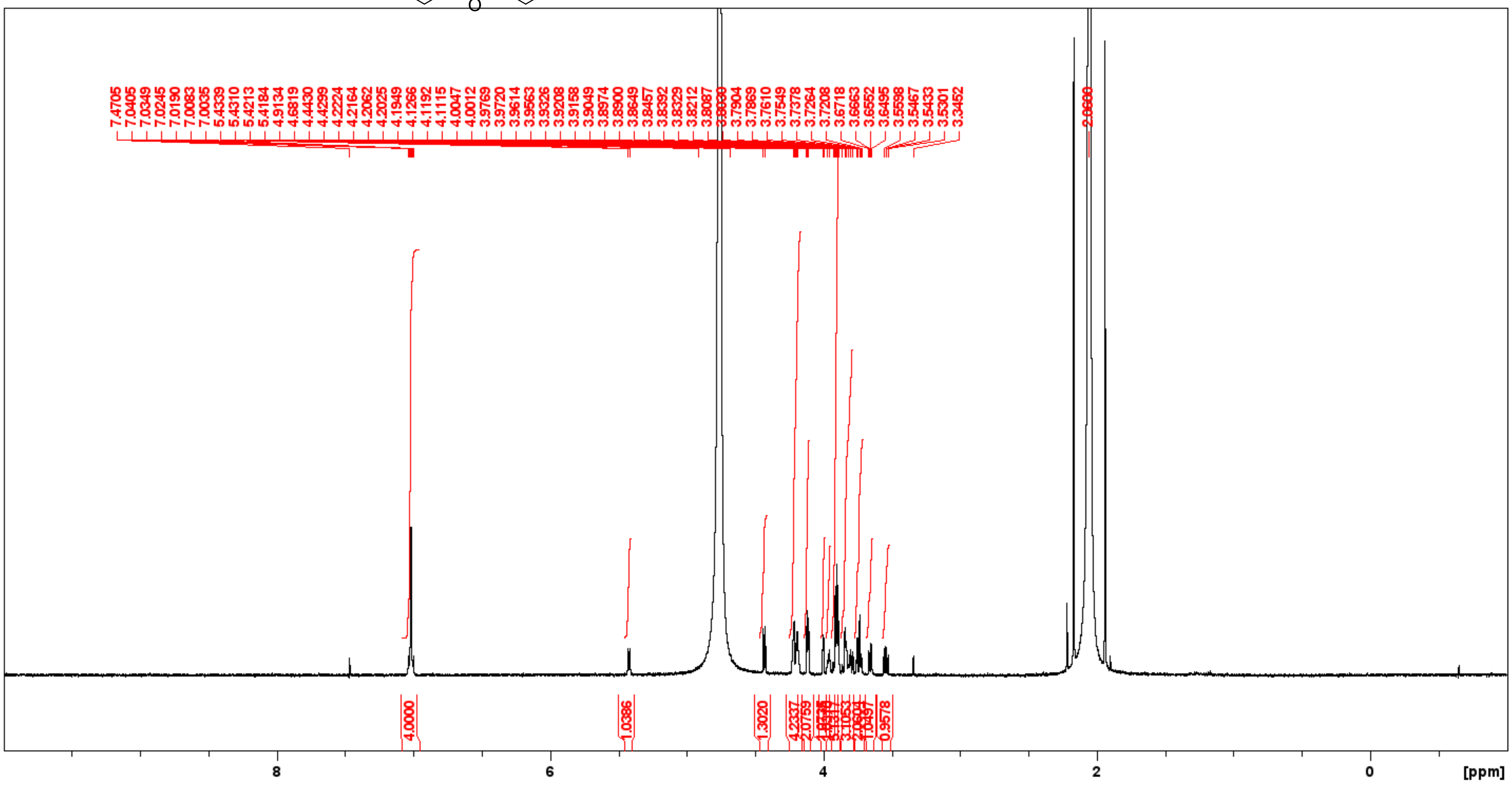

^{1}H NMR (600 MHz, D_2O)

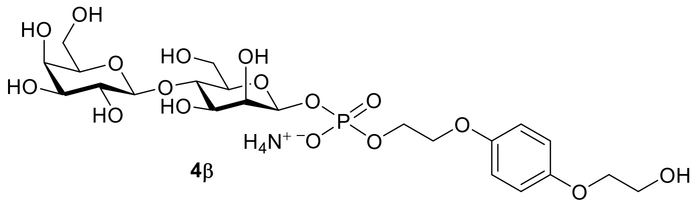

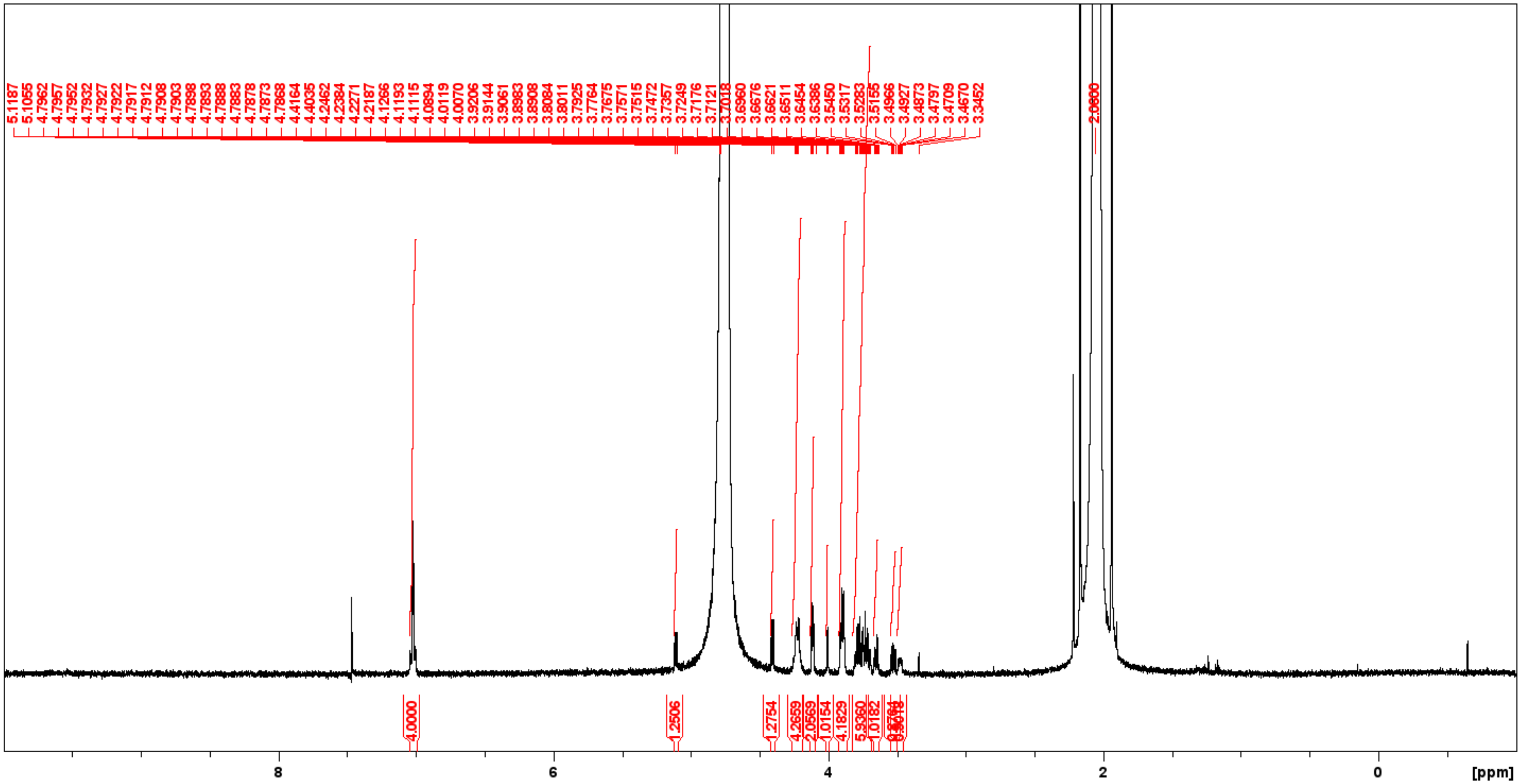

^{1}H NMR (500 MHz, D2O)

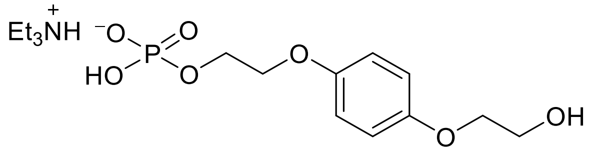

6

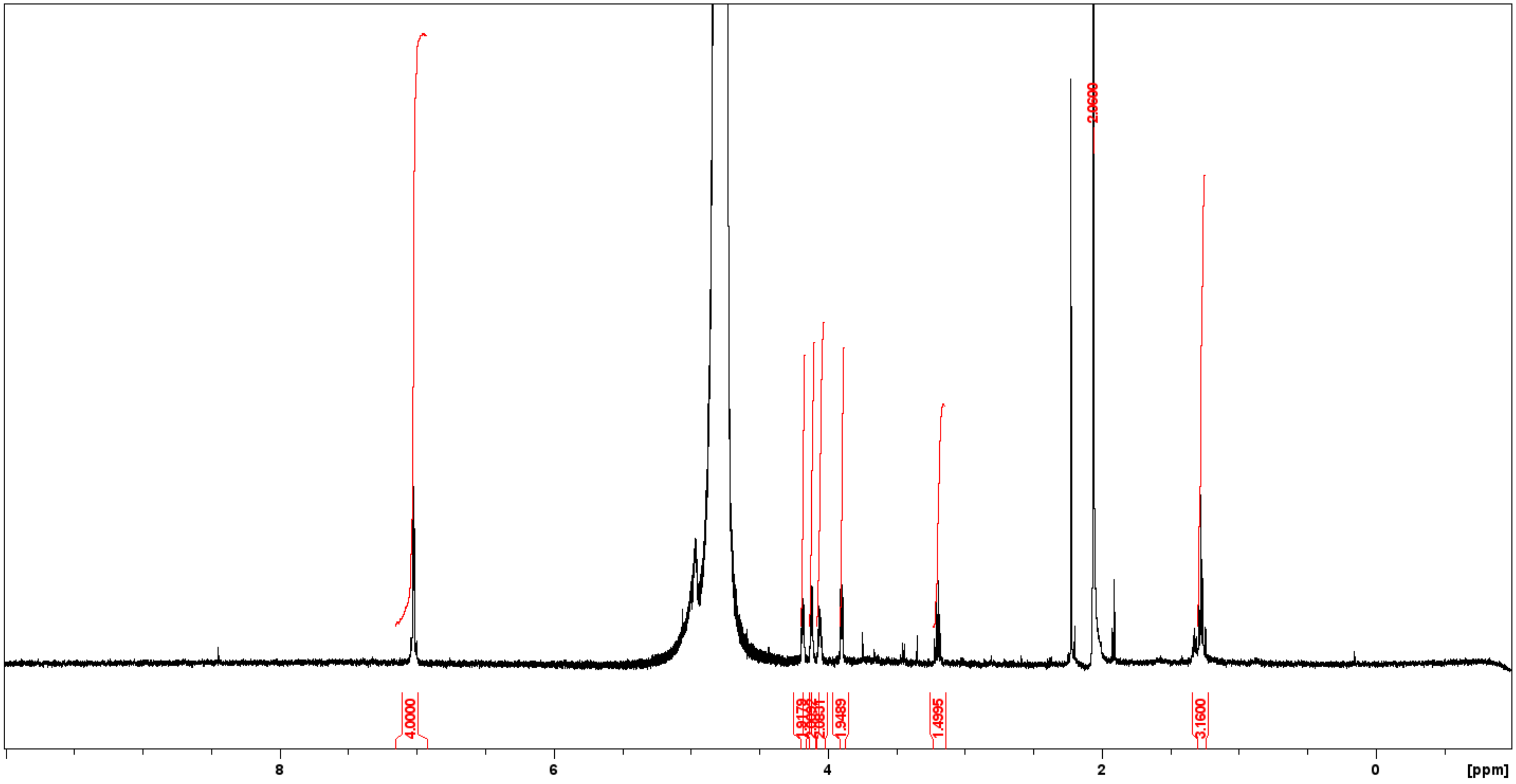

^{1}H NMR (600 MHz, D_2O)

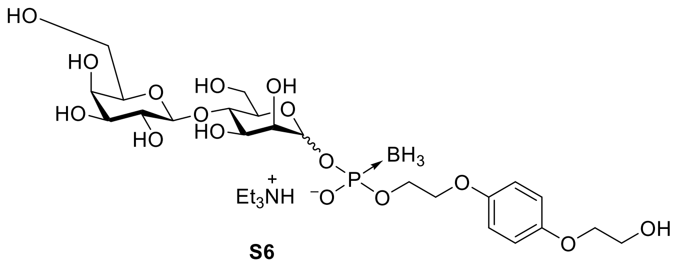

S6

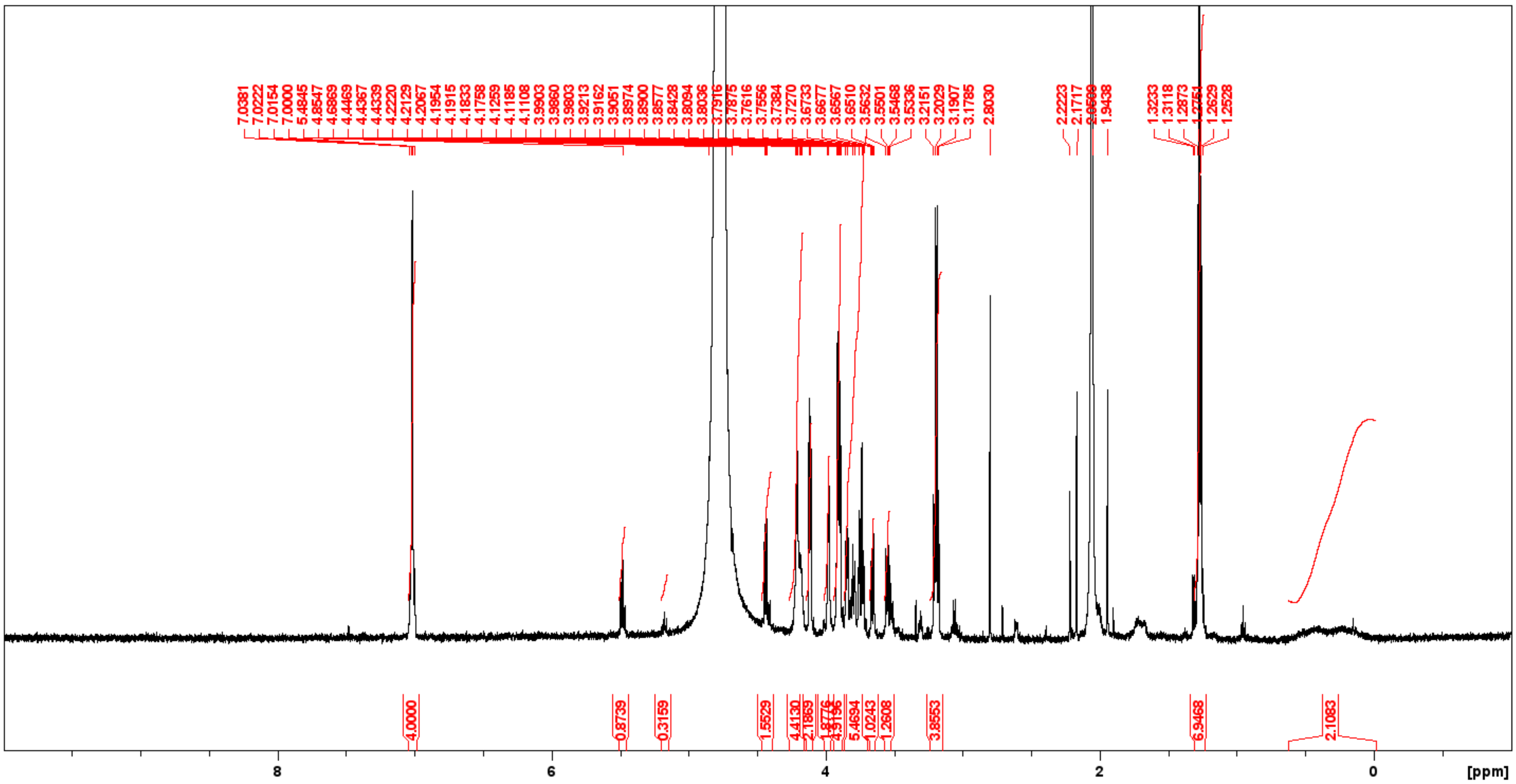

^{1}H NMR (600 MHz, D_2O)

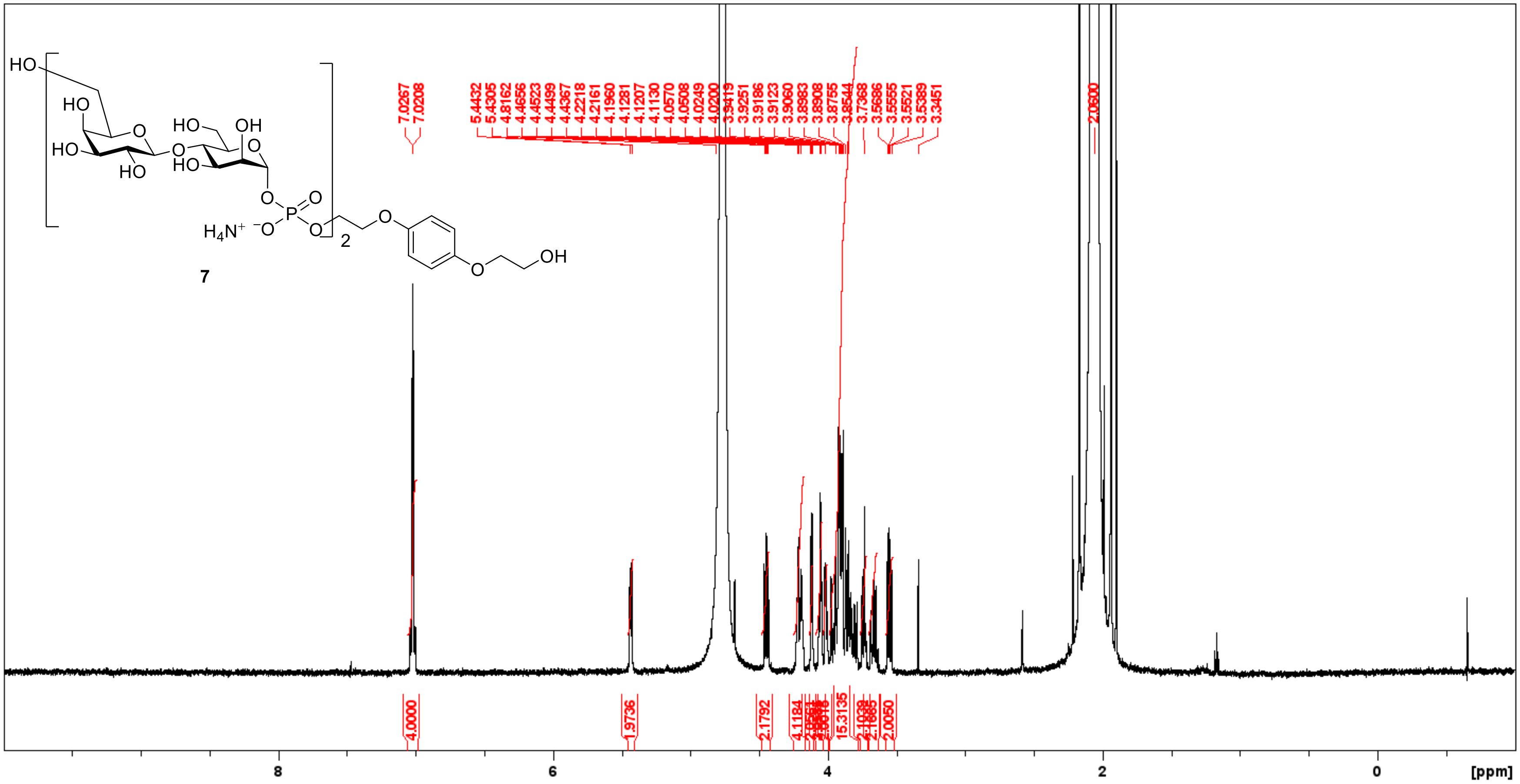

^{1}H NMR (600 MHz, D_2O)

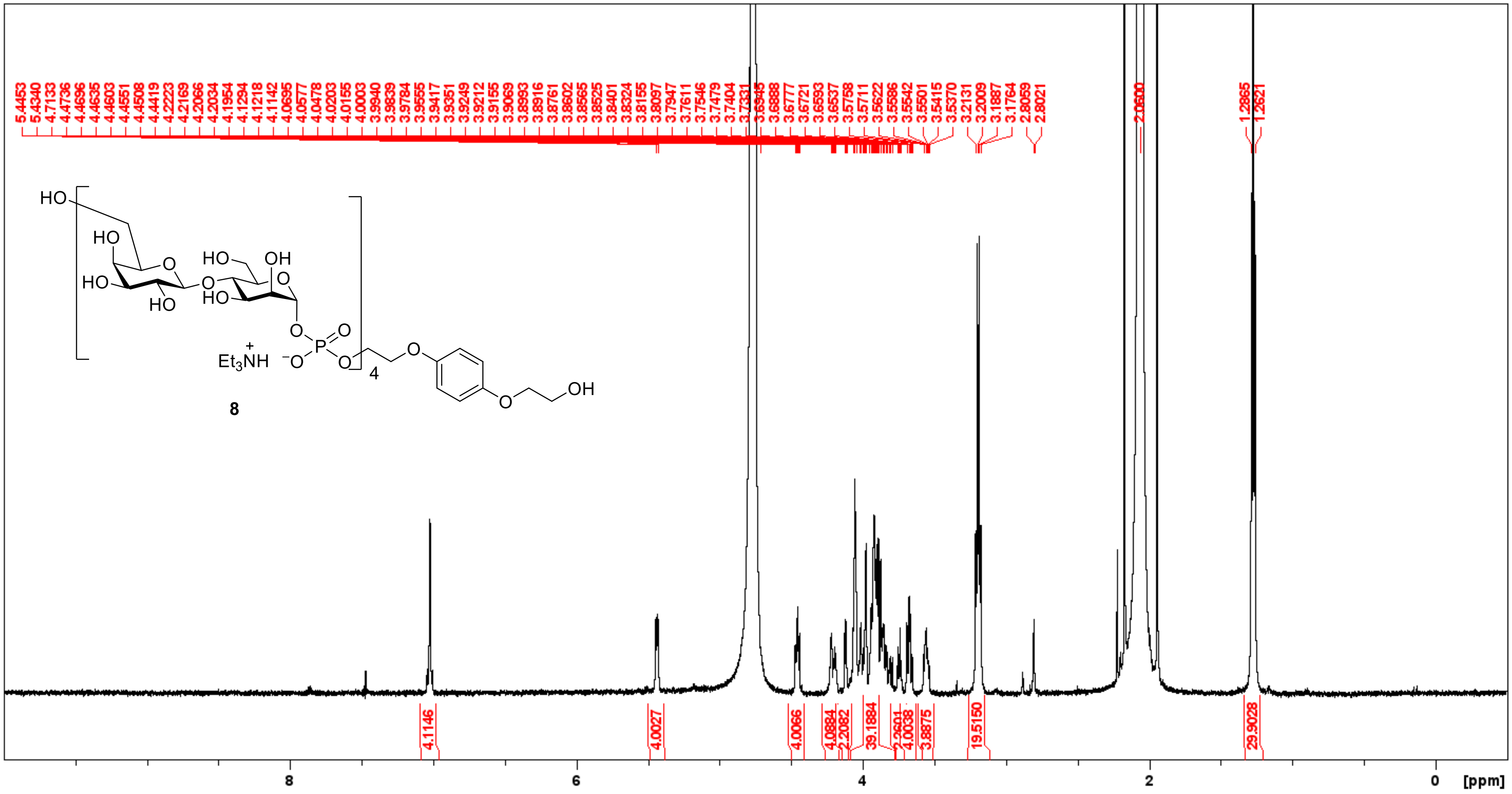

^{1}H NMR (600 MHz, D_2O)

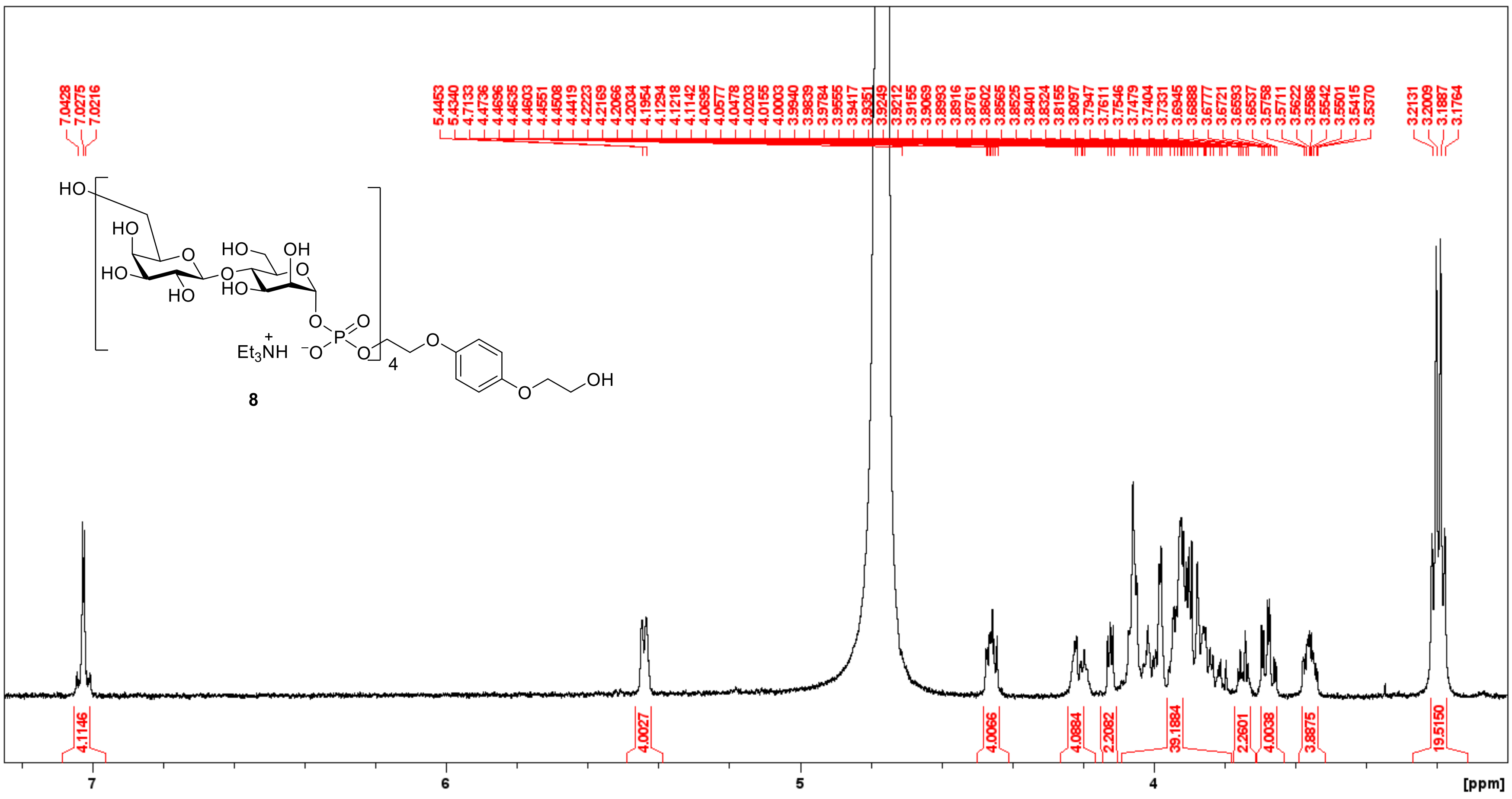

^{1}H NMR (600 MHz, D_2O)

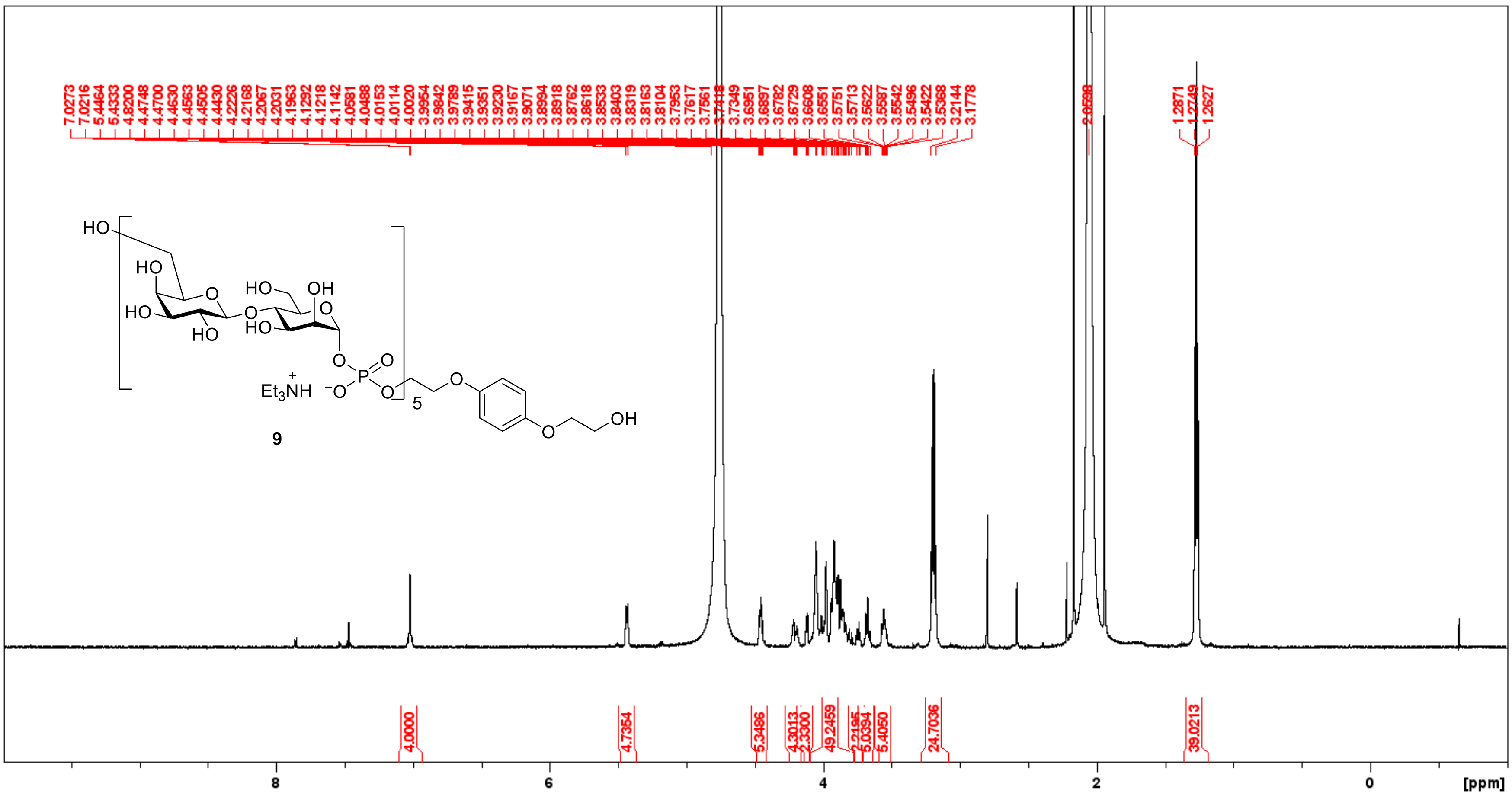

^{1}H NMR (600 MHz, D_2O, Zoom)

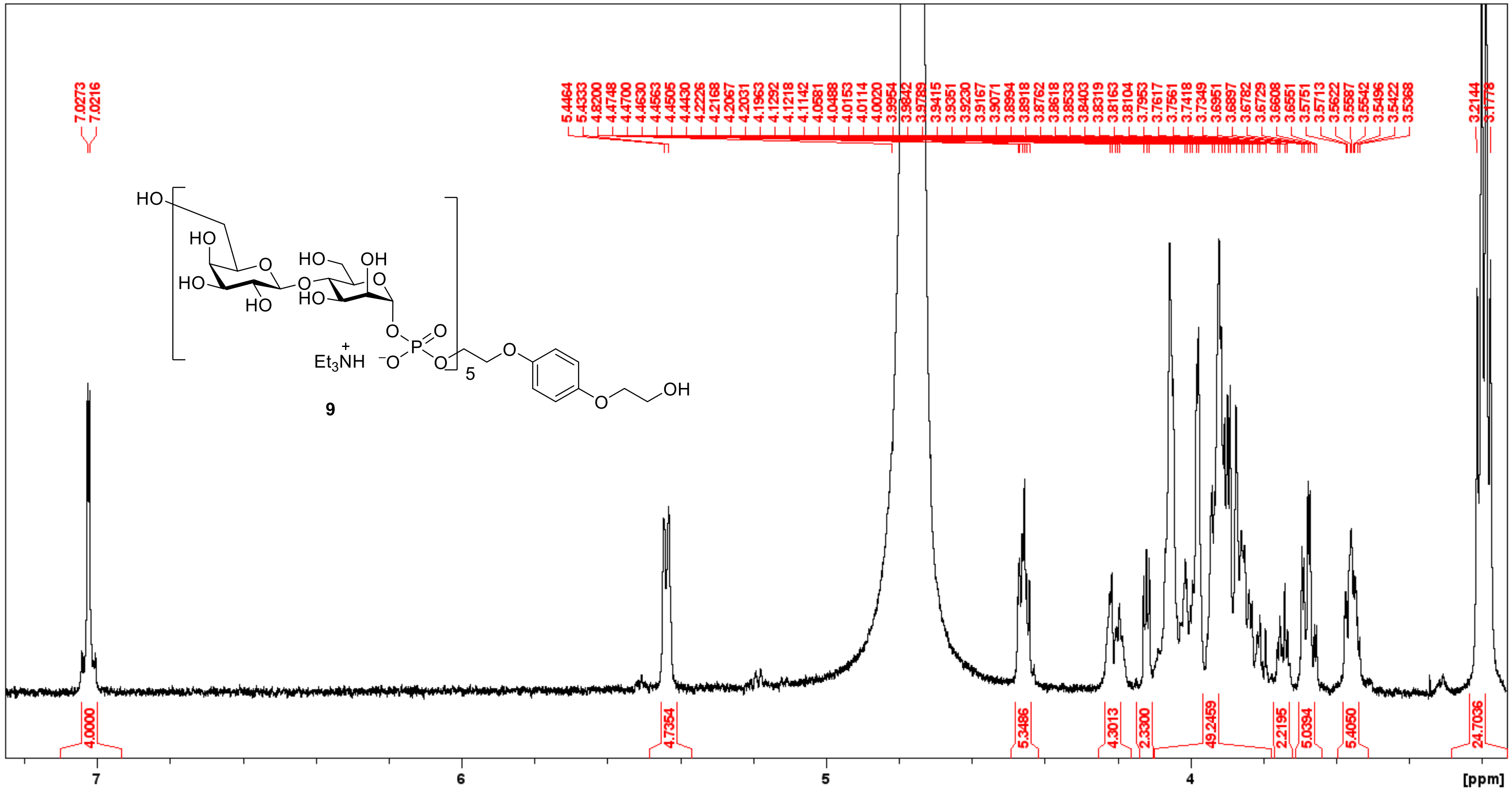

Supplement: Supplementary file 1 — ol3c01293_si_001.pdf [file ol3c01293_si_001.pdf]
